# Indeno[2,1‐*c*]fluorene Quasi[8]circulenes Through Intramolecular Cyclization

**DOI:** 10.1002/anie.202510583

**Published:** 2025-08-21

**Authors:** Eric Sidler, Robert Hein, Charlotte N. Stindt, Ben L. Feringa

**Affiliations:** ^1^ Stratingh Institute for Chemistry University of Groningen Nijenborgh 3 Groningen 9747 AG The Netherlands; ^2^ Organic Chemistry Institute University of Münster Corrensstraße 40 48149 Münster Germany

**Keywords:** Indenofluorenes, Nanographenes, Polycyclic aromatic hydrocarbons, Quasi[8]circulenes, Redox chemistry

## Abstract

The study of polycyclic aromatic hydrocarbons has become a cornerstone of chemical sciences, providing crucial guidance for advancing a wide range of fields. Within this context, quasi[8]circulenes and indenofluorenes (IFs) have gained considerable attention due to their rich chiroptical, optoelectronic, supramolecular, and redox properties. However, their combined integration into a single molecular framework has not yet been realized. This work demonstrates that curved indeno[2,1‐*c*]fluorene quasi[8]circulenes are easily accessible through an intramolecular cyclization of a readily available helicene diketone. The cyclization is high yielding and accompanied by an unexpected regioselective triflation that—alongside both ketones—allows orthogonal functionalization. This synthetic utility offers great potential for incorporating 8‐membered rings and formally antiaromatic *as*‐indacene moieties into complex architectures. Herein, a series of fully conjugated antiaromatic indeno[2,1‐*c*]fluorene quasi[8]circulenes were synthesized and studied spectroscopically and electrochemically, showcasing the rich properties of these rare, purely carbon‐based quasi[8]circulenes. It is shown that the studied compounds display multiple accessible redox states at mild potentials, which can be significantly altered through adequate substituents. These substituents additionally induce unprecedented aggregation behavior for one of the compounds. Furthermore, the studied IFs exhibit chirality arising from the curved structure of the quasi[8]circulenes, which allowed studying chiroptical properties for one of the structures.

## Graphical abstract

A new class of curved, chiral polycyclic aromatic hydrocarbon emerges from the fusion of indeno[2,1‐*c*]fluorene and quasi[8]circulene motifs. These redox‐active, antiaromatic scaffolds offer great synthetic utility, tunable properties, and modular functionalities for organic electronics and functional materials.

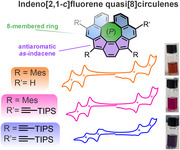

## Introduction

**Figure 1:**
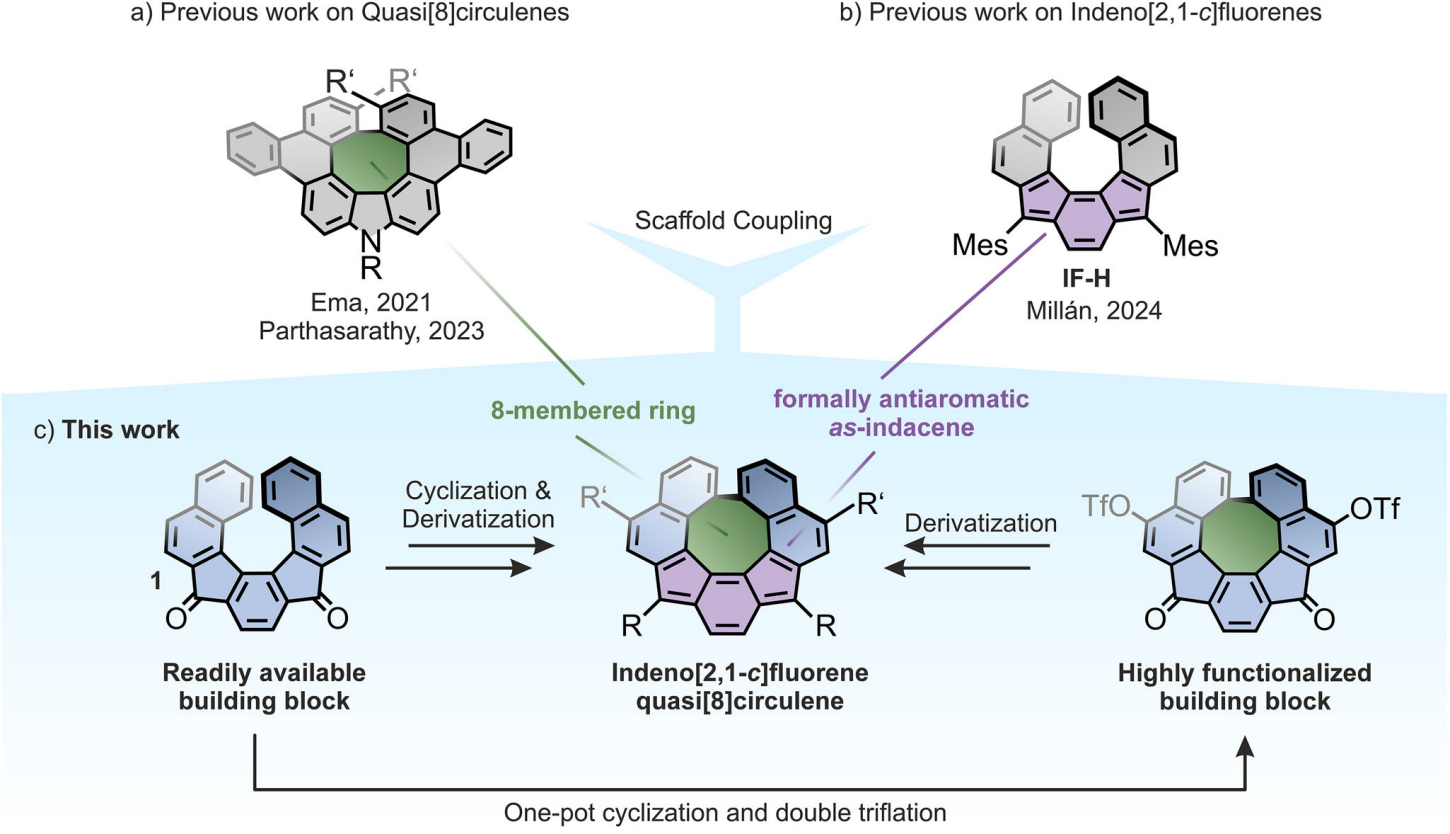
a) Selected recently reported quasi[8]circulene. b) Previously reported helical indeno[2,1‐*c*]fluorene (**IF‐H**). c) Overview of the present work wherein a series of unique all‐carbon indeno[2,1‐*c*]fluorene quasi[8]circulenes are obtained via highly functionalized building blocks.

Celebrating the 200‐year discovery of benzene,^[^ ^1^ ^]^ studies on polycyclic aromatic hydrocarbons (PAHs) have risen up to be one of the core pillars of organic chemistry, progressing not only fundamental sciences^[^ ^2^ ^]^ but also providing crucial answers for advancing technological innovations and industries.^[^ ^3^ ^]^ PAHs are utilized as molecular switches,^[^ ^4^ ^]^ dyes,^[^ ^5^ ^]^ luminophores,^[^ ^6^ ^]^ organic semiconductors^[^ ^7^ ^]^ or photosensitizers,^[^ ^8^ ^]^ and play a central role in organic light‐emitting diodes (OLEDs),^[^ ^9^ ^]^ organic photovoltaics (OPVs),^[^ ^10^ ^]^ molecular electronics,^[^ ^11^ ^]^ supramolecular assemblies,^[^ ^12^ ^]^ molecular machines,^[^ ^13^, ^14^ ^]^ optoelectronics,^[^ ^15^ ^]^ and chiroptical applications,^[^ ^16^ ^]^ just to name a few. Within the context of PAHs, nanographenes have gained significant attention due to their intriguing charge carrier mobilities, tunable optoelectronic properties and more facile accessibility and processability than the parent graphene.^[^ ^17^, ^18^, ^19^ ^]^ Recently, introduction of curvature into the backbone of nanographenes has led to the development of curved aromatics, which display narrower bandgaps, improved solubility and processability, along with prevalent inherent chirality.^[^ ^20^, ^21^, ^22^, ^23^, ^24^ ^]^ A particularly intriguing example is the class of [*n*]circulenes, where *n* ortho‐fused aromatic rings encircle an *n*‐membered ring.^[^ ^25^, ^26^ ^]^ Through opening of one of the surrounding rings, curved structures, so‐called quasi[*n*]circulenes are obtained. Among them, quasi[8]circulenes, wherein seven fused rings are flanking an 8‐membered ring, have gained considerable attention recently due to their strong curvature induced by the nonaromatic and nonplanar central octagon and steric clash of the ortho‐substituents adjacent to the connecting single biaryl bond (Figure 1a). These structural features endow quasi[8]circulenes with intriguing (chir)optoelectronic and redox properties, as well as host–guest chemistry. The first quasi[8]circulene was reported in 2009,^[^ ^27^ ^]^ and only a limited number of such structures have been described since.^[^ ^28^, ^29^ ^]^ Especially purely carbon‐based analogues remain rare and are either found as low‐yielding products of a nonselective reaction^[^ ^30^ ^]^ or as fragments of larger polycyclic frameworks.^[^ ^31^, ^32^ ^]^ In contrast, most reported structures typically incorporate heterocycles in the periphery of the 8‐membered ring. For example, Osuka, Pittelkow, and Iwanaga documented the quasi[8]circulenes with three heterocycles, highlighting their interesting optoelectronic and chiral properties.^[^ ^33^, ^34^, ^35^, ^36^ ^]^ Similarly, Ema and Parthasarathy separately reported on quasi[8]circulenes comprising only a single pyrrole heterocycle surrounding the central ring,^[^ ^37^, ^38^ ^]^ and Takizawa described the electrochemical syntheses of a quasi[8]circulene bearing a furane and pyrrole moiety.^[^ ^39^, ^40^ ^]^ For most quasi[8]circulene compounds, however, the synthesis involves intramolecular cyclization reactions on helicene precursors, wherein the 8‐membered ring is formed using *Scholl*‐type reaction conditions as the final step. This may not only be faced with low chemo‐ and regioselectivity but may also suffer from a narrow scope due to potential over‐oxidation and limited functional group tolerance that hampers further derivatizations.

Amid the plethora of polycyclic conjugated structures, indenofluorenes (IFs) make up an intriguing class of compounds that display a formally antiaromatic indacene scaffold in their fully conjugated state (purple rings in Figure 1b).^[^ ^41^, ^42^, ^43^ ^]^ This results in a significant diradical character, amphoteric redox behavior, and narrow bandgaps, providing them with promising properties for organic semiconductor applications. Among the five possible regioisomers of IFs, the fully conjugated formally antiaromatic indeno[2,1‐*c*]fluorene, which was first synthesized in 2013 by Haley and coworkers,^[^ ^44^ ^]^ displays the lowest diradical character and thus typically the highest stability. Recently, the helical diketone **1** (Figure 1c) and dihydro derivatives, comprising an indeno[2,1‐*c*]fluorene scaffold in its structure, have gained attraction for the development of circularly polarized luminescence (CPL) emitters,^[^ ^45^, ^46^ ^]^ configurationally stable chiral IFs (**IF‐H**, Figure 1b)^[^ ^47^ ^]^ or as starting points to access various different architectures.^[^ ^48^ ^]^ We have demonstrated the use of **1** as a building block for a redox‐driven aromaticity switch, wherein the simultaneous oxidation of redox‐active rotors allows the reversible formation of the antiaromatic indacene moiety in the IF scaffold.^[^ ^49^ ^]^ Building on previous methodologies of intramolecular cyclizations of helical precursors toward quasi[8]circulenes, an investigation into the reactivity of the helical diketone **1** was initiated. Herein, we report the successful realization of an intramolecular cyclization that affords a structurally unprecedented fusion of quasi[8]circulene and indeno[2,1‐*c*]fluorene motifs and one of the rare examples of purely carbon‐based quasi[8]circulenes. Furthermore, it is demonstrated that the intramolecular cyclization is accompanied by an unusual triflation that allows further transformations using cross‐coupling reactions. The practicality of the reported structures is showcased through subsequent derivatization that led to a series of highly functionalized IFs, which were comprehensively characterized by spectroscopic and electrochemical methods, complemented by density functional theory (DFT) calculations.

## Results and Discussion

### Synthesis

**Figure 2:**
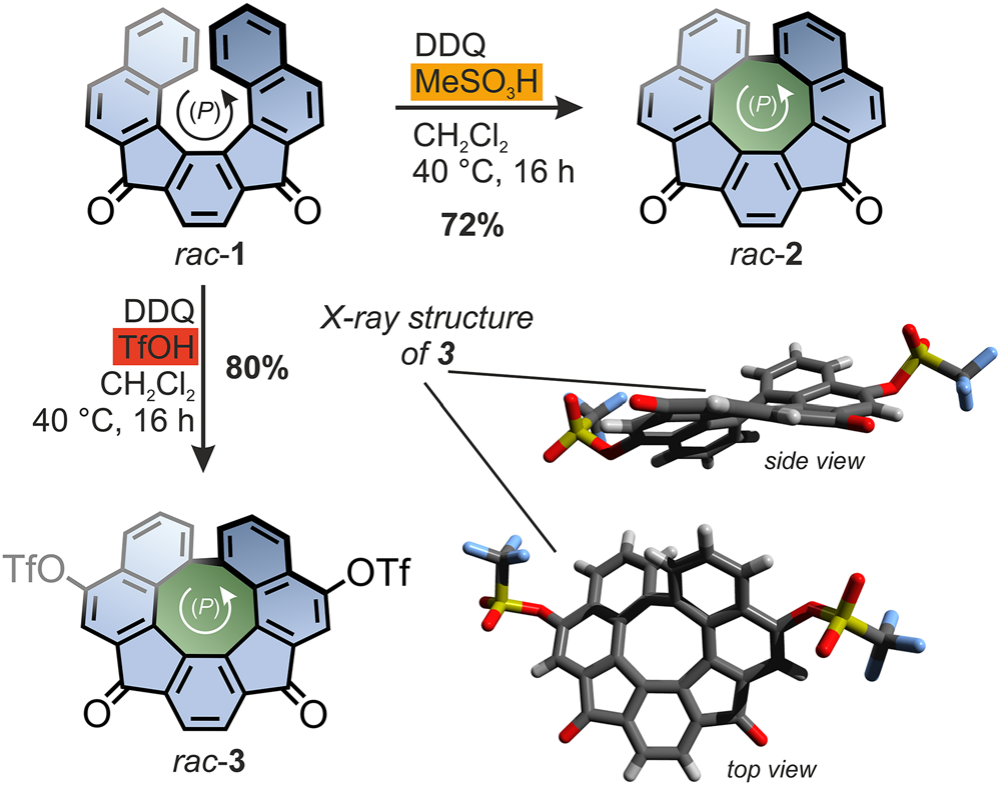
Overview of the synthetic route toward *rac*‐**2** and *rac*‐**3** starting from *rac*‐**1**, as well as top and side views of (*P*)‐**3** in the crystal structure of *rac*‐**3**.

The helically chiral indeno[2,1‐*c*]fluorene diketone *rac*‐**1** was synthesized in a racemic fashion according to a modified literature procedure in 51% yield (1 g scale) from a readily available building block that can be obtained using a few high‐yielding reaction steps.^[^ ^45^, ^46^ ^]^ Initially, subjecting racemic *rac*‐**1** to standard *Scholl* oxidation conditions^[^ ^50^ ^]^ using 1 equiv. of 2,3‐dichloro‐5,6‐dicyano‐1,4‐benzoquinone (DDQ) in a mixture of dichloromethane and methanesulfonic acid (10:1) at 0 °C resulted in the formation of a mixture of starting material *rac*‐**1** and desired cyclized indeno[2,1‐*c*]fluorene quasi[8]circulene diketone *rac*‐**2** in a ratio of 15:85. Only a subsequent reaction with additional DDQ resulted in the complete conversion to *rac*‐**2**. Conveniently, subjecting *rac*‐**1** immediately to an excess of DDQ (7 equiv.) and heating the mixture to 40 °C overnight resulted in the complete conversion to *rac*‐**2** without the formation of significant amounts of side products. Isolation through simple flash column chromatography afforded *rac*‐**2** in 72% yield (Figure 2).

**Figure 3:**
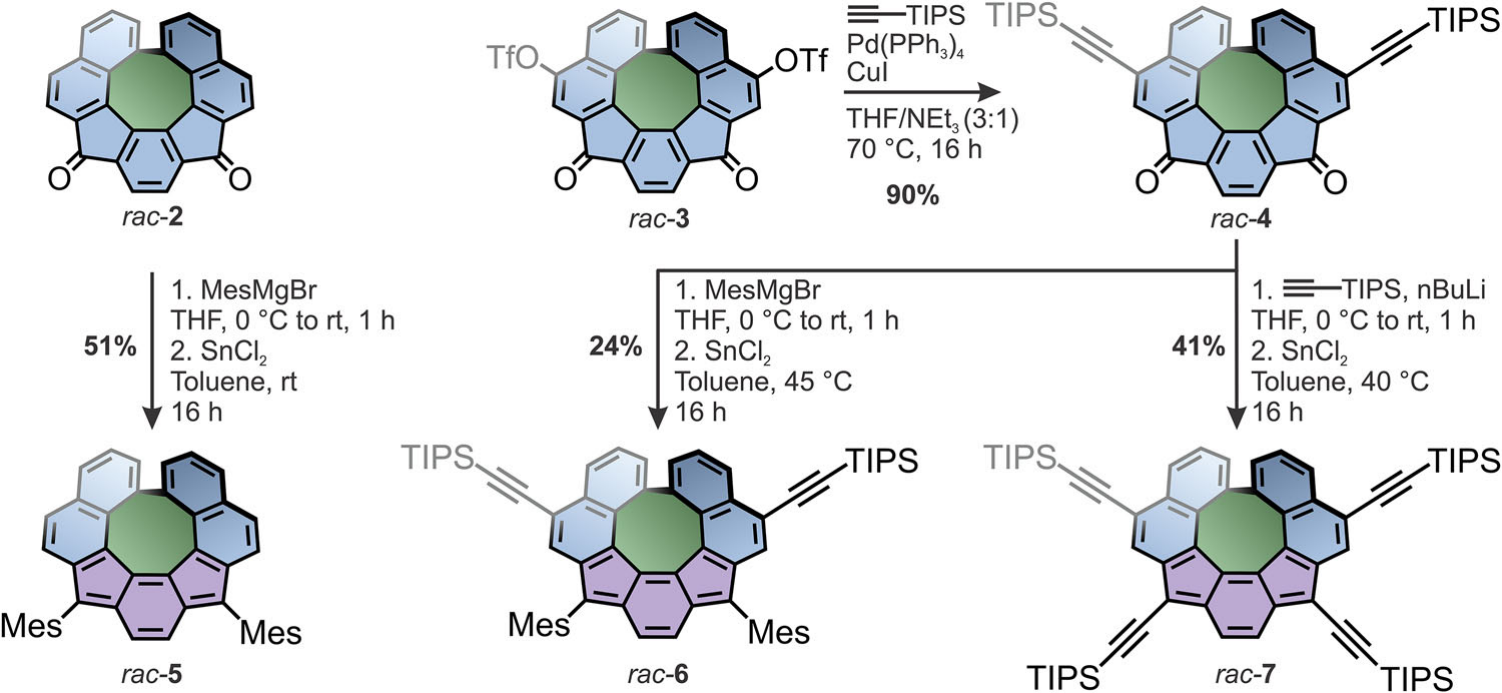
Overview of the synthetic route toward *rac*‐**4**, *rac*‐**5**, *rac*‐**6**, and *rac*‐**7** starting from *rac*‐**2** and *rac*‐**3**.

Unexpectedly, when the reaction was performed with triflic acid (TfOH) instead of methanesulfonic acid, it resulted in the formation of a distinct species. Aside from the desired ring closure, a regioselective double triflation was observed, yielding the highly functionalized quasi[8]circulene *rac*‐**3** in a good yield of 80% as the sole product (Figure 2). This is particularly interesting, considering that reports on such aromatic substitution reactions using acid and DDQ remain rare,^[^ ^51^, ^52^, ^53^ ^]^ even though they represent widely employed conditions for *Scholl* reactions on countless nanographenes and other PAHs.^[^ ^54^, ^55^ ^]^ The formation of **3** was unambiguously verified by X‐ray diffraction of suitable single crystals that were obtained via pentane vapor diffusion into a solution of *rac*‐**3** in CH_2_Cl_2_ (Figure 2). In the crystal structure, the central 8‐membered ring displays a significant distortion from planarity, which allows its escape from the antiaromatic penalty. Furthermore, the structure suggests strong steric clashes of the protons adjacent to the newly formed biaryl bond, which hints at configurational stability of the helical chirality (vide infra). The unit cell (space group *P*2_1_/*c*) contains two molecules of (*P*)‐**3** and (*M*)‐**3** each. Two adjacent molecules feature phenylene rings in close proximity (up to 3.63 Å), indicating efficient π–π stacking (Figure S14). In total, compound *rac*‐**3** features four handles for further functionalization, two triflates and two ketone moieties, which makes it an intriguing starting point for the integration of curved 8‐membered rings and formally antiaromatic IF scaffolds into various functionalized nanographene‐type structures and larger assemblies. The integrity and reactivity of the triflates as *pseudo*‐halides was confirmed by subjecting *rac*‐**3** to standard *Sonogashira* conditions using (triisopropylsilyl)acetylene (TIPSA), Pd(PPh_3_)_4_, and CuI in a mixture of THF and triethylamine (3:1) at elevated temperatures of 70 °C. This allowed isolation of well‐soluble *rac*‐**4** in a high yield of 90%. Starting from *rac*‐**2** and *rac*‐**4**, a small series of conjugated formally antiaromatic indeno[2,1‐*c*]fluorene quasi[8]circulenes (*rac*‐**5**, *rac*‐**6**, and *rac*‐**7**) was then prepared following conventional literature procedures for IFs (Figure 3).^[^ ^44^, ^56^, ^57^ ^]^ Commercially available 2‐mesitylmagnesium bromide (MesMgBr) was employed to undergo 1,2‐addition on the diketones of both *rac*‐**2** and *rac*‐**4**, followed by immediate reductive elimination of the crude diols using tin(II)chloride to afford *rac*‐**5** and *rac*‐**6** in 51% and 24% yield, respectively. Similarly, *rac*‐**7** was obtained in 41% yield via a nucleophilic addition by in situ generated lithium TIPS‐acetylide on *rac*‐**4**, followed by reductive elimination in toluene. For the synthesis of both *rac*‐**6** and *rac*‐**7**, the reductive elimination of the diol intermediates required elevated temperatures of 45 and 40 °C, respectively, whereas room temperature was sufficient for the formation of *rac*‐**5**. The reactivity difference of the intermediate diol species can be attributed to the increased steric hindrance caused by the presence of large TIPSA substituents in *rac*‐**6** and *rac*‐**7** that were previously installed on *rac*‐**4**.

### Spectroscopy

**Figure 4:**
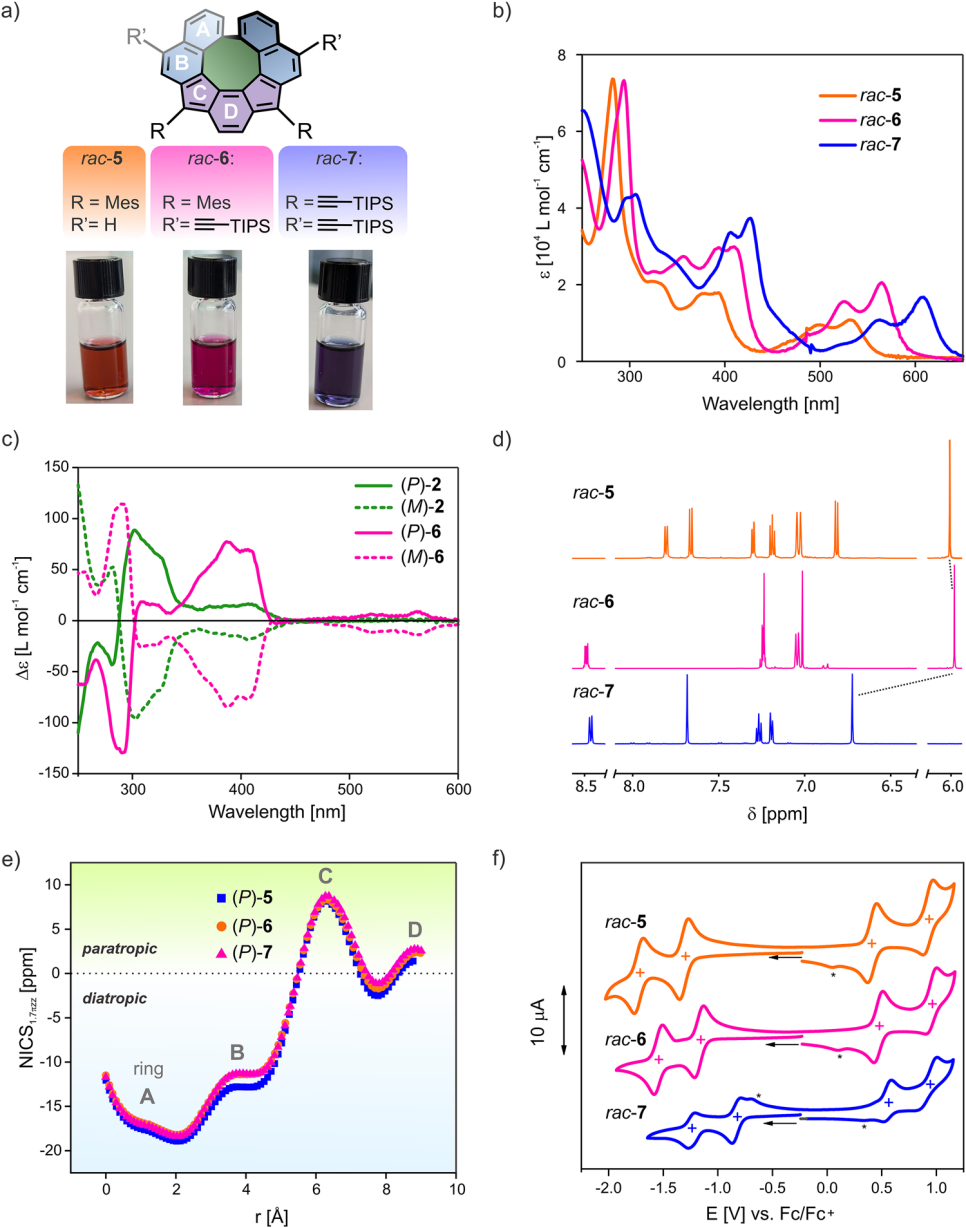
a) Molecular structure of *rac*‐**5**, *rac*‐**6**, and *rac*‐**7** and the color of their respective dilute solutions in CH_2_Cl_2_. b) Absorption spectra of *rac*‐**5** (orange), *rac*‐**6** (pink), and *rac*‐**7** (blue). c) CD spectra of the (*P*)‐enantiomers (solid lines) and (*M*)‐enantiomers (dashed lines) of **2** (green) and **6** (pink) in CH_2_Cl_2_. d) Downfield shifted region of the ^1^H NMR spectra (600 MHz, CD_2_Cl_2_, 298 K) of *rac*‐**5** (orange), *rac*‐**6** (pink), and *rac*‐**7** (blue). e) NICS_1.7πzz_‐XY scan of *rac*‐**5** (orange), *rac*‐**6** (pink), and *rac*‐**7** (blue), obtained via DFT calculations. f) CVs of 0.5 mM *rac*‐**5** (orange), *rac*‐**6** (pink), and *rac*‐**7** (blue) at *ν* = 100 mV s^−1^ in CH_2_Cl_2_, 100 mM TBAPF_6_. The black arrow indicates the starting point and direction of the first scan. The peaks marked with an asterisk are not impurities but arise as a result of the second oxidation wave (and second reduction for *rac*‐**7**).

All three isolated IFs (*rac*‐**5** to *rac*‐**7**) are strongly colored compounds due to the presence of a large π system as well as a conjugated formally antiaromatic *as*‐indacene moiety (purple scaffold in Figure 3). This can be seen in dilute CH_2_Cl_2_ solutions (*c* ∼ 10^−5^ M), which display orange, pink, and deep blue colorations for *rac*‐**5**, *rac*‐**6**, and *rac*‐**7**, respectively (Figure 4a). The intense colors of the IFs can also be deduced from the UV–vis spectra which display strong transitions well into the visible range, in line with previously reported conjugated IFs (Figure 4b).^[^ ^43^, ^44^, ^47^ ^]^ For all three compounds, the most red‐shifted region is governed by three main transitions, which are continuously redshifted in correlation with the number of TIPSA substituents attached to the molecular framework. Whereas *rac*‐**5** displays a *λ*_max _= 530 nm (zero TIPSA groups), *rac*‐**6** (two TIPSA groups) and *rac*‐**7** (four TIPSA groups) display *λ*_max_ of 565 and 609 nm, respectively. The consecutive bathochromic shift upon introduction of more TIPSA substituents is also in good agreement with an increased redox amphotericity, as revealed by electrochemical studies (vide infra). Notably, compared to *rac*‐**1**, *rac*‐**2**, and *rac*‐**4** (Figure S21), all IF derivatives display a significantly more red‐shifted absorption spectrum, further confirming the presence of the conjugated *as*‐indacene moiety. Interestingly, while the archetypal indeno[2,1‐*c*]fluorene,^[^ ^44^ ^]^ the helicene‐based **IF‐H** ^[^ ^47^ ^]^ (Figure 1b) and other benzo‐fused indeno[2,1‐*c*]fluorenes^[^ ^57^ ^]^ display a very weak and broad absorption band corresponding to the HOMO–LUMO transition, only *rac*‐**5** showcases a similar response at 750 nm (Figure S22). In contrast, in both *rac*‐**6** and *rac*‐**7**, this broad transition is absent. Time‐dependent density functional theory (TD‐DFT) at the CAM‐B3LYP/def2‐TZVP/CPCM(CH_2_Cl_2_)^[^ ^58^, ^59^, ^60^ ^]^ level of theory revealed weak oscillator strengths of *f* = 0.057, 0.053, and 0.065 for *rac*‐**5**, *rac*‐**6**, and *rac*‐**7** at 770, 883, and 946 nm, respectively (Figures S47–S49). This absence may thus stem from subtle differences in molecular geometry or electronic distribution not fully covered by the computed oscillator strengths. This suggests that other factors besides the calculated oscillator strengths, such as vibronic coupling or conformational effects, may influence the visibility of this transition in the experimental spectra. Similarly to other reported conjugated IFs, compounds *rac*‐**5** to *rac*‐**7** are nonluminescent.^[^ ^61^ ^]^

### Aggregation Studies

Interestingly, when dissolving the green solid *rac*‐**7** in organic solvents, peculiar time‐dependent color changes were observed. Initially, the solution was endowed with a deep green coloration, which over time transitioned into deep blue (Figure 4a). This color change (from green to blue) could be accelerated by diluting the samples, which is consistent with the breakup of an initial aggregate (green) into its monomeric species *rac*‐**7** (blue). This was investigated by measuring the absorption spectra of *rac*‐**7** in THF/water mixtures (Figure S16). Indeed, when reaching a 50% fractional volume of water, all three transitions governing the most red‐shifted absorption spectrum of *rac*‐**7** basically completely disappeared or were considerably blueshifted. By increasing the fractional volume of water even more, only negligible additional differences were observed. To get further insights into the aggregation behavior, a diffusion‐ordered spectroscopy NMR (DOSY‐NMR) spectrum of a concentrated sample of *rac*‐**7** was measured. The sharp signals of the monomeric species of *rac*‐**7** were accompanied by broad signals of a slower diffusing, higher molecular weight species, suggesting the formation of aggregates (Figure S15). Furthermore, the color of the sample remained green and did not transition to blue over time, reminiscent of the initial color that is observed when dissolving the solid compound in organic solvents. Analysis of the DOSY experiment using the Stokes–Einstein–Sutherland equation allowed us to determine a hydrodynamic radius of 3.17 and 7.31 Å for the monomeric and the broad aggregate species, respectively, suggesting the exclusive formation of a discrete dimeric aggregate. A rough estimate of the dimerization constant of *K*_dim_ = 7.6 x 10^4^ M^−1^ in THF/water (6:4) was determined using UV–vis spectroscopy (see Figures S17 and S18 and associated discussion). The true nature of the aggregate, and especially the nature of the bonding, remains elusive at this point and requires further detailed attention. However, the significant hypsochromic changes in the UV–vis spectrum in the aggregate state might already hint at the possibility that the formally antiaromatic *as*‐indacene moiety is dimerizing to form an overall aromatic or nonaromatic state. While this phenomenon has not been reported for IFs, similar dynamics have been observed for other diradicaloids^[^ ^62^ ^]^ and investigations regarding the nature of aggregation of *rac*‐**7** are currently ongoing. The diradical character *y*_0_ was calculated by determining the occupation numbers of the natural orbitals using unrestricted DFT,^[^ ^63^, ^64^ ^]^ which gave values of *y*_0_ **= 0 or close to zero for all the studied IFs (Table S4). This suggests that the dimerization of *rac*‐**7** is not predominantly driven by diradical interactions since none of the other species, *rac*‐**5** or *rac*‐**6**, displayed similar aggregation behavior, neither in NMR nor UV–vis measurements, even though comparable *y*_0_ were computed.

### Chiral Analysis

Similarly to other quasi[8]circulenes, the steric clashes of the biaryl bond forming the 8‐membered ring, along with its nonplanarity render these structures inherently chiral, and possibly allows separation of their (*M*)‐ and (*P*)‐enantiomers. The chiral resolution using high‐performance liquid chromatography (HPLC) on a chiral stationary phase was first investigated for quasi[8]circulene diketone *rac*‐**2**. Conveniently, analytical and semipreparative separation of *rac*‐**2** into (*M*)‐**2** and (*P*)‐**2** was achieved using a CHIRALPAK IG column and pure CH_2_Cl_2_ as eluent, which facilitated the separation owing to the decent solubility of *rac*‐**2** in CH_2_Cl_2_. In contrast, enantiomeric separation of the IF derivatives proved more troublesome and only *rac*‐**6** could be resolved using a CHIRALPAK IBN‐5 column and pure *n*‐heptane as eluent. Notably, already with a column oven temperature of 40 °C, a slight plateau between the two enantiomeric chromatogram peaks was observed, indicating a low racemization barrier (Figure S20). This was much more notable when performing the separation at 70 °C column oven temperature, where a broad and intense plateau between the peaks was observed (Figure S28). The elution profile was then analyzed via the unified equation for dynamic chromatography,^[^ ^65^ ^]^ which enabled determination of an enantiomerization barrier of $\Delta G_{e}^{‡\;}$ = 99 kJ mol^−1^ for **6** at 70 °C, corresponding to a half‐life time at room temperature of *t*_1/2_ = 14 h. On the other hand, the enantiomerization barrier of **2**, which was determined by dynamic circular dichroism (CD) spectroscopy at 60 °C, revealed a $\Delta G_{e}^{‡}$ = 108 kJ mol^−1^, which amounts to a half‐life time of *t*_1/2_ = 23.5 d at room temperature (Figure S27).^[^ ^66^ ^]^ The enantiomerization barriers were further investigated using DFT at the r^2^SCAN‐3c/CPCM(CH_2_Cl_2_)^[^ ^60^, ^67^ ^]^ level of theory by calculating the transition state energies (Figure S44). Indeed, DFT predicts a barrier of $\Delta G_{e}^{‡}$ = 110 kJ mol^−1^ for **2**, which is in good agreement with the experimental value of 108 kJ mol^−1^. Albeit slightly lower than the experimental value, DFT predicts a barrier of $\Delta G_{e}^{‡}$ = 94 kJ mol^−1^ for **6**, which, however, is in line with the general trend of an overall lower barrier than for **2**. It should be noted that the comparison of the enantiomerization barriers obtained via dynamic chromatography with DFT calculations should be interpreted with caution, as the enantiomerization mechanism on the surface of the solid phase of the chiral column can differ considerably. For **5**, DFT predicts an even lower barrier of $\Delta G_{e}^{‡}$ = 92 kJ mol^−1^, which explains why separation into the respective enantiomers was not achieved. On the other hand, DFT suggests a barrier of $\Delta G_{e}^{‡}$ = 100 kJ mol^−1^ for **7**, similarly to **6**. However, no separation on various chiral columns was achieved, possibly due to the apolar nature of the compound, which typically resulted in elution of **7** along with the injection peak, even when using pure *n*‐heptane as eluent. In general, the presence of the conjugated and antiaromatic (vide infra) *as*‐indacene moiety seems to lower the enantiomerization barrier of these quasi[8]circulenes relative to the diketone precursor **2**. Compared to previously reported quasi[8]circulene structures (without substituents next to the biaryl bond), the herein studied compounds display very similar enantiomerization barriers.^[^ ^28^, ^34^ ^]^ The CD spectra of (*M*)‐**2**/(*P*)‐**2** and (*M*)‐**6**/(*P*)‐**6** (Figures 4c, S23, and S25) display decently strong Cotton bands with Δ*ε*_max_ (244 nm) = 168 L mol^−1^ cm^−1^ for (*M*)‐**2** and Δ*ε*_max_ (290 nm) = −130 L mol^−1^ cm^−1^ for (*P*)‐**6**. Relative to the enantiopure **IF‐H**,^[^ ^47^ ^]^ compound **6** exhibits much more pronounced Cotton effects, with Δ*ε* values exceeding those of the former by more than a factor of five for analogous transitions. In the red‐shifted region of the spectrum, **6** also displays weaker Cotton band intensities, mirroring the behavior observed for **IF‐H**. The enantiopure samples furthermore display average absorption dissymmetry factors (*g*_abs_ = Δ*ε*/*ε*) in the range of 10^−3^, similarly to other chiral small molecules and **IF‐H** (Figures S24 and S26). Assignment of the absolute configuration was performed by comparing the experimental with simulated CD spectra obtained from TD‐DFT calculations at the CAM‐B3LYP/def2‐TZVP/CPCM(CH_2_Cl_2_) level of theory for (*P*)‐**2** and (*P*)‐**6** (Figures S45 and S46).

### NICS Calculations

Next, the antiaromatic character of the conjugated *as*‐indacene moieties for all IF derivatives was analyzed. Already in the respective ^1^H NMR spectra, the protons of the central phenylene unit of the *as*‐indacene scaffold displays an upfield shift with respect to the other aromatic signals. More precisely, the protons of the central ring displays a resonance signal at 5.97, 5.94, and 6.68 ppm for *rac*‐**5**, *rac*‐**6**, and *rac*‐**7**, respectively (Figures 4d, S8, S10, and S12), in line with other reported IF compounds. On previously DFT‐optimized structures at the r^2^SCAN‐3c/CPCM(CH_2_Cl_2_) level (Figure S43 and Section 9 in the Supporting Information), NICS_1.7πzz_‐XY scans^[^ ^68^, ^69^, ^70^ ^]^ at the B3LYP/6‐311+G*/NMR = GIAO^[^ ^71^, ^72^, ^73^ ^]^ level of theory were performed to investigate magnetically induced ring currents. The NICS‐XY scan plots (Figure 4e) reveal strong aromatic (diatropic) character for rings A and B (see Figure 4a for ring denotation) and antiaromatic (paratropic) character for rings C and D, reaching values comparable to previously reported benzo‐fused indeno[2,1‐*c*]fluorene systems.^[^ ^57^ ^]^ For symmetry reasons, only half of the molecule was scanned and within the series of studied IF compounds, only negligible differences can be observed in the NICS‐XY plots. Also, the magnetically induced ring currents of the 8‐membered ring were investigated by calculating the NICS(1.7) values at the center of the ring, which amounted to 0.22, 1.09, and 1.12 ppm for *rac*‐**5**, *rac*‐**6**, and *rac*‐**7**, respectively, indicating negligible induced ring currents.

### Electrochemistry

Due to their antiaromatic nature, IF derivatives display rich redox properties, characterized by multiple, typically reversible, reductions/oxidations and a high degree of redox amphotericity.^[^ ^43^, ^47^, ^57^, ^74^, ^75^ ^]^ To probe this, as well as the influence of ring closure of the novel IF quasi[8]circulenes, *rac*‐**5**, *rac*‐**6**, and *rac*‐**7** were systematically studied via cyclic voltammetry (CV) and square‐wave voltammetry (SWV) in CH_2_Cl_2_. As shown in Figure 4f, for all three compounds four one‐electron redox couples were resolved, corresponding to two reductions (IF^2−/−•^ (Red2) and IF^−•/0^ (Red1)) and two oxidations (IF^0/+•^ (Ox1) and IF^+•/2+^ (Ox2)), respectively (see also Figures S29 and S30). For *rac*‐**5**, these interconversions occur at −1.720, −1.305, +0.410, and +0.925 V (versus Fc/Fc^+^), respectively (Table 1). This behavior is similar to the helical, noncyclized analogue **IF‐H**,^[^ ^47^ ^]^ which also displays four quasi‐reversible redox couples, albeit shifted to lower potentials by 40–130 mV. This indicates that cyclization to the quasi[8]circulene *rac*‐**5** is associated with a slight increase in all oxidation potentials but does otherwise not significantly affect the redox properties of the system.

**Table 1:** Half‐wave potentials *E*_1/2_ of *rac*‐**5**, *rac*‐**6**, and *rac*‐**7** in CH_2_Cl_2_, 100 mM TBAPF_6_ versus Fc/Fc^+^.

	Red2 (V)	Red1 (V)	Ox1 (V)	Ox2 (V)	*E*_LUMO_ ^c)^ (eV)	*E*_HOMO_ ^d)^ (eV)	*E*_gap_ (eV)
*rac*‐**5**	−1.720^a)^	−1.305^a)^	+0.410^a)^	+0.925^b)^	−3.56	−5.15	1.59
*rac*‐**6**	−1.550^a)^	−1.165^a)^	+0.470^a)^	+0.955^b)^	−3.69	−5.20	1.51
*rac*‐**7**	−1.245^b)^	−0.830^a)^	+0.555^b)^	+0.935^b)^	−4.03	−5.27	1.24

^a)^ Reversible.

^b)^ Quasireversible; the reported potentials correspond to the peak potentials obtained via SWV.

^c)^ *E*_LUMO_ = –(4.8 eV + *E*_Red1, onset_).

^d)^ *E*_HOMO_ = –(4.8 eV + *E*_Ox1, onset_).

**Figure 5:**
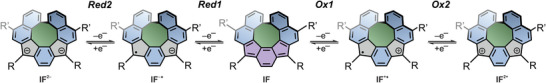
Depiction of the five charge states of the IF derivatives and their proposed, most relevant mesomeric structures.

Upon introduction of two additional TIPSA groups in the R’ position (Figure 4a), the redox potentials of all couples of *rac*‐**6** shift anodically wrt. *rac*‐**5** (Table 1 and Tables S2, S3), in good agreement with the electron‐withdrawing nature of the TIPSA motifs. This effect is larger for the reductive couples (140–170 mV shift) than for the oxidative couples (30–60 mV shift), indicating that the LUMO energy is more significantly lowered than the HOMO energy by this modification (Table 1). An even larger effect is observed for *rac*‐**7**, where the mesityl groups are also substituted for TIPSA motifs. In this case, additional anodic shifts of 305 and 335 mV are observed for the second and first reduction, respectively, while this effect is less pronounced for the oxidations. The significantly larger effect of the substituents at the IF core (R‐position) can be explained by considering the mesomeric structures of the different charge states (Figure 5), whereby the R‐group is directly attached to the position in which the charge localization is expected to be highest.

Of particular note is also the low difference between the first oxidation and reduction potentials Δ*E*_Ox1‐Red1_ for all compounds, confirming a high degree of redox amphotericity. For example, Δ*E*_Ox1‐Red1_ is 1.715 V for *rac*‐**5**, which is slightly smaller than the value reported for **IF‐H** (1.750 V).^[^ ^47^ ^]^ For *rac*‐**6** and *rac*‐**7**, this gap shrinks even further to 1.635 and 1.385 V, respectively, a trend that is also in good agreement with the progressively smaller HOMO–LUMO gaps as evidenced by increasingly red‐shifted absorbances of this series of derivatives (Figure 4b).

Finally, the redox processes of these novel IF quasi[8]circulenes are diffusion‐controlled and predominantly display a high degree of redox reversibility as evidenced by a near‐unity ratio of peak currents, a low peak separation Δ*E*_p_ (Table S2) and a linear dependence of the peak currents on the square root of the scan rate across a large range of scan rates, as predicted by the Randles–Sevcik equation, see Figures S31–S42. This is particularly true for Red2, Red1, and Ox1 for *rac*‐**5** and *rac*‐**6** as well as Red1 for *rac*‐**7** with Δ*E*_p_ < 77 mV in all cases. Ox2 is quasi‐reversible for all three derivatives; in particular for *rac‐* **7** the reversibility is more limited, especially for Red2 and Ox2, which might, at least in part, be related to the aggregation behavior (vide supra). Nevertheless, in all cases at least three stable/reversible redox states are accessible (IF^−•/0/+•^ for *rac*‐**7**), while for *rac*‐**5** and *rac*‐**6** the dianion (and to a lesser extent the dication) are also stable. Such a large number of accessible redox states is rare for all‐carbon‐based molecules but is expected to enable further redox switching of, for example, (chir)optical properties of these intriguing nonplanar π‐systems.

## Conclusion

In this study, a novel combination of quasi[8]circulene and indeno[2,1‐*c*]fluorene within the same molecular scaffold was prepared and investigated for the first time. While the synthesis of polycyclic structures comprising 8‐membered rings is typically challenging,^[^ ^76^ ^]^ the herein presented oxidative cyclization of the helicene precursor **1** leads to the formation of the central 8‐membered ring in **2** in high yields. Notably, performing the *Scholl* cyclization in presence of TfOH additionally leads to a regioselective and unexpected triflation, affording compound **3**, providing a novel route to functionalized quasi[8]circulenes. Starting from **2** and **3**, a series of fully conjugated formally antiaromatic IF quasi[8]circulenes (*rac*‐**5**, *rac*‐**6**, and *rac*‐**7**) were synthesized and investigated. These compounds retain the characteristic redox activity of IFs, featuring multiple accessible redox states while also demonstrating that structural modification through peripheral substitution can significantly tune their optical and electronic properties, including color, HOMO–LUMO gap, redox amphotericity, and aggregation behavior. Although the compounds exhibit relatively low enantiomerization barriers, the inherent curvature of the quasi[8]circulene framework imparts chirality to the IFs, potentially enabling future exploration of spin‐selective redox processes based on their rich redox behavior. Finally, the highly functionalized intermediate **3**, bearing intrinsic chirality, an 8‐membered ring, and four reactive sites (two triflates and two ketones), offers substantial potential as a modular building block for the construction of complex polycyclic aromatic and antiaromatic architectures.

## Conflict of Interests

The authors declare no conflict of interest.

## Supporting information



Supporting Information

Supporting Information

## Data Availability

The data that support the findings of this study are available in the Supporting Information of this article.

## References

[1] M. Faraday , Philos. Trans. R. Soc. Lond. 1825, 115, 440–466.

[2] M. Solà , Front. Chem. 2013, 1, 22. 24790950 10.3389/fchem.2013.00022 PMC3982536

[3] J. E. Anthony , Chem. Rev. 2006, 106, 5028–5048. 17165682 10.1021/cr050966z

[4] B. L. Feringa , W. R. Browne , Molecular Switches, Vol. 2, Wiley‐VCH, Weinheim, Germany, 10.1002/9783527634408.

[5] A. Gürses , M. Açıkyıldız , K. Güneş , M. S. Gürses , Dyes and Pigments, Springer International Publishing, Cham 2016.

[6] E. A. William Jr. , S. A. Tucker , J. C. Fetzer , Polycycl. Aromat. Compd. 1991, 2, 75–105.

[7] H. Bronstein , C. B. Nielsen , B. C. Schroeder , I. McCulloch , Nat. Rev. Chem. 2020, 4, 66–77. 37128048 10.1038/s41570-019-0152-9

[8] T. C. Pham , V.‐N. Nguyen , Y. Choi , S. Lee , J. Yoon , Chem. Rev. 2021, 121, 13454–13619. 34582186 10.1021/acs.chemrev.1c00381

[9] J. Bauri , R. B. Choudhary , G. Mandal , J. Mater. Sci. 2021, 56, 18837–18866.

[10] B. Kippelen , J.‐L. Brédas , Energy Environ. Sci. 2009, 2, 251–261.

[11] N. Weibel , S. Grunder , M. Mayor , Org. Biomol. Chem. 2007, 5, 2343–2353. 17637951 10.1039/b703287k

[12] Y. Xu , M. von Delius , Angew. Chem. Int. Ed. 2020, 59, 559–573. 10.1002/anie.201906069 31190449

[13] B. L. Feringa , J. Org. Chem. 2007, 72, 6635–6652. 17629332 10.1021/jo070394d

[14] W. R. Browne , B. L. Feringa , Nat. Nanotechnol. 2006, 1, 25–35. 18654138 10.1038/nnano.2006.45

[15] O. Ostroverkhova , Chem. Rev. 2016, 116, 13279–13412. 27723323 10.1021/acs.chemrev.6b00127

[16] J. Crassous , M. J. Fuchter , D. E. Freedman , N. A. Kotov , J. Moon , M. C. Beard , S. Feldmann , Nat. Rev. Mater. 2023, 8, 365–371.

[17] A. Narita , X.‐Y. Wang , X. Feng , K. Müllen , Chem. Soc. Rev. 2015, 44, 6616–6643. 26186682 10.1039/c5cs00183h

[18] L. Chen , Y. Hernandez , X. Feng , K. Müllen , Angew. Chem. Int. Ed. 2012, 51, 7640–7654. 10.1002/anie.201201084 22777811

[19] S. Fujii , T. Enoki , Acc. Chem. Res. 2013, 46, 2202–2210. 24383129 10.1021/ar300120y

[20] Y. Zhu , J. Wang , Acc. Chem. Res. 2023, 56, 363–373. 36700652 10.1021/acs.accounts.2c00767

[21] M. Rickhaus , M. Mayor , M. Juríček , Chem. Soc. Rev. 2017, 46, 1643–1660. 28225107 10.1039/c6cs00623j

[22] S. H. Pun , Q. Miao , Acc. Chem. Res. 2018, 51, 1630–1642. 29974752 10.1021/acs.accounts.8b00140

[23] M. Buendía , J. M. Fernández‐García , J. Perles , S. Filippone , N. Martín , Nat. Synth. 2024, 3, 545–553.

[24] S. Ramírez‐Barroso , F. Romeo‐Gella , J. M. Fernández‐García , S. Feng , L. Martínez‐Fernández , D. García‐Fresnadillo , I. Corral , N. Martín , R. Wannemacher , Adv. Mater. 2023, 35, 2212064. 10.1002/adma.202212064 37094332

[25] C.‐N. Feng , M.‐Y. Kuo , Y.‐T. Wu , Angew. Chem. Int. Ed. 2013, 52, 7791–7794; 10.1002/anie.201303875 23794166

[26] R. W. Miller , A. K. Duncan , S. T. Schneebeli , D. L. Gray , A. C. Whalley , Chem. Eur. J. 2014, 20, 3705–3711. 24615957 10.1002/chem.201304657 PMC4371848

[27] A. Rajca , M. Miyasaka , S. Xiao , P. J. Boratyński , M. Pink , S. Rajca , J. Org. Chem. 2009, 74, 9105–9111. 19886651 10.1021/jo902030u

[28] M. Baudillon , T. Cauchy , N. Vanthuyne , N. Avarvari , F. Pop , Org. Chem. Front. 2022, 9, 4260–4270.

[29] J. Liu , J. Hong , Z. Liao , J. Tan , H. Liu , E. Dmitrieva , L. Zhou , J. Ren , X.‐Y. Cao , A. A. Popov , Y. Zou , A. Narita , Y. Hu , Angew. Chem. Int. Ed. 2024, 63, e202400172. 10.1002/anie.202400172 38345140

[30] C.‐N. Feng , W.‐C. Hsu , J.‐Y. Li , M.‐Y. Kuo , Y.‐T. Wu , Chem. Eur. J. 2016, 22, 9198–9208. 27243750 10.1002/chem.201600124

[31] B. Liu , M. Chen , X. Liu , R. Fu , Y. Zhao , Y. Duan , L. Zhang , J. Am. Chem. Soc. 2023, 145, 28137–28145. 38095317 10.1021/jacs.3c10303

[32] R. Fu , X. Chen , F. Qiu , X. Liu , J. Xia , L. Zhang , Angew. Chem. Int. Ed. 2025, 64, e202420419. 10.1002/anie.202420419 39568338

[33] Y. Matsuo , F. Chen , K. Kise , T. Tanaka , A. Osuka , Chem. Sci. 2019, 10, 11006–11012. 32110354 10.1039/c9sc05087f PMC7012041

[34] F. Chen , T. Tanaka , T. Mori , A. Osuka , Chem. Eur. J. 2018, 24, 7489–7497. 29533480 10.1002/chem.201800617

[35] B. Lousen , S. K. Pedersen , P. Bols , K. H. Hansen , M. R. Pedersen , O. Hammerich , S. Bondarchuk , B. Minaev , G. V. Baryshnikov , H. Ågren , M. Pittelkow , Chem. Eur. J. 2020, 26, 4935–4940. 32052498 10.1002/chem.201905339

[36] T. Iwanaga , T. Oki , Y. Morioka , S. Inoue , H. Sato , J. Org. Chem. 2022, 87, 14855–14860. 36219831 10.1021/acs.joc.2c01571

[37] C. Maeda , S. Nomoto , K. Akiyama , T. Tanaka , T. Ema , Chem. Eur. J. 2021, 27, 15699–15705. 34449114 10.1002/chem.202102269

[38] S. Mallick , K. Kollimalaian , P. Chetti , V. Parthasarathy , Chem. Eur. J. 2024, 30, e202302876. 37747146 10.1002/chem.202302876

[39] M. I. Khalid , M. S. H. Salem , M. Sako , M. Kondo , H. Sasai , S. Takizawa , Commun. Chem. 2022, 5, 1–7. 36697698 10.1038/s42004-022-00780-7 PMC9814689

[40] M. S. H. Salem , R. Sharma , M.d. I. Khalid , M. Sasi , R. Amasaki , Y. Imai , M. Arisawa , S. Takizawa , Electrochemistry 2023, 91, 112015–112015.

[41] A. Can , A. Facchetti , H. Usta , J. Mater. Chem. C 2022, 10, 8496–8535.

[42] A. G. Fix , D. T. Chase , M. M. Haley , in Polyarenes I (Eds: J. S. Siegel , Y.‐T. Wu ), Springer, Berlin, Heidelberg 2014, pp. 159–195.

[43] C. K. Frederickson , B. D. Rose , M. M. Haley , Acc. Chem. Res. 2017, 50, 977–987. 28207235 10.1021/acs.accounts.7b00004

[44] A. G. Fix , P. E. Deal , C. L. Vonnegut , B. D. Rose , L. N. Zakharov , M. M. Haley , Org. Lett. 2013, 15, 1362–1365. 23461389 10.1021/ol400318z

[45] R. P. Kaiser , D. Nečas , T. Cadart , R. Gyepes , I. Císařová , J. Mosinger , L. Pospíšil , M. Kotora , Angew. Chem. Int. Ed. 2019, 58, 17169–17174. 10.1002/anie.201908348 31539185

[46] T. Cadart , D. Nečas , R. P. Kaiser , L. Favereau , I. Císařová , R. Gyepes , J. Hodačová , K. Kalíková , L. Bednárová , J. Crassous , M. Kotora , Chem. Eur. J. 2021, 27, 11279–11284. 33830567 10.1002/chem.202100759

[47] Á. Martínez‐Pinel , L. Lezama , J. M. Cuerva , R. Casares , V. Blanco , C. M. Cruz , A. Millán , Org. Lett. 2024, 26, 6012–6017. 38967257 10.1021/acs.orglett.4c02128 PMC11267600

[48] T. Cadart , M. Kotora , Eur. J. Org. Chem. 2025, e202500036.

[49] E. Sidler , R. Hein , D. Doellerer , B. L. Feringa , J. Am. Chem. Soc. 2024, 146, 19168–19176. 38954739 10.1021/jacs.4c04191 PMC11258684

[50] L. Zhai , R. Shukla , R. Rathore , Org. Lett. 2009, 11, 3474–3477. 19594139 10.1021/ol901331p

[51] X. Yang , M. Hoffmann , F. Rominger , T. Kirschbaum , A. Dreuw , M. Mastalerz , Angew. Chem. Int. Ed. 2019, 58, 10650–10654. 10.1002/anie.201905666 31125478

[52] X. Yang , F. Rominger , M. Mastalerz , Angew. Chem. Int. Ed. 2019, 58, 17577–17582. 10.1002/anie.201908643 PMC6899884 31550407

[53] M. S. Little , S. G. Yeates , A. A. Alwattar , K. W. J. Heard , J. Raftery , A. C. Edwards , A. V. S. Parry , P. Quayle , Eur. J. Org. Chem. 2017, 2017, 1694–1703.

[54] Y. Zhang , S. H. Pun , Q. Miao , Chem. Rev. 2022, 122, 14554–14593. 35960873 10.1021/acs.chemrev.2c00186

[55] R. S. Jassas , E. Ullah Mughal , A. Sadiq , R. I. Alsantali , M. M. Al‐Rooqi , N. Naeem , Z. Moussa , S. A. Ahmed , RSC Adv. 2021, 11, 32158–32202. 35495486 10.1039/d1ra05910f PMC9041733

[56] D. T. Chase , B. D. Rose , S. P. McClintock , L. N. Zakharov , M. M. Haley , Angew. Chem. Int. Ed. 2011, 50, 1127–1130. 10.1002/anie.201006312 21268210

[57] T. Jousselin‐Oba , P. E. Deal , A. G. Fix , C. K. Frederickson , C. L. Vonnegut , A. Yassar , L. N. Zakharov , M. Frigoli , M. M. Haley , Chem. Asian J. 2019, 14, 1737–1744. 30548168 10.1002/asia.201801684

[58] T. Yanai , D. P. Tew , N. C. Handy , Chem. Phys. Lett. 2004, 393, 51–57.

[59] F. Weigend , R. Ahlrichs , Phys. Chem. Chem. Phys. 2005, 7, 3297–3305. 16240044 10.1039/b508541a

[60] V. Barone , M. Cossi , J. Phys. Chem. A 1998, 102, 1995–2001.

[61] B. D. Rose , L. E. Shoer , M. R. Wasielewski , M. M. Haley , Chem. Phys. Lett. 2014, 616–617, 137–141.

[62] J. L. Zafra , L. Qiu , N. Yanai , T. Mori , M. Nakano , M. P. Alvarez , J. T. L. Navarrete , C. J. Gómez‐García , M. Kertesz , K. Takimiya , J. Casado , Angew. Chem. Int. Ed. 2016, 55, 14563–14568. 10.1002/anie.201605997 27781355

[63] M. Nakano , H. Fukui , T. Minami , K. Yoneda , Y. Shigeta , R. Kishi , B. Champagne , E. Botek , T. Kubo , K. Ohta , K. Kamada , Theor. Chem. Acc. 2011, 130, 711–724.

[64] K. Yamaguchi , Chem. Phys. Lett. 1975, 33, 330–335.

[65] O. Trapp , J. Chromatogr. B 2008, 875, 42–47. 10.1016/j.jchromb.2008.07.047 18722828

[66] M. Rickhaus , L. Jundt , M. Mayor , Chimia 2016, 70, 192–192. 27052760 10.2533/chimia.2016.192

[67] S. Grimme , A. Hansen , S. Ehlert , J.‐M. Mewes , J. Chem. Phys. 2021, 154, 064103. 33588555 10.1063/5.0040021

[68] A. Stanger , J. Org. Chem. 2006, 71, 883–893. 16438497 10.1021/jo051746o

[69] A. Stanger , J. Org. Chem. 2010, 75, 2281–2288. 20196577 10.1021/jo1000753

[70] R. Gershoni‐Poranne , A. Stanger , Chem. Eur. J. 2014, 20, 5673–5688. 24677667 10.1002/chem.201304307

[71] A. D. Becke , J. Chem. Phys. 1993, 98, 5648–5652.

[72] W. J. Hehre , R. Ditchfield , J. A. Pople , J. Chem. Phys. 1972, 56, 2257–2261.

[73] R. Krishnan , J. S. Binkley , R. Seeger , J. A. Pople , J. Chem. Phys. 1980, 72, 650–654.

[74] N. Maurya , P. Jana , H. Sharma , S. Bandyopadhyay , S. Das , Chem. Commun. 2025, 61, 4808–4811. 10.1039/d4cc06643j 40029303

[75] J. L. Marshall , M. M. Haley , in Organic Redox Systems: Synthesis, Properties and Applications, John Wiley & Sons, New York 2016, pp. 311–358.

[76] G. G. Miera , S. Matsubara , H. Kono , K. Murakami , K. Itami , Chem. Sci. 2022, 13, 1848–1868. 35308842 10.1039/d1sc05586k PMC8848939

