# Supplementary material for: Indeno[2,1‐c]fluorene Quasi[8]circulenes Through Intramolecular Cyclization
Source: Angew Chem Int Ed Engl. 2025 Aug 21;64(40):e202510583. doi: 10.1002/anie.202510583 (PMC12462751; doi:10.1002/anie.202510583)
Supplement: Supplementary file 1 — Supporting Information [file ANIE-64-e202510583-s002.pdf]

Supporting Information

Indeno[2,1-*c*]fluorene Quasi[8]Circulenes through Intramolecular Cyclization

Eric Sidler,*[a] Robert Hein,[b] Charlotte N. Stindt[a] and Ben L. Feringa*[a]

*To whom correspondence should be addressed

b.l.feringa@rug.nl

e.c.sidler@rug.nl

a Stratingh Institute for Chemistry, University of Groningen, Nijenborgh 3, 9747 AG Groningen (The Netherlands)

b Organic Chemistry Institute, University of Münster, Corrensstraße 40, 48149 Münster, Germany

Table of Contents

1. General Remarks 3

2. Synthesis & NMR Spectra 4

Synthesis of *rac*-1 5

Synthesis of *rac*-2 6

Synthesis of *rac*-3 8

Synthesis of *rac*-4 11

Synthesis of *rac*-5 13

Synthesis of *rac*-6 15

Synthesis of *rac*-7 17

3. Crystal Structure 19

4. Aggregation Studies 21

5. Chiral Resolution 26

6. Spectroscopy 27

7. Enantiomerization Barrier 30

8. Electrochemistry 32

9. Computations 41

Structure Optimizations 41

Enantiomerization Barrier 42

TD-DFT 43

NICS-Calculations 45

Diradical Character 46

XYZ Coordinates 46

10. References 47

1. General Remarks

All reagents were obtained from commercial sources and used as received without further purification. Dry solvents were obtained from a MBraun solvent purification system. Progress of the reactions was determined by TLC: silica gel 60, Merck, 0.25 mm. The TLC plates were visualized with ultraviolet (UV) light (λ = 254 nm or 355 nm). High Resolution Mass Spectrometry (HRMS) measurements were performed using a Thermo Scientific Orbitrap Exploris 480. NMR spectra were recorded on a Bruker Avance Neo (^{1}H: 600 MHz, ^{13}C: 151 MHz, ^{19}F: 565 MHz). Chemical shifts (δ) are in parts per million (ppm) relative to TMS. For ^{1}H NMR spectroscopy, the splitting pattern of peaks is designated as follows: s (singlet), d (doublet), t (triplet), q (quartet), m (multiplet), dd (doublet of doublets), td (triplet of doublets), dq (quartet of doublets), and qt (quartet of triplets). Single-crystal X-ray diffraction measurements were performed on a Bruker-AXS D8 Venture diffractometer. UV/Vis absorption spectra were recorded on a Agilent Cary 8454 spectrophotometer or a JASCO V-770 in a 1 cm quartz cuvette. CD spectra were obtained on a Jasco J-715 spectropolarimeter. All electrochemical measurements were carried out in a three-electrode configuration using a PalmSens4 potentiostat, a Pt wire counter electrode, a Ag/$AgNO_3$ (10 mM in CH_3CN, 100 mM $TBAPF_6$) reference electrode and a glassy carbon (GC) disk electrode (3 mm diameter). $TBAPF_6$ was obtained from BLD and recrystallized twice from EtOH before use. Geometry optimizations and TD-DFT calculations were performed using the Orca 5.0.1. package and Orca 6.0.0. package.[1] The NICS calculations were performed with the Gaussian 16 Rev. B.01 software package.[2]

2. Synthesis & NMR Spectra

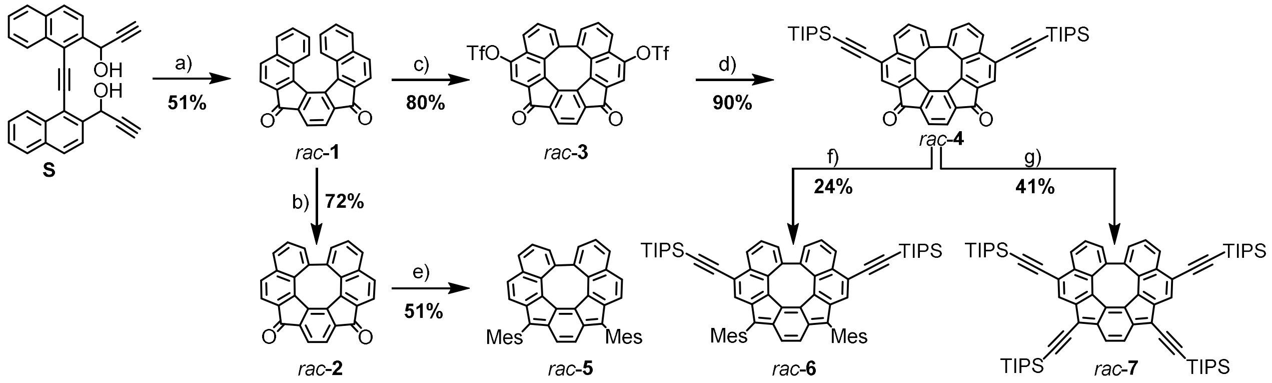

Scheme S1. Overview of the developed synthetic routes. Conditions: a) 1. $Rh(PPh_3)_3Cl$, PhMe, 130 °C, 16 h. 2. Pyridinium chlorochromate, celite, CH_2Cl_2, rt, 16 h; b) DDQ, $MeSO_3H$, CH_2Cl_2, 40 °C, 16 h; c) DDQ, TfOH, CH_2Cl_2, 40 °C, 16 h; d) (Triisopropylsilyl)acetylene, $Pd(PPh_3)_4$, CuI, THF/NEt_3 (3:1), 80 °C, 16 h; e) 1. 2-Mesitylmagnesium bromide, THF, 0 °C to rt, 1 h. 2. $SnCl_2{\cdot}2(H_2O)$, PhMe, rt, 16 h; f) 1. 2-Mesitylmagnesium bromide, THF, 0 °C to rt, 1 h. 2. $SnCl_2{\cdot}2(H_2O)$, PhMe, 45 °C, 16 h; g). (Triisopropylsilyl)acetylene, *n*BuLi, 0 °C to rt, 1 h; 2. $SnCl_2{\cdot}2(H_2O)$, PhMe, 40 °C, 16 h.

Synthesis of *rac*-1

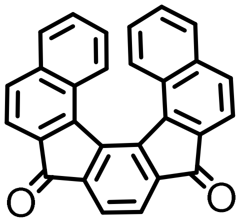

The synthesis of *rac*-**1** was slightly adapted from literature to obviate the need for a microwave and to allow access to larger amounts of compound.[3]

A Schlenk flask was charged with **S**[3] (1.00 g, 2.59 mmol, 1 eq.), Wilkinson's catalyst (72 mg, 78 µmol, 0.03 eq.) and toluene (130 mL). The reaction was stirred at 130 °C overnight before cooling the mixture to room temperature. The solvent was removed under reduced pressure before adding CH_2Cl_2 (30 mL), pyridinium chlorochromate (1.67 g, 7.76 mmol, 3 eq.) and celite (200 mg). The mixture was stirred at room temperature overnight before filtering it through a mixture of silica/celite (4:1) and eluting with CH_2Cl_2. After removing the solvent under reduced pressure, pure *rac*-**1** (503 mg, 51%) was obtained as a red solid.

The analytical data matched with the literature report.[3]

Synthesis of *rac*-2

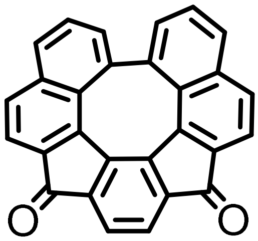

A Schlenk flask was charged with *rac*-**1** (100 mg, 0.26 mmol), DDQ (415 mg, 1.83 mmol, 7 eq.), CH_2Cl_2 (18 mL), and CH_3SO_3H (2 mL) before closing it with a stopper. The reaction mixture was stirred at 40 °C for 16 h before cooling it to room temperature. The reaction mixture was poured into a separatory funnel and diluted with CH_2Cl_2, before adding sat. aq. $NaHCO_3$. The phases were separated and the aqueous layer was extract with CH_2Cl_2. The combined organic layers were washed with brine, dried with $MgSO_4$, and filtered. The filtrate was concentrated under reduced pressure and the crude product was purified by flash column chromatography (SiO_2, CH_2Cl_2/EtOAc 1:0 to 9:1), which gave a red solid that was further purified by two consecutive trituration steps with methanol to yield pure product as an orange solid (72 mg, 72%).

^{1}H NMR (600 MHz, $CDCl_3$) δ 7.84 (dd, *J* = 8.1, 1.2 Hz, 2H), 7.82 (d, *J* = 8.2 Hz, 2H), 7.79 (d, *J* = 8.1 Hz, 2H), 7.51 (dd, *J* = 8.0, 7.1 Hz, 2H), 7.46 (s, 2H), 7.04 (dd, *J* = 7.1, 1.3 Hz, 2H); ^{13}C NMR (151 MHz, $CDCl_3$) δ 193.03, 144.41, 142.85, 141.74, 138.72, 138.43, 136.22, 134.02, 131.52, 130.49, 128.83, 128.05, 124.14, 120.58; HR-ESI-MS (+): *m/z* calcd. for $C_{28}H_{13}O_2$ [M+H]$^+$: 381.0910, found: 381.0909.

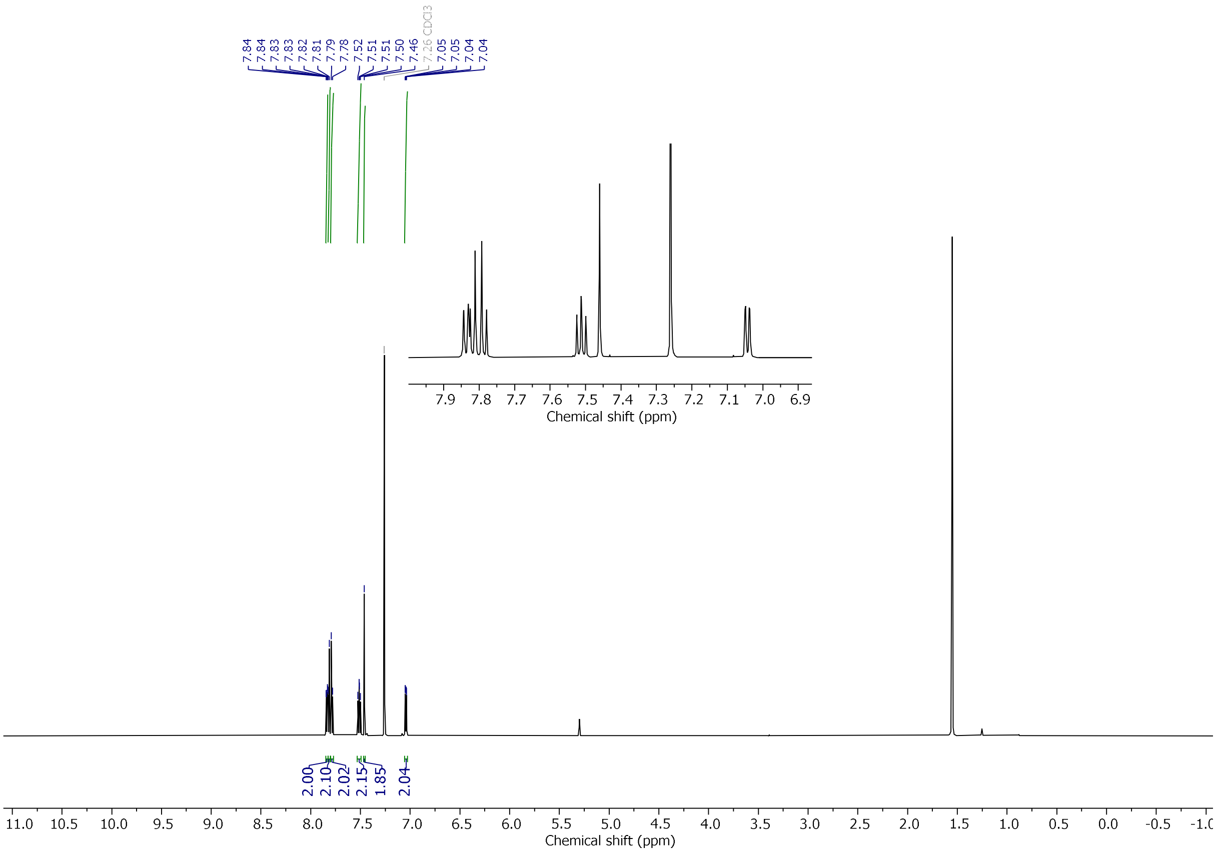

Figure S 1. ^{1}H NMR (600 MHz, $CDCl_3$, 298 K) spectrum of *rac*-**2**.

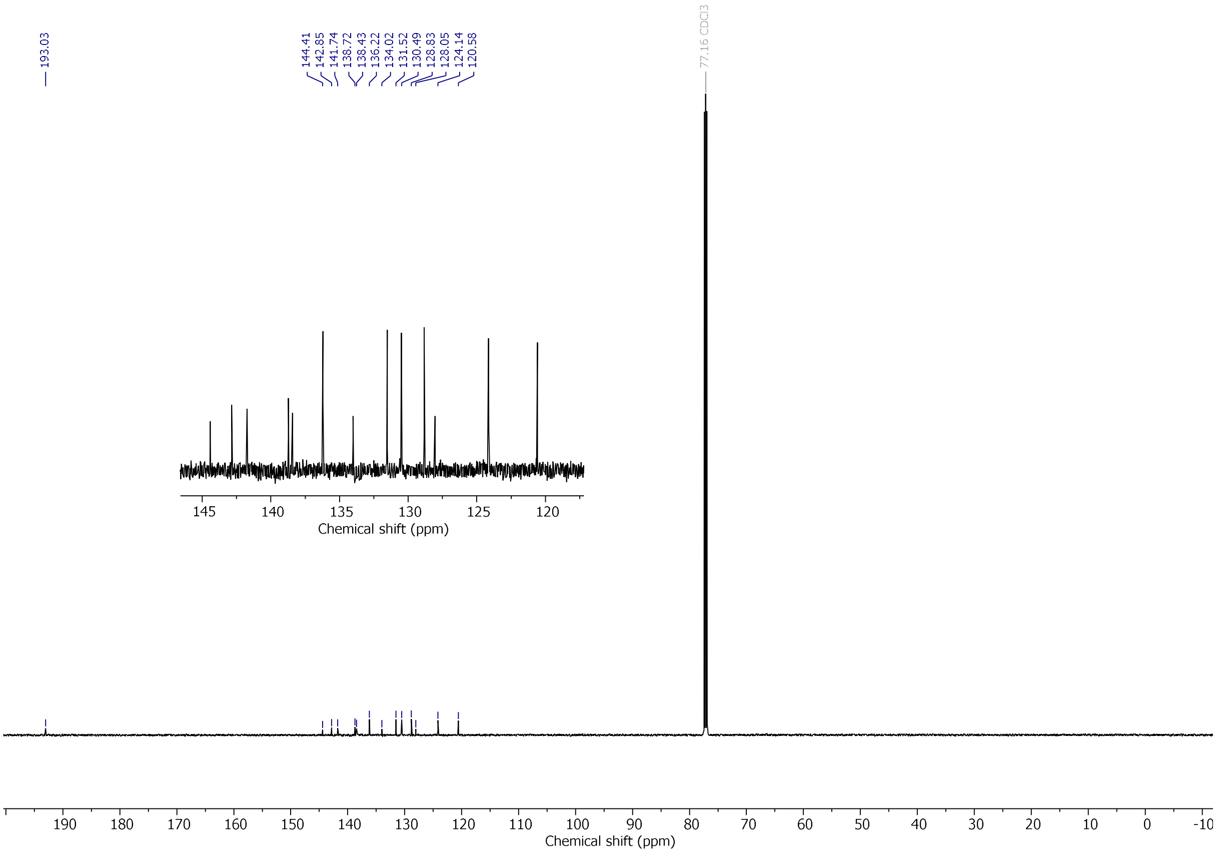

Figure S 2. ^{13}C NMR (151 MHz, $CDCl_3$, 298 K) spectrum of *rac*-**2**.

Synthesis of *rac*-3

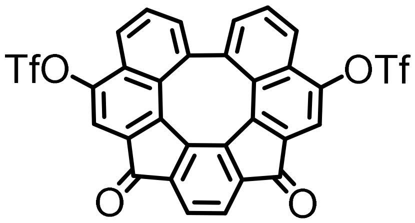

A Schlenk flask was charged with *rac*-**1** (100 mg, 0.26 mmol), DDQ (415 mg, 1.83 mmol, 7 eq.), CH_2Cl_2 (18 mL), and CF_3SO_3H (2 mL) before closing it with a stopper. The reaction mixture was stirred at 40 °C for 16 h and then cooled to room temperature. The reaction mixture was poured into a separatory funnel and diluted with CH_2Cl_2, before adding sat. aq. $NaHCO_3$. The phases were separated and the aqueous layer was extract with CH_2Cl_2. The combined organic layers were washed with brine, dried with $MgSO_4$, and filtered. The filtrate was concentrated under reduced pressure and the crude product was purified by flash column chromatography (SiO_2, CH_2Cl_2) to yield pure product as an orange solid (142 mg, 80%).

^{1}H NMR (600 MHz, $CDCl_3$) δ 8.18 (dd, *J* = 8.4, 1.2 Hz, 2H), 7.79 (s, 2H), 7.73 (dd, *J* = 8.4, 7.2 Hz, 2H), 7.59 (s, 2H), 7.16 (dd, *J* = 7.2, 1.2 Hz, 2H); ^{13}C NMR (151 MHz, $CDCl_3$) δ 190.74, 146.68, 143.62, 142.19, 141.58, 137.83, 137.72, 133.74, 131.24, 130.70, 129.21, 125.25, 123.61, 112.95; CF_3 carbon signal is not observed; ^{19}F NMR (565 MHz, $CDCl_3$) δ -72.96; HR-ESI-MS (+): *m/z* calcd. for $C_{30}H_{11}F_6O_8S_2$ $[M+H]^+$: 676.9794, found: 676.9802.

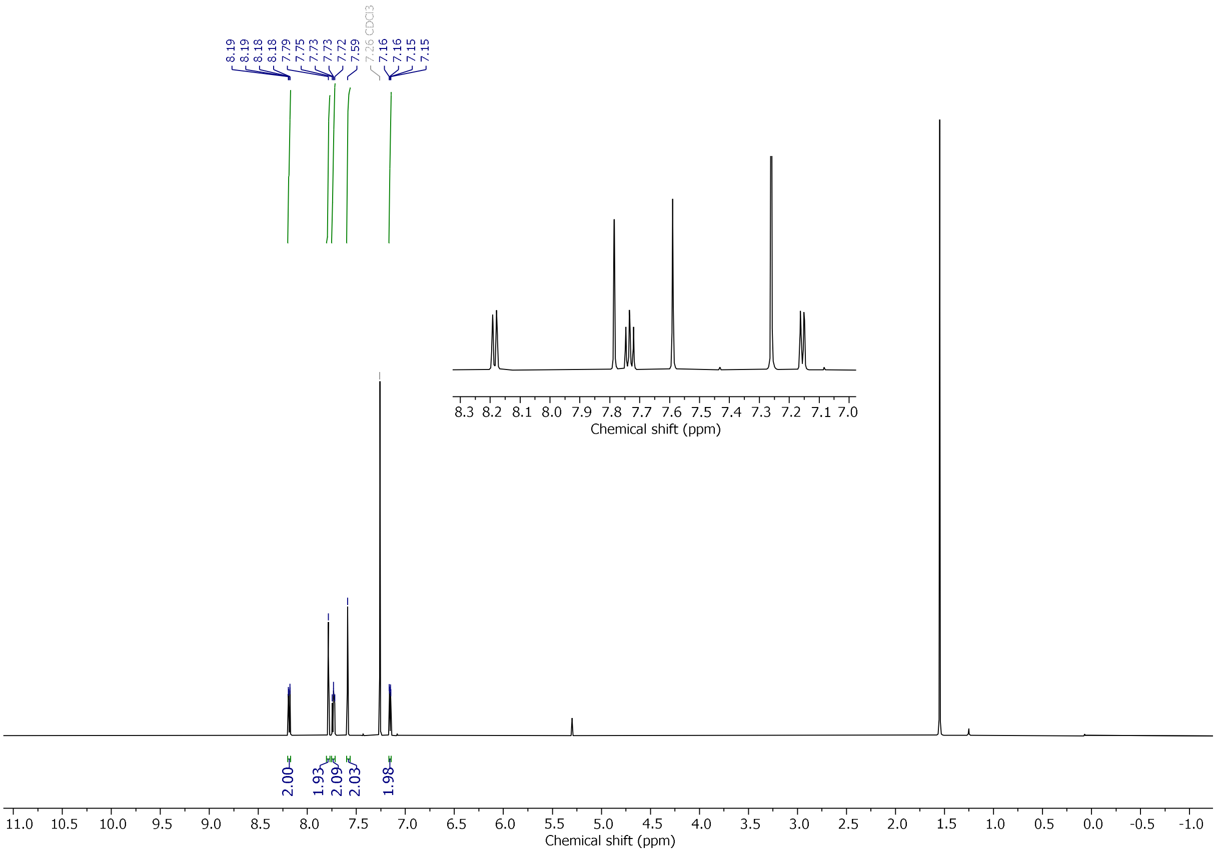

Figure S 3. ^{1}H NMR (600 MHz, $CDCl_3$, 298 K) spectrum of *rac*-**3**.

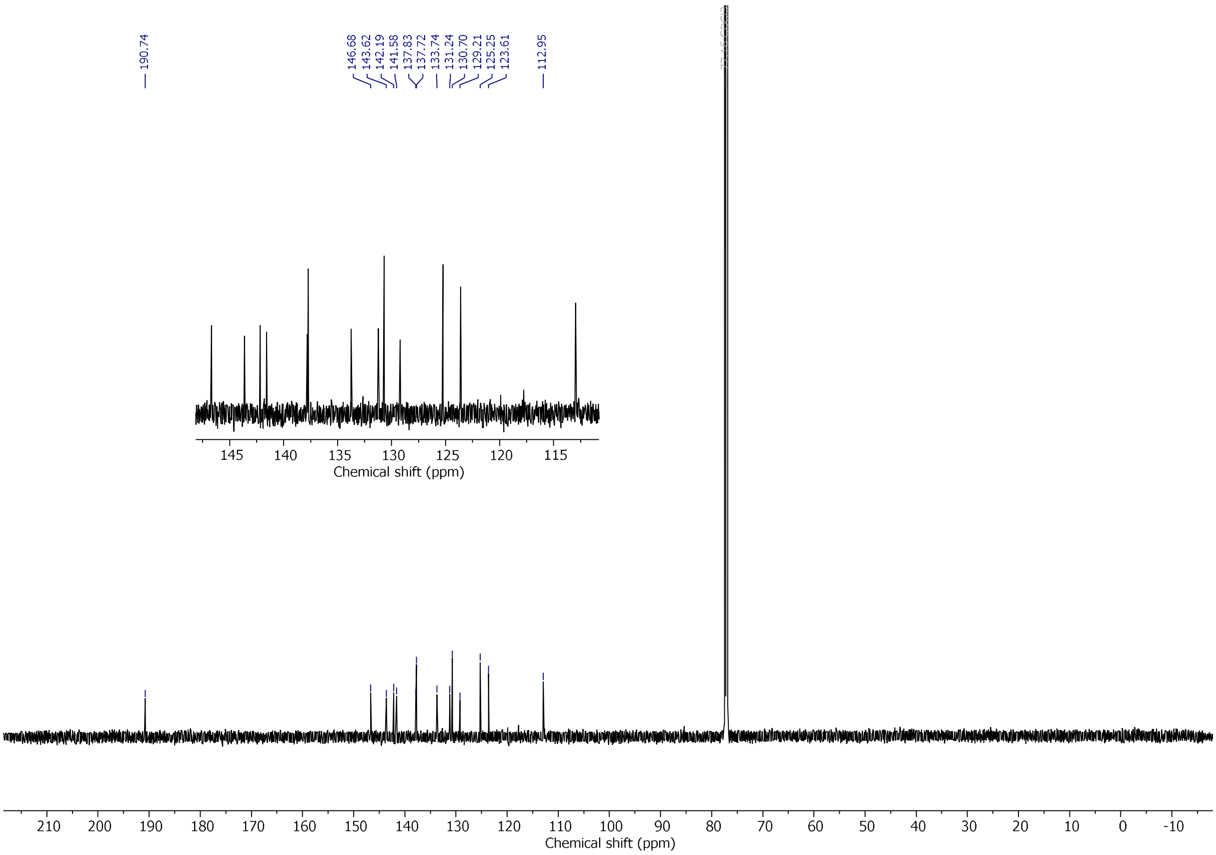

Figure S 4. ^{13}C NMR (151 MHz, $CDCl_3$, 298 K) spectrum of *rac*-**3**.

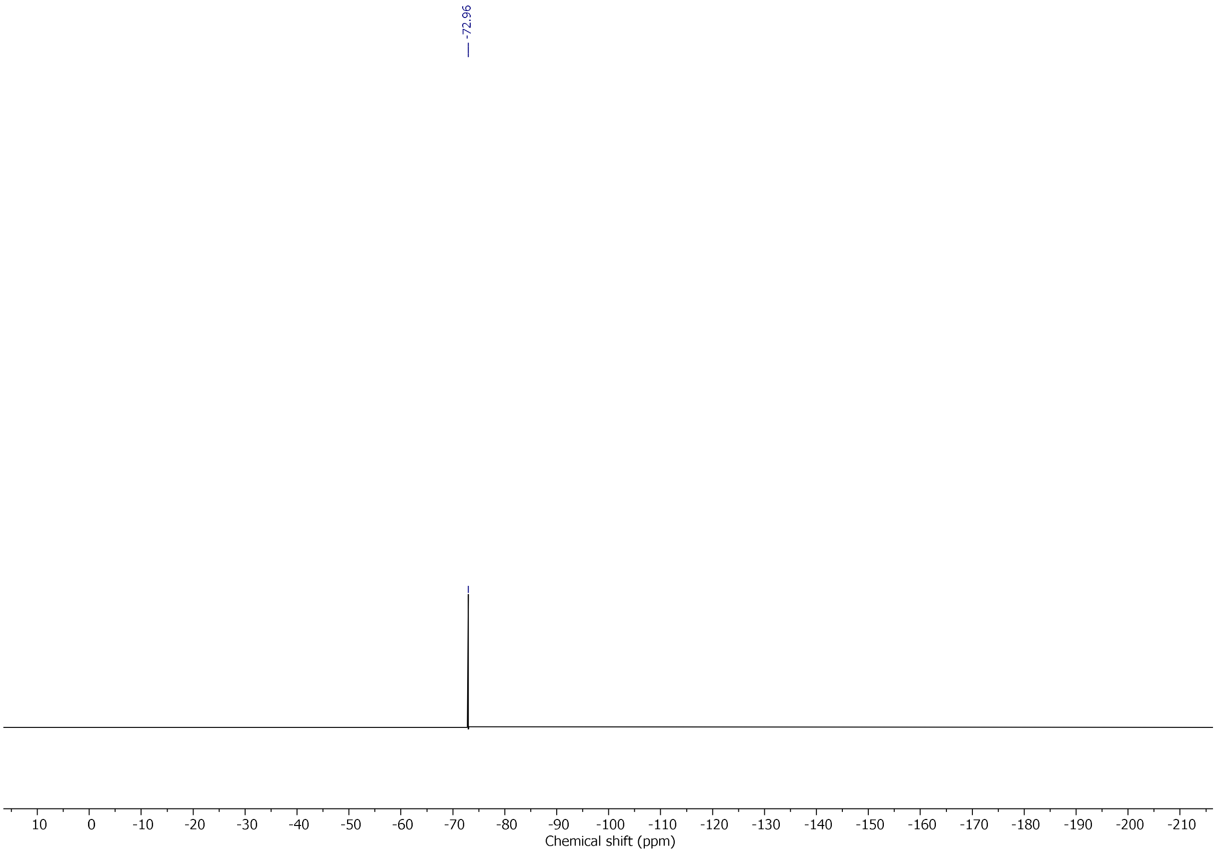

Figure S 5. ^{19}F NMR (565 MHz, $CDCl_3$, 298 K) spectrum of *rac*-**3**.

Synthesis of *rac*-4

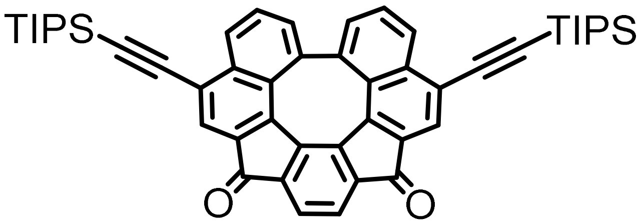

A Schlenk flask was charged with *rac*-**3** (82 mg, 0.12 mmol) before adding THF (8.1 mL) and NEt_3 (2.7 mL). The suspension was degassed with nitrogen for 10 min before adding a spatula tip of $Pd(PPh_3)_4$ and CuI, followed by further degassing for 5 min. Tri(isopropylsilyl)acetylene (110 µL, 0.485 mmol, 4 eq.) was added and the mixture was further degassed for another 2 min before closing the flask with a stopper and putting the flask into a preheated oil bath at 80 °C overnight. The reaction mixture was cooled to room temperature and concentrated under reduced pressure. The crude product was purified by flash column chromatography (SiO_2, pentane/CH_2Cl_2 1:0 to 1:1) to isolate pure product as a red solid (81 mg, 90%).

^1H NMR (600 MHz, $CDCl_3$) δ 8.48 (dd, *J* = 8.3, 1.3 Hz, 2H), 7.98 (s, 2H), 7.56 (dd, *J* = 8.3, 7.1 Hz, 2H), 7.46 (s, 2H), 6.95 (dd, *J* = 7.2, 1.3 Hz, 2H), 1.24 – 1.19 (m, 42H); ^{13}C NMR (151 MHz, $CDCl_3$) δ 192.17, 143.88, 142.91, 141.74, 138.39, 138.31, 136.46, 133.11, 129.61, 128.63, 128.09, 125.49, 124.39, 124.16, 104.28, 99.95, 18.92, 11.52; HR-ESI-MS (+): *m/z* calcd. for $C_{50}H_{53}O_2Si_2$ $[M+H]^+$: 741.3579, found: 741.3584.

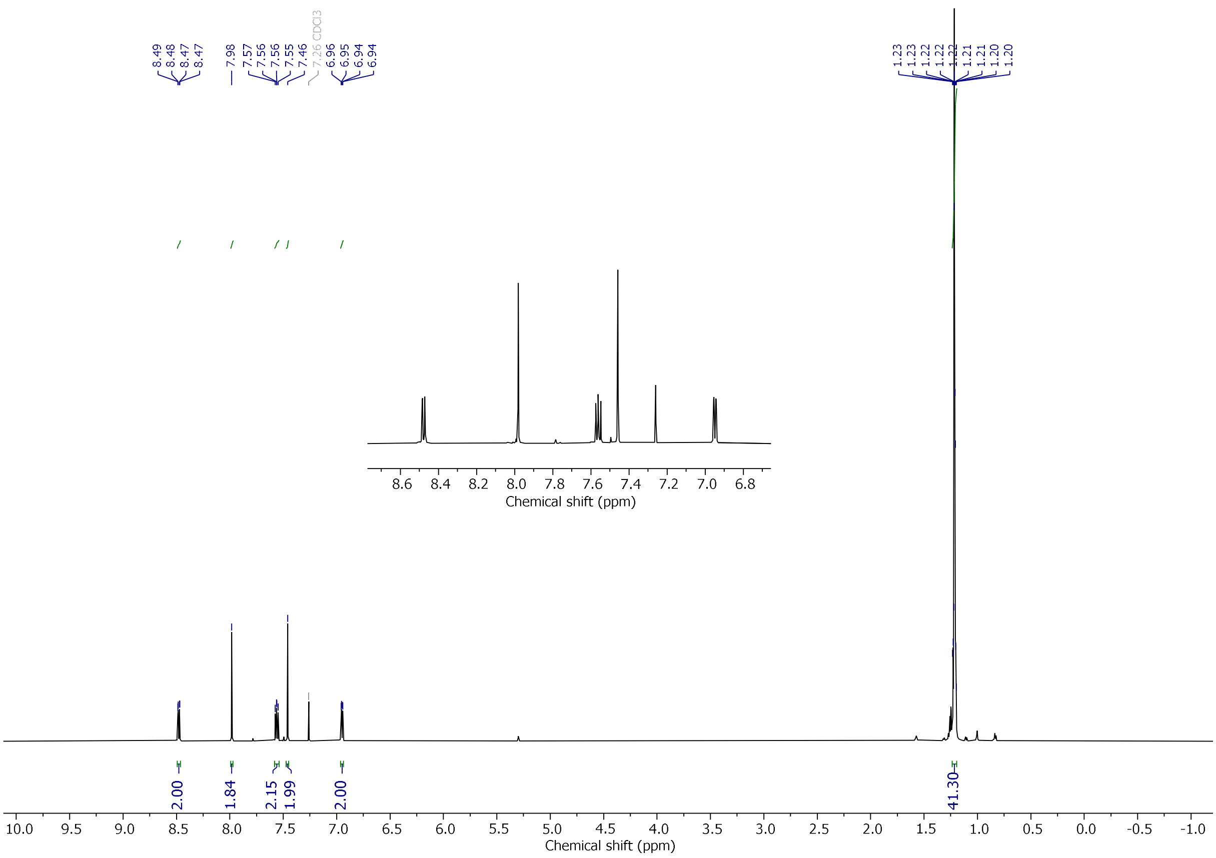

Figure S 6. ^{1}H NMR (600 MHz, $CDCl_3$, 298 K) spectrum of *rac*-**4**.

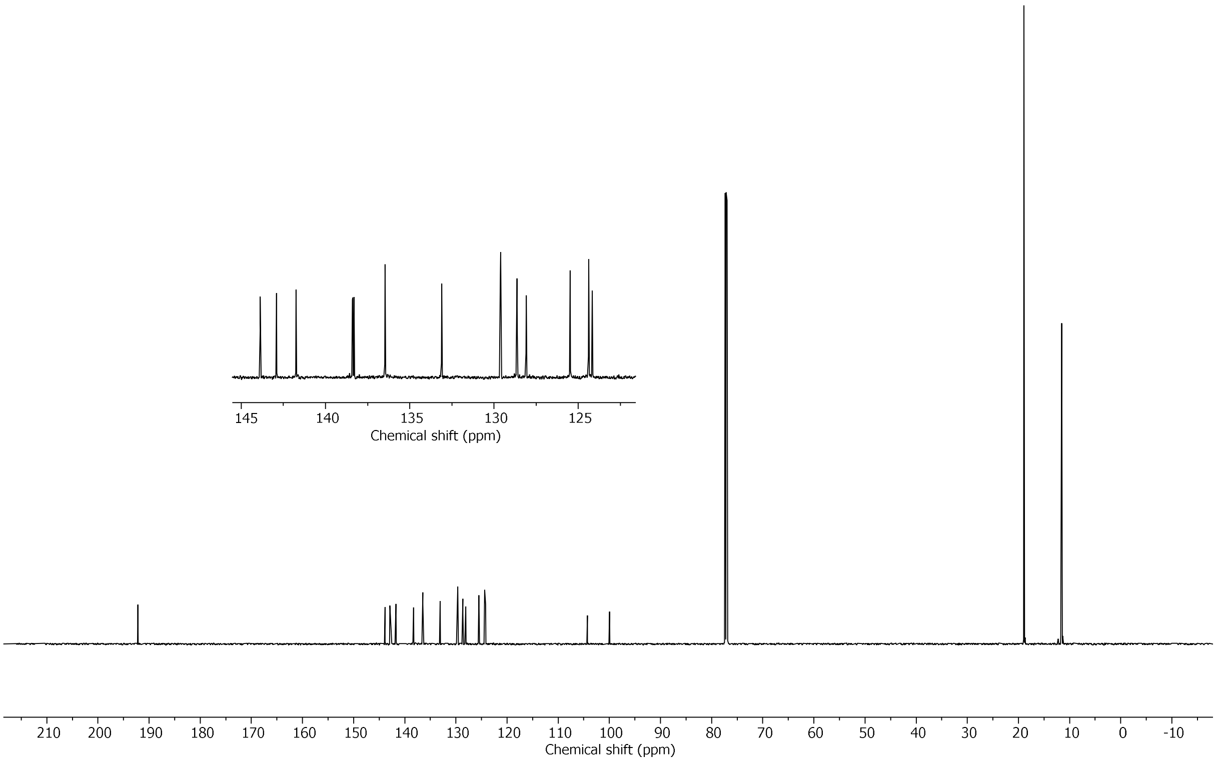

Figure S 7. ^{13}C NMR (151 MHz, $CDCl_3$, 298 K) spectrum of *rac*-**4**.

Synthesis of *rac*-5

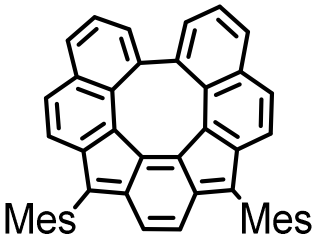

A flame dried round bottom flask was charged with *rac*-**2** (28 mg, 74 µmol, 1 eq.) and dry THF (10 mL), and the mixture was cooled to 0 °C. After dropwise addition of an excess of 2-mesitylmagnesium bromide (1 M in THF, 0.59 mL, 8 eq.), the cooling bath was removed and the reaction mixture was stirred at room temperature for 1 h. The mixture was then quenched with sat. aq. NH_4Cl and diluted with CH_2Cl_2. After phase separation, the aqueous phase was extracted with CH_2Cl_2 and the combined organic layers were dried with $MgSO_4$, filtered and evaporated under reduced pressure. The resulting brown crude oil was mixed with pentane to precipitate a beige solid that was filtered and transferred to a new round bottom flask. After the addition of toluene (10 mL), the reaction mixture was degassed with nitrogen before adding $SnCl_2 \cdot 2(H_2O)$ (66 mg, 295 µmol, 4 eq.). The resulting suspension was stirred at room temperature overnight before filtering the reaction mixture over celite and eluting with additional toluene. The solvent was evaporated under reduced pressure and the purple crude was purified by precipitation from a CH_2Cl_2/MeOH mixture to yield pure product (22 mg, 51%) as a purple solid.

^{1}H NMR (600 MHz, CD_2Cl_2) δ 7.77 (dd, *J* = 8.0, 1.4 Hz, 2H), 7.62 (d, *J* = 8.2 Hz, 2H), 7.26 (dd, *J* = 7.2, 1.4 Hz, 2H), 7.15 (dd, *J* = 7.9, 7.2 Hz, 2H), 7.01 (s, 2H), 6.99 (s, 2H), 6.78 (d, *J* = 8.2 Hz, 2H), 5.97 (s, 2H), 2.35 (s, 6H), 2.30 (s, 6H), 2.03 (s, 6H); ^{13}C NMR (151 MHz, CD_2Cl_2) δ 146.84, 146.11, 140.42, 137.75, 137.66, 137.58, 137.23, 136.57, 135.29, 132.26, 131.32, 130.17, 129.81 (d, *J* = 2.9 Hz), 129.65, 129.43, 128.14, 128.09, 121.39, 120.24, 119.82, 20.87, 20.03, 19.84; HR-ESI-MS (+): *m/z* calcd. for $C_{46}H_{34}$ $[M]^+$: 586.2655, found: 586.2651.

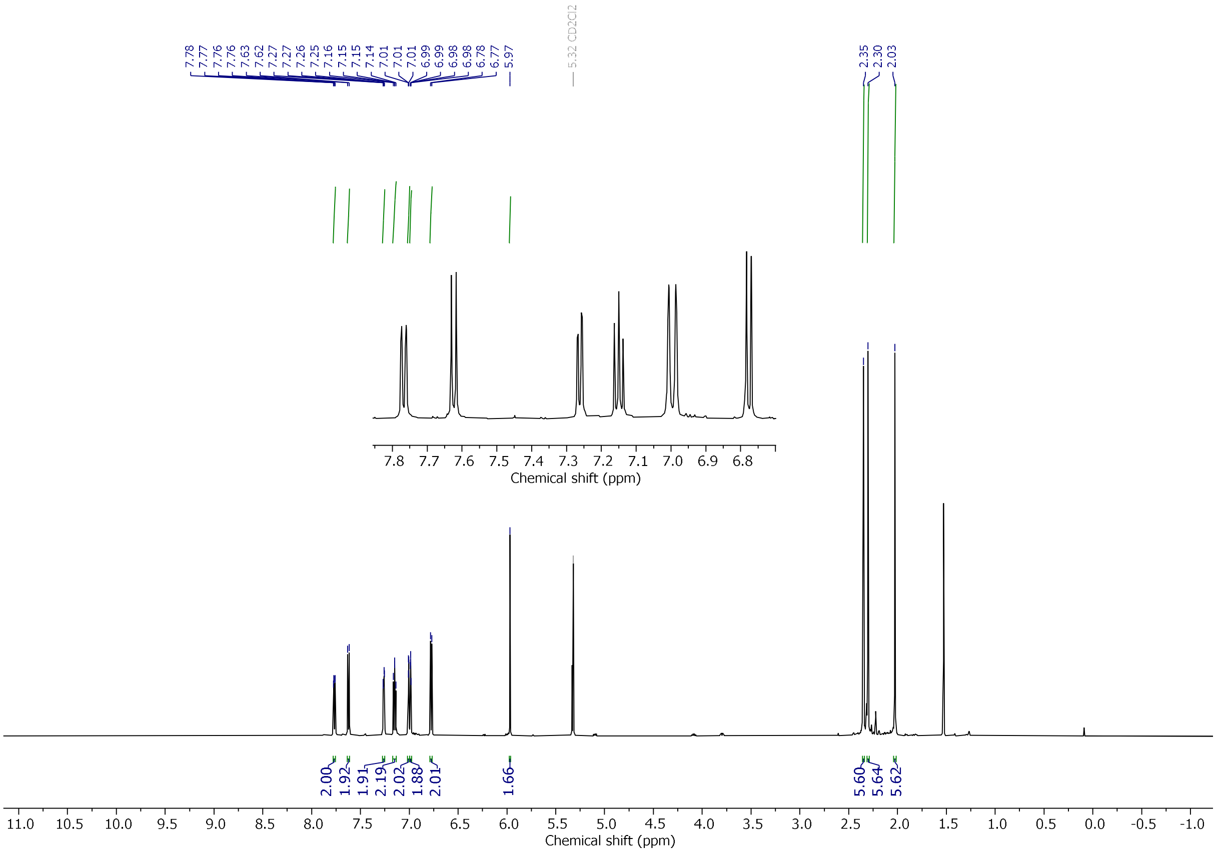

Figure S 8. ^{1}H NMR (600 MHz, CD_2Cl_2, 298 K) spectrum of *rac*-**5**.

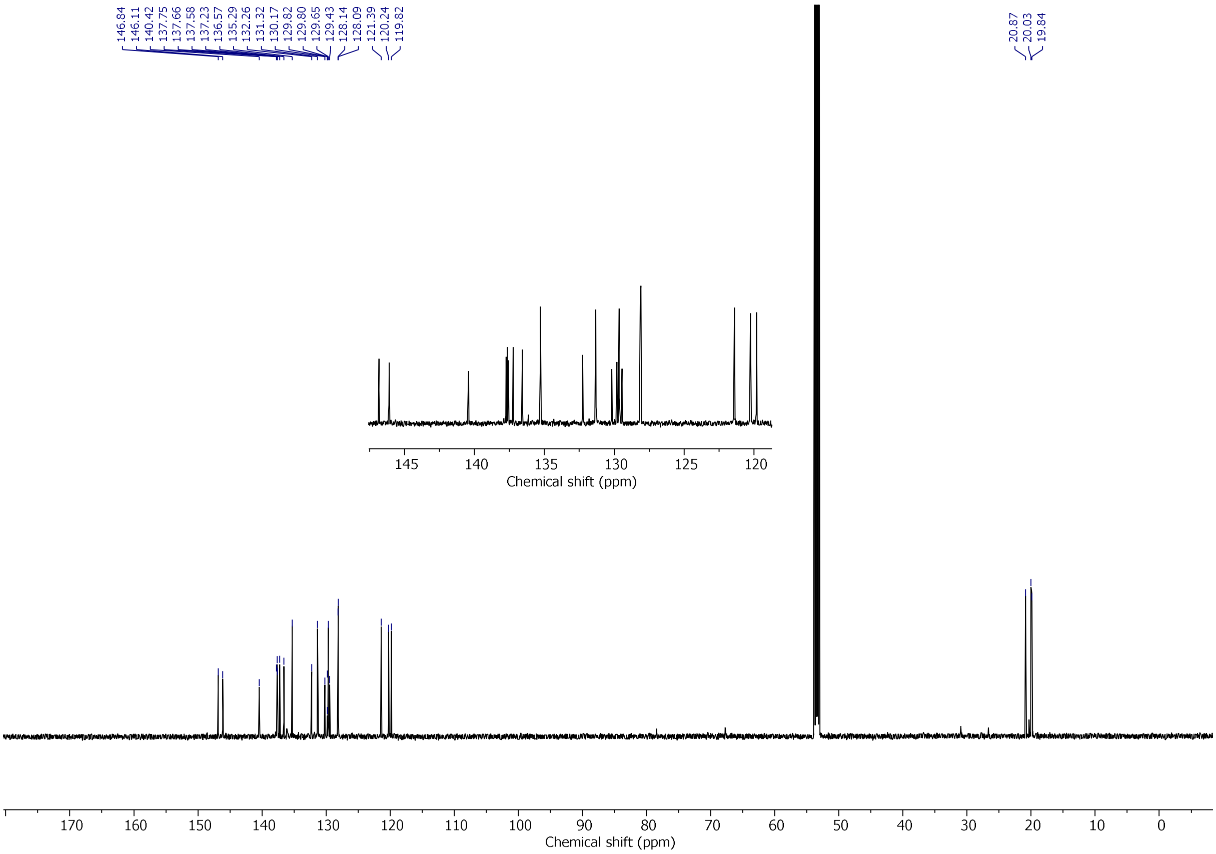

Figure S 9. ^{13}C NMR (151 MHz, CD_2Cl_2, 298 K) spectrum of *rac*-**5**.

Synthesis of *rac*-6

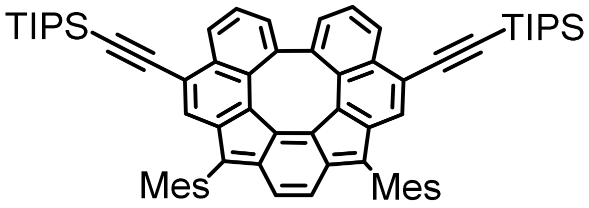

A flame dried round bottom flask was charged with *rac*-**2** (30 mg, 40 µmol, 1 eq.) and dry THF (10 mL), and the mixture was cooled to 0 °C. After dropwise addition of an excess of 2-mesitylmagnesium bromide (1 M in THF, 0.32 mL, 320 µmol, 8 eq.), the cooling bath was removed and the reaction mixture was stirred at room temperature for 1 h. The mixture was then quenched with sat. aq. NH_4Cl and diluted with CH_2Cl_2. After phase separation, the aqueous phase was extracted with CH_2Cl_2 and the combined organic layers were dried with $MgSO_4$, filtered and evaporated under reduced pressure. The resulting crude oil was dissolved in toluene (10 mL) and the solution was degassed with nitrogen before adding $SnCl_2{\cdot}2(H_2O)$ (37 mg, 162 µmol, 4 eq.). The resulting suspension was stirred at 45 °C overnight before filtering the reaction mixture over celite and eluting with additional toluene. The solvent was evaporated under reduced pressure and the purple crude was purified by flash column chromatography (SiO_2, pentane/CH_2Cl_2 1:0 to 9:1) to yield pure product (9 mg, 24%) as a purple solid.

^{1}H NMR (600 MHz, CD_2Cl_2) δ 8.47 – 8.43 (m, 2H), 7.23 – 7.19 (m, 4H), 7.01 (s, 2H), 7.00 (s, 2H), 6.97 (s, 2), 5.94 (s, 2H), 2.36 (s, 63H), 2.30 (s, 6H), 2.03 (s, 6H), 1.20 – 1.16 (m, 42H); ^{13}C NMR (151 MHz, CD_2Cl_2) δ 146.37, 146.19, 141.11, 138.81, 138.29, 138.26, 137.69, 137.00, 136.15, 132.72, 131.12, 130.16, 129.73, 128.66, 128.58, 128.05, 125.08, 123.75, 122.47, 120.52, 105.90, 98.78, 21.30, 20.49, 20.29, 18.93, 11.84; HR-ESI-MS (+): *m/z* calcd. for $C_{68}H_{75}Si_2$ $[M+H]^+$: 947.5402, found: 947.5403.

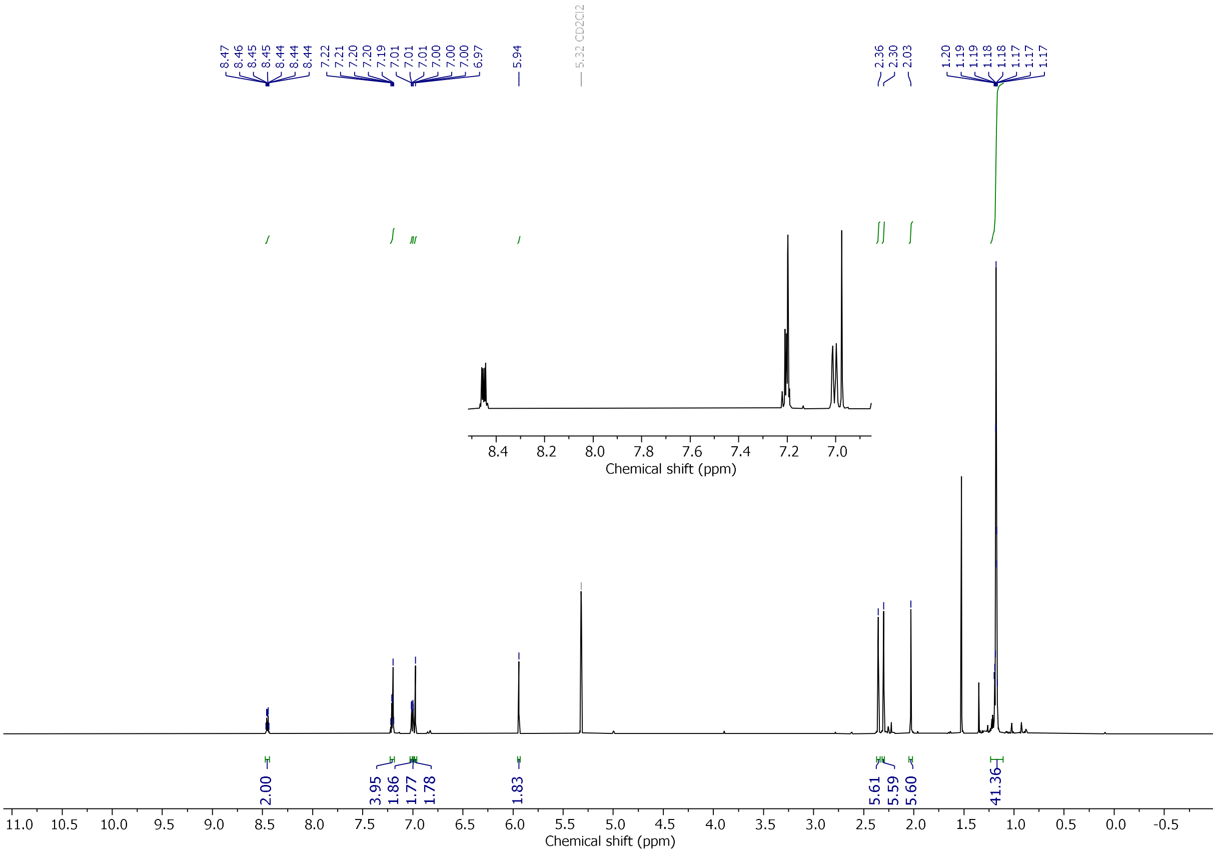

Figure S 10. ^{1}H NMR (600 MHz, CD_2Cl_2, 298 K) spectrum of *rac*-**6**.

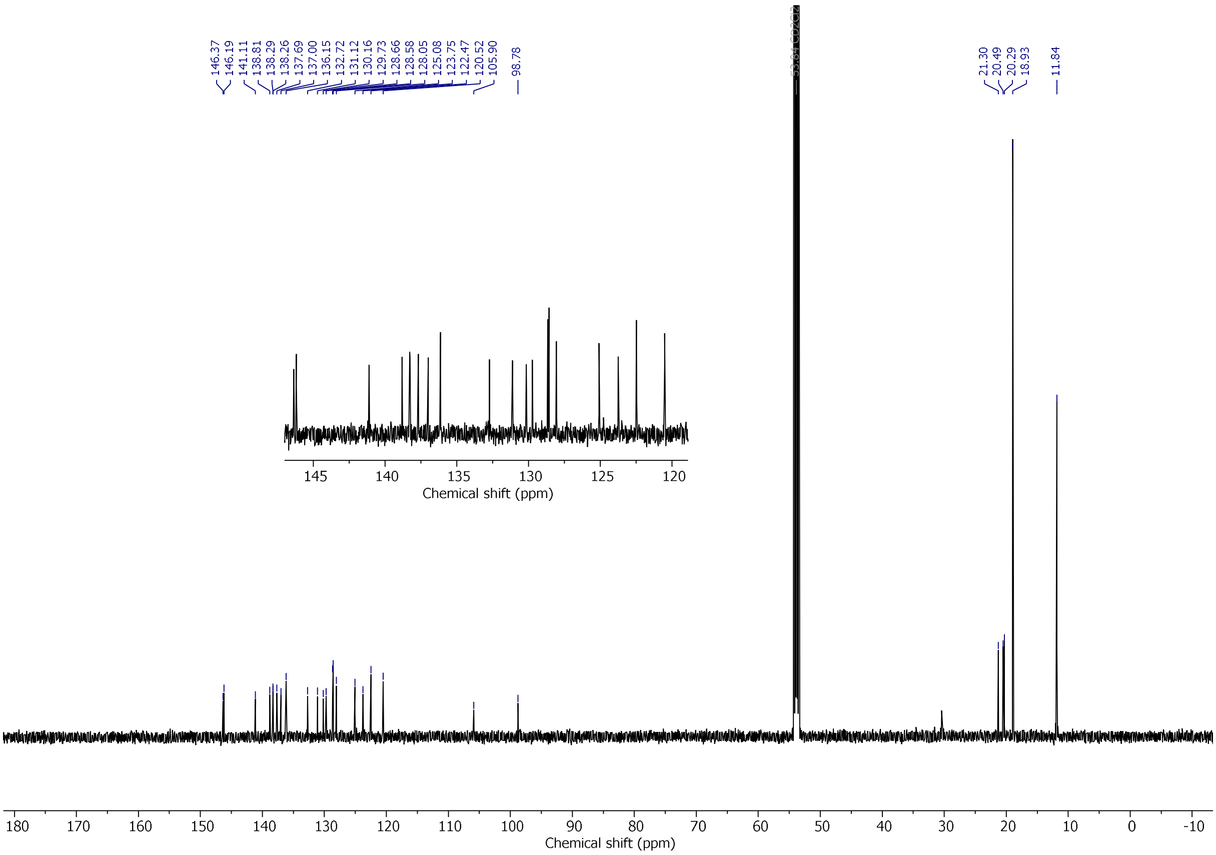

Figure S 11. ^{13}C NMR (151 MHz, CD_2Cl_2, 298 K) spectrum of *rac*-**6**.

Synthesis of *rac*-7

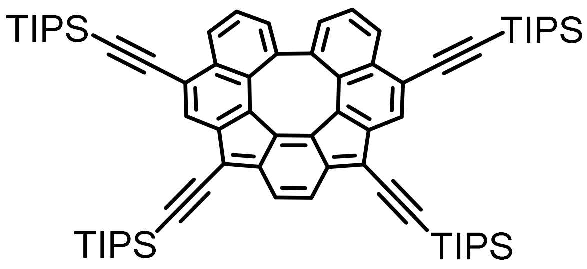

A flame dried round bottom flask was charged with dry THF (2.5 mL) and (triisopropylsilyl)acetylene (37 µL, 162 µmol, 4 eq.) before cooling the solution to 0 °C. After the dropwise addition of *n*-butyllithium (2.5 M in THF, 65 µL, 162 µmol, 4 eq.), the mixture was stirred at 0 °C for 30 min. In the meantime, another flame dried round bottom flask was charged with *rac*-**4** (30 mg, 40 µmol, 1 eq.), dry THF (5 mL) and the resulting solution was cooled to 0 °C. The previously made lithium acetylide solution was added to the solution of *rac*-**4** dropwise at 0 °C and the resulting reaction mixture was stirred at 0 °C for 30 min and then at room temperature for additional 30 min. The bright yellow solution was quenched with sat. aq. NH_4Cl and diluted with CH_2Cl_2. After phase separation, the aqueous phase was extracted with CH_2Cl_2 and the combined organic layers were dried with $MgSO_4$, filtered and evaporated under reduced pressure. The resulting crude was dissolved in toluene (10 mL) and the solution was degassed with nitrogen before adding $SnCl_2·2(H_2O)$ (36 mg, 162 µmol, 4 eq.). The resulting suspension was stirred at 40 °C overnight before filtering the reaction mixture over celite and eluting with additional toluene. The solvent was evaporated under reduced pressure and the green solid crude was purified by flash column chromatography (SiO_2, pentane/CH_2Cl_2 9:1) to yield pure product (18 mg, 41%) as a green solid.

^{1}H NMR (600 MHz, CD_2Cl_2) δ 8.42 (d, *J* = 7.7 Hz, 2H), 7.64 (s, 2H), 7.25 – 7.20 (m, 2H), 7.15 (d, *J* = 7.0 Hz, 2H), 6.68 (s, 2H), 1.26 – 1.17 (m, 84H); ^{13}C NMR (151 MHz, CD_2Cl_2) δ 144.62, 144.40, 141.33, 138.46, 136.49, 132.98, 130.69, 129.75, 128.23, 127.92, 126.02, 124.30, 123.30, 121.25, 111.81, 105.89, 101.73, 99.28, 18.95, 18.93, 11.83, 11.76; HR-ESI-MS (+): *m/z* calcd. for $C_{72}H_{95}Si_4$ $[M+H]^+$: 1071.6505, found: 1071.6509.

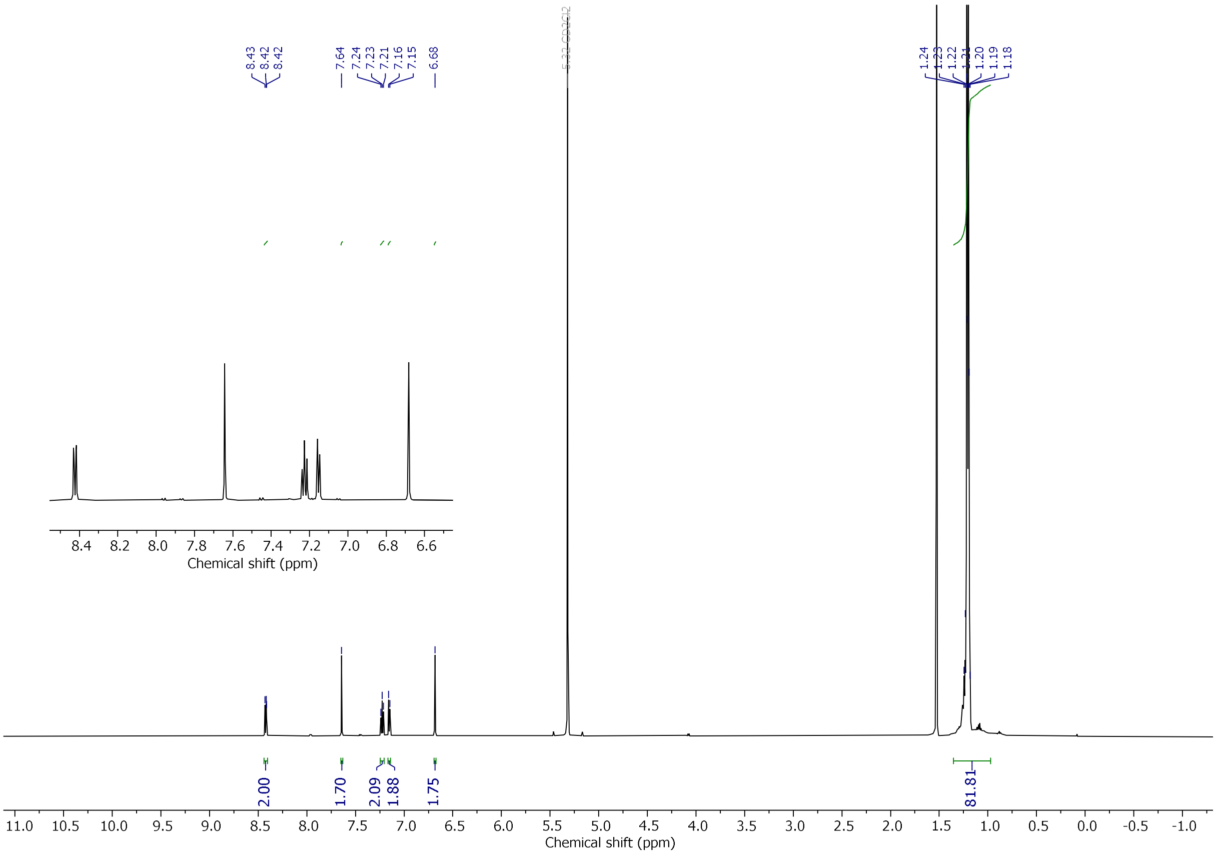

Figure S 12. ^{1}H NMR (600 MHz, CD_2Cl_2, 298 K) spectrum of *rac*-**7**.

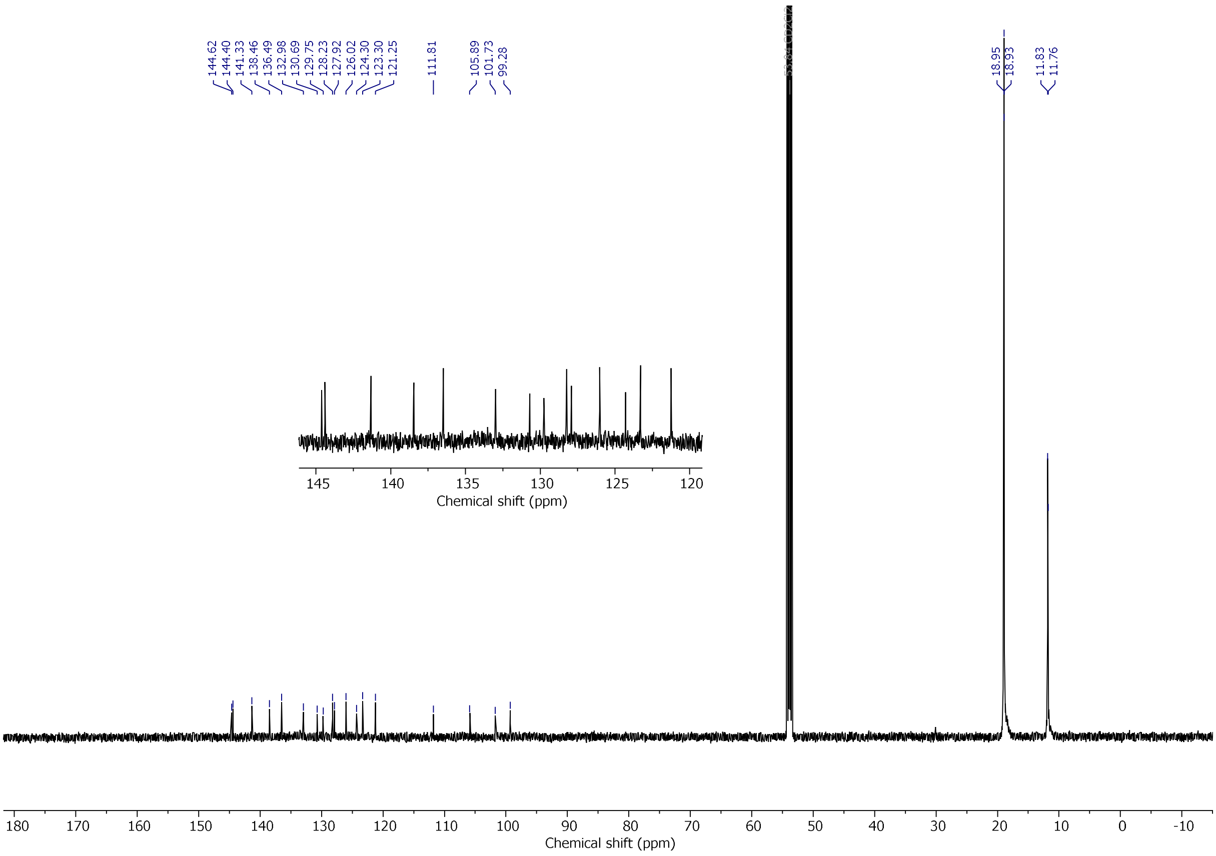

Figure S 13. ^{13}C NMR (151 MHz, CD_2Cl_2, 298 K) spectrum of *rac*-**7**.

3. Crystal Structure

Single crystals suitable for X-Ray diffraction analysis were obtained by diffusion of pentane vapors into a solution of *rac*-**3** in CH_2Cl_2 at room temperature. A single crystal was mounted on a cryoloop and cooled to 100 K in a nitrogen stream for analsysis using a Bruker-AXS D8 Venture diffractometer, with CuKα radiation (λ = 1.54178 Å). Data acquisition and processing were carried out with the Bruker APEX4 software suite, and a multi-scan absorption corrections was performed using SADABS (SADABS-2016/2).[4] The structure was solved with SHELXT[5] and refined using SHELXL[6] within the OLEX2 software package.[7] Hydrogen atoms were added based on geometrical considerations and refined using a riding model. No A- or B level alerts were raised by CheckCIF for the fully refined structure. The crystallographic data supporting this paper are available under Deposition Number 2450296 and can be accessed for free via the Cambridge Crystallographic Data Centre at https://www.ccdc.cam.ac.uk/structures/.

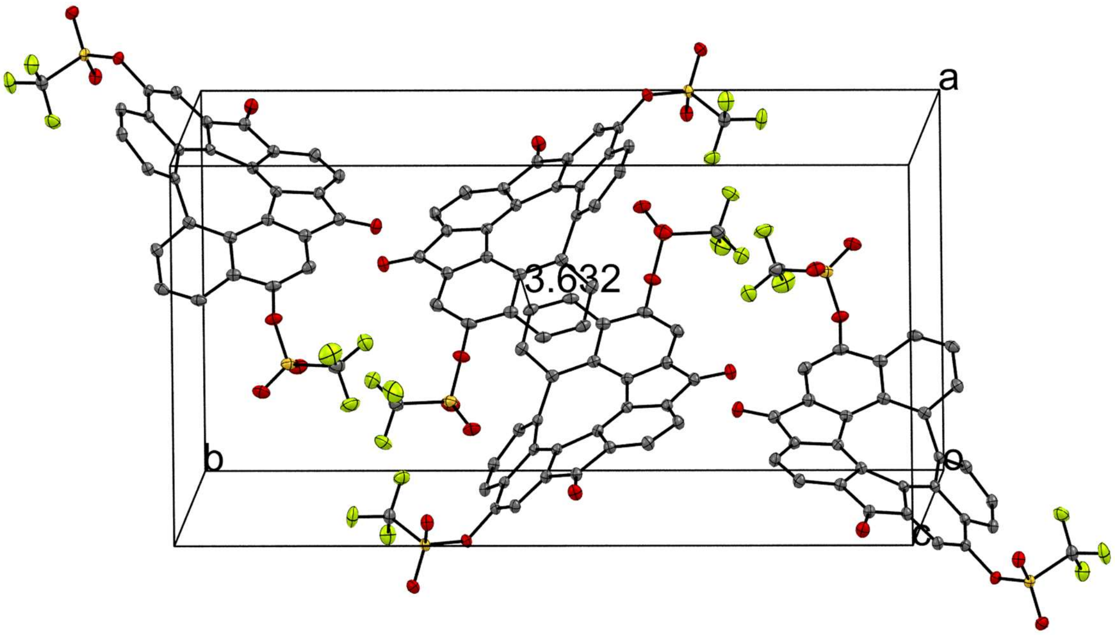

Figure S 14. ORTEP plot (50% probability level) of the crystal packing of *rac*-**3**. Hydrogen atoms are omitted for clarity.

Table S 1. Crystallographic data for *rac*-**3**.

Empirical formula	$C_{30}H_{10}F_6O_8S_2$
Formula weight	676.50
Temperature/K	100
Crystal system	monoclinic
Space group	$P2_1/c$
a/Å	12.3538(3)
b/Å	24.1064(6)
c/Å	8.7667(2)
α/°	90
β/°	101.4470(10)
γ/°	90
Volume/Å^3	2558.84(11)
Z	4
ρ_{calc}g/cm^3	1.756
μ/mm^{-1}	2.824
F(000)	1360.0
Crystal size/mm^3	0.23 × 0.138 × 0.04
Radiation	CuKα (λ = 1.54178)
2Θ range for data collection/°	7.3 to 149.076
Index ranges	-13 ≤ h ≤ 15, -30 ≤ k ≤ 30, -10 ≤ l ≤ 10
Reflections collected	97465
Independent reflections	5222 [R_{int} = 0.0766, R_{sigma} = 0.0323]
Data/restraints/parameters	5222/0/415
Goodness-of-fit on F^2	1.052
Final R indexes [I>=2σ (I)]	R_1 = 0.0370, wR_2 = 0.0975
Final R indexes [all data]	R_1 = 0.0383, wR_2 = 0.0986
Largest diff. peak/hole / e Å^{-3}	0.41/-0.61

4. Aggregation Studies

The aggregation of *rac*-**7** was investigated by Diffusion-Ordered Spectroscopy (DOSY) NMR using a concentrated sample in CD_2Cl_2. The broad peaks were shown to have a smaller but well-defined diffusion coefficient compared to the sharp monomeric peaks. Using the Stokes-Einstein-Sutherland equation

$$D = \frac{k_B T}{6\pi\eta r}$$

where D is the diffusion coefficient, η is the dynamic viscosity of the solvent and r is the hydrodynamic of the diffusing sphere, a twofold hydrodynamic radius (7.31 Å) compared to the monomeric radius (3.17 Å) was calculated. This suggests the exclusive formation of a dimeric species in the aggregate state.

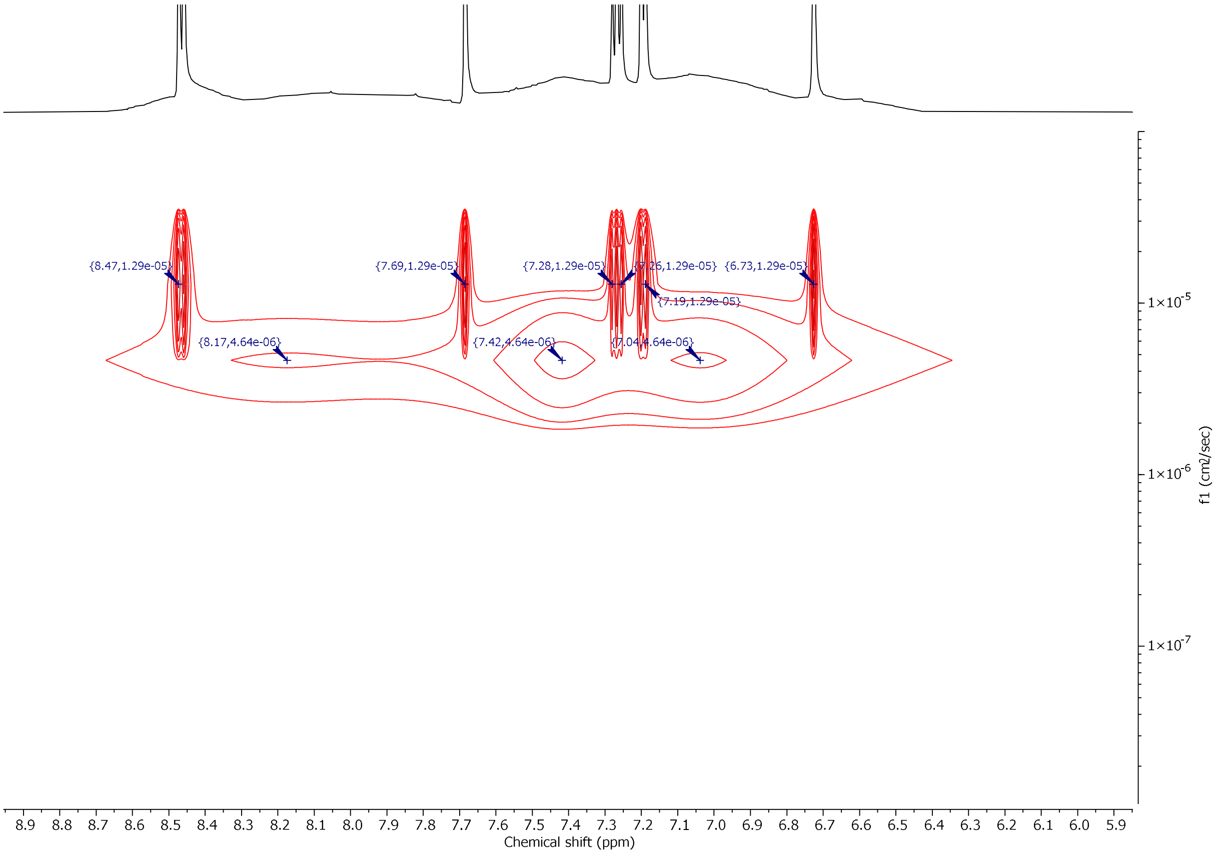

Figure S 15. ^{1}H DOSY NMR (600 MHz, CD_2Cl_2, 298 K) spectrum of *rac*-**7**.

The aggregation was then further analyzed by UV-Vis spectroscopy in THF/water mixtures, wherein with an increasing fractional volume of water a significant blueshift of the spectrum concurrent with a color change of the solution from blue to yellow was observed. At higher fractional volumes of water (>90%), precipitation occurred.

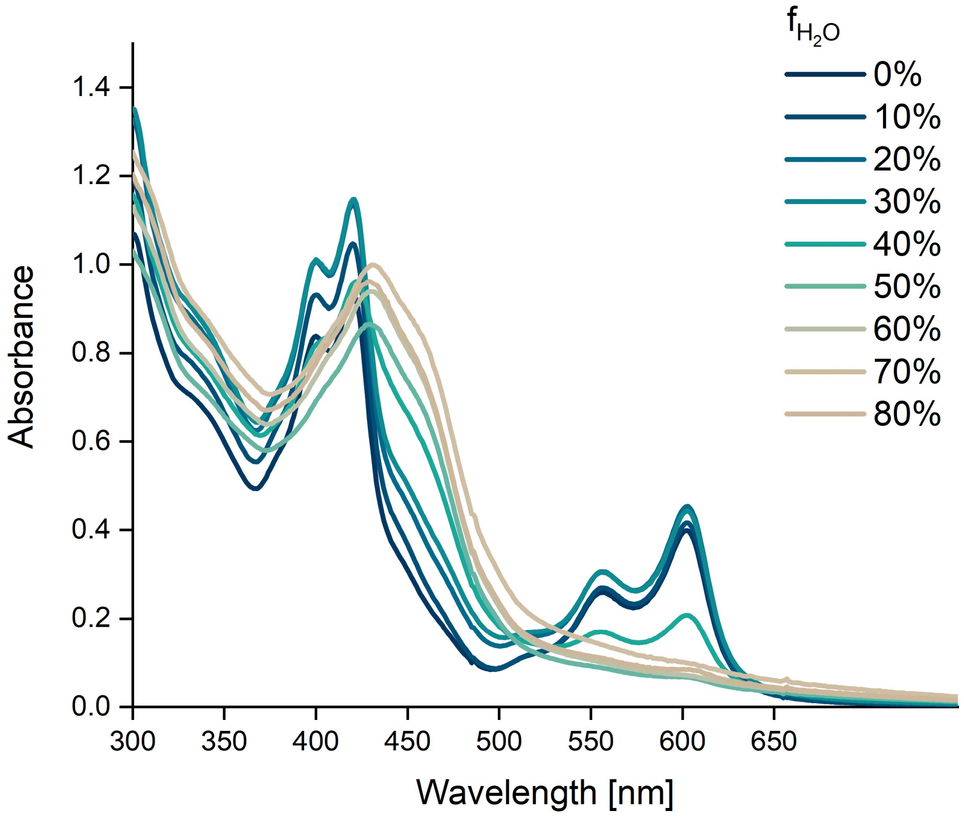

Figure S 16. UV-Vis spectra of *rac*-**7** in varying THF/water mixtures (c ~ 10^{-5} M).

The dimerization constant K_{dim} was determined by measuring the UV-Vis spectrum of *rac*-**7** at varying concentrations and plotting the absorbance at 600 nm versus the concentration. At 600 nm, the monomeric species displays a significant absorption band, while the dimeric species shows much lower absorbance. To ensure favorable dimerization within a concentration range that results in absorbance values below 1, K_{dim} was determined in a mixture of THF/water of 6:4.

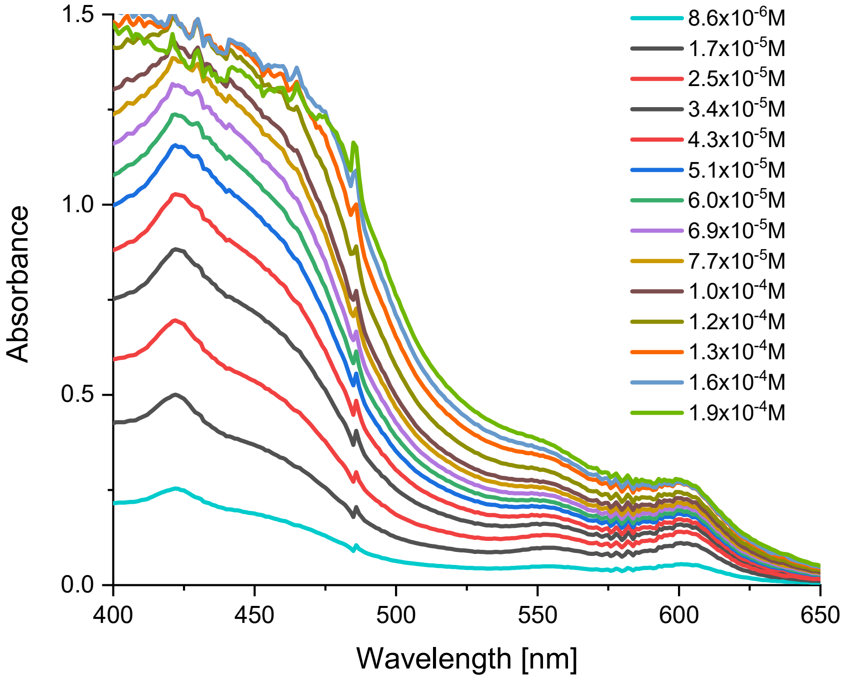

Figure S 17. UV-Vis spectra of *rac*-**7** at varying concentrations in THF/water (6:4).

The following equations were utilized for fitting the data:

The observed absorbance at 600 nm is

$$A = \varepsilon_M[M] + \varepsilon_D[D] \quad (1)$$

where ε_M and ε_D are the extinction coefficients of pure monomer and pure dimer in THF/water (6:4), and [M] and [D] are the concentrations of monomer and dimer, respectively.

Then,

$$K_{dim} = \frac{[D]}{[M]^2} \quad (2)$$

$$[D] = K_{dim}[M]^2 \quad (3)$$

$$C_{total} = [M] + 2[D] \quad (4),$$

where C_{total} is the known total concentration of the sample.

Putting equation (3) into equation (4) results in equation (5)

$$2K_{dim}[M]^2 + [M] - C_{total} = 0 \quad (5).$$

Solving equation (5) for [M] results in equation (6)

$$[M] = \frac{-1+\sqrt{1+8K_{dim}C_{total}}}{4K_{dim}} \quad (6).$$

Putting equation (6) into equation (3) gives equation (7)

$$[D] = K_{dim}\left(\frac{-1+\sqrt{1+8K_{dim}C_{total}}}{4K_{dim}}\right)^2 \quad (7).$$

Finally, putting equation (7) and equation (6) into equation (1) allows to fit the absorbance value versus concentration (Figure S18).

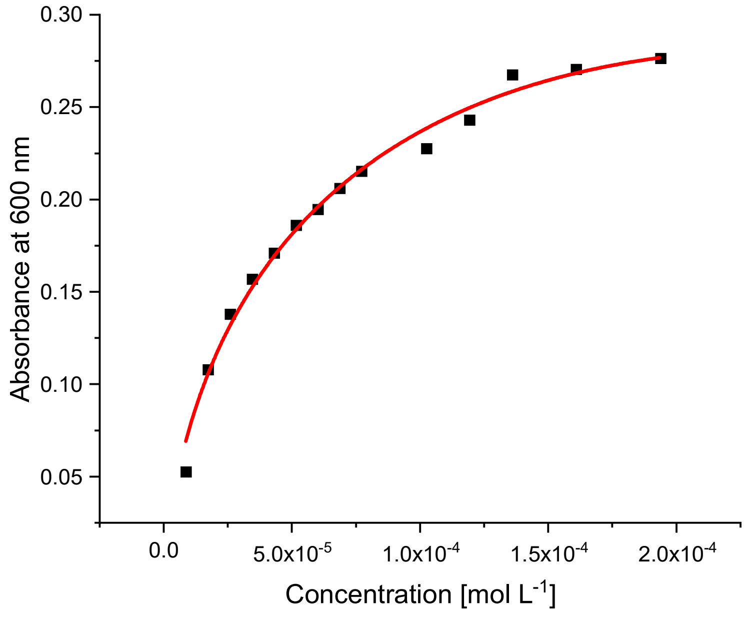

Figure S 18. Absorbance at 600 nm vs. concentration of *rac*-**7** in THF/water (6:4). The red line is the fit obtained according to equations (7), (6) and (1).

It should be noted that by using this method, the determined K_{dim} value relies on the extinction coefficients of pure monomer or dimer ε_M and ε_D, which are both unknown in THF/water (6:4) and can

also not experimentally be determined, because at any useful concentrations for UV-Vis measurements, *rac*-**7** is present as a mixture of both monomer and dimer. While we can use the assumption that the ε_M is similar in THF/water (6:4) as in pure THF (~15x10^4), we cannot make a comparable assumption for the dimeric species, which is why ε_D is fitted along with the K_{dim} value, resulting in a comparably large error. Also, the determined K_{dim} should rather serve as a rough estimate than a true dimerization constant.

Using this method, a dimerization constant of

$$K_{dim} = 7.6x10^4 \ \pm 4.6x10^3 M^{-1}$$

was determined.

The determination of the dimerization constant using ^{1}H NMR was unsuccessful due to complex peak overlap and broad dimeric peaks that made integrations unreliable, also when using peak deconvolution.

5. Chiral Resolution

Semipreparative separation of (*P*)-**2** and (*M*)-**2** was achieved on a CHIRALPAK IG column (10x250 mm, 5 µm particle size), using CH_2Cl_2 as an eluent with a flowrate of 5 mL/min at 40 °C column temperature. Assignment of the absolute configuration was done by comparing the measured and calculated CD spectra (see section 9 in the Supporting Information).

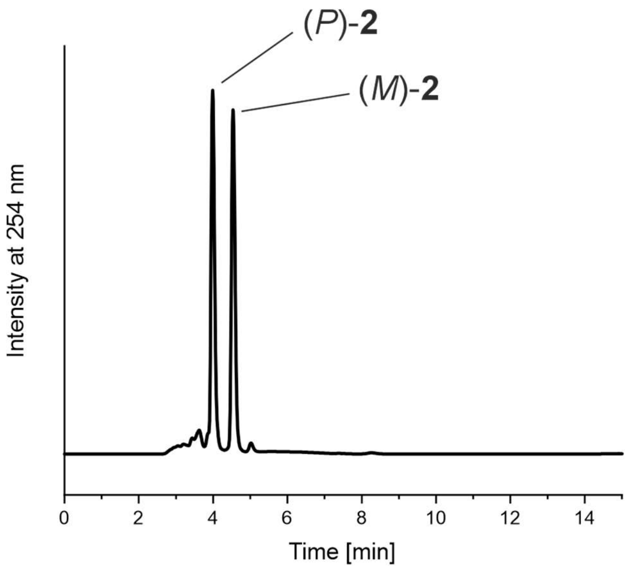

Figure S 19. HPLC chromatogram of *rac*-**2**.

Semipreparative separation of (*P*)-**6** and (*M*)-**6** was achieved on a CHIRALPAK IBN-5 column (10x250 mm, 5 µm particle size), using *n*-heptane as an eluent with a flowrate of 4.5 mL/min at 40 °C column temperature. The assignment of the absolute configuration was done by comparing the measured and calculated CD spectra (see section 9 in the Supporting Information).

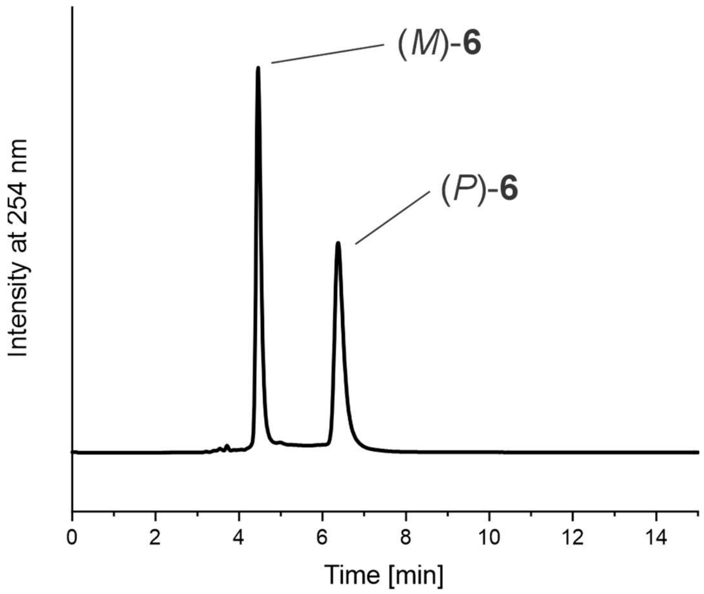

Figure S 20. HPLC chromatogram of *rac*-**6**.

6. Spectroscopy

For comparison, the absorption spectra of *rac*-**1**, *rac*-**2**, and *rac*-**4** were recorded.

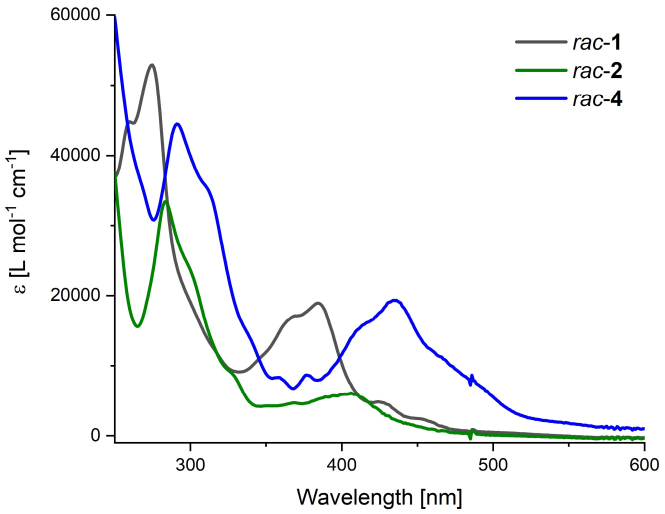

Figure S 21. UV-Vis spectra of *rac*-**1** (black), *rac*-**2** (green), and *rac*-**4** (blue) in CH_2Cl_2 (c ~ 10^{-5}M).

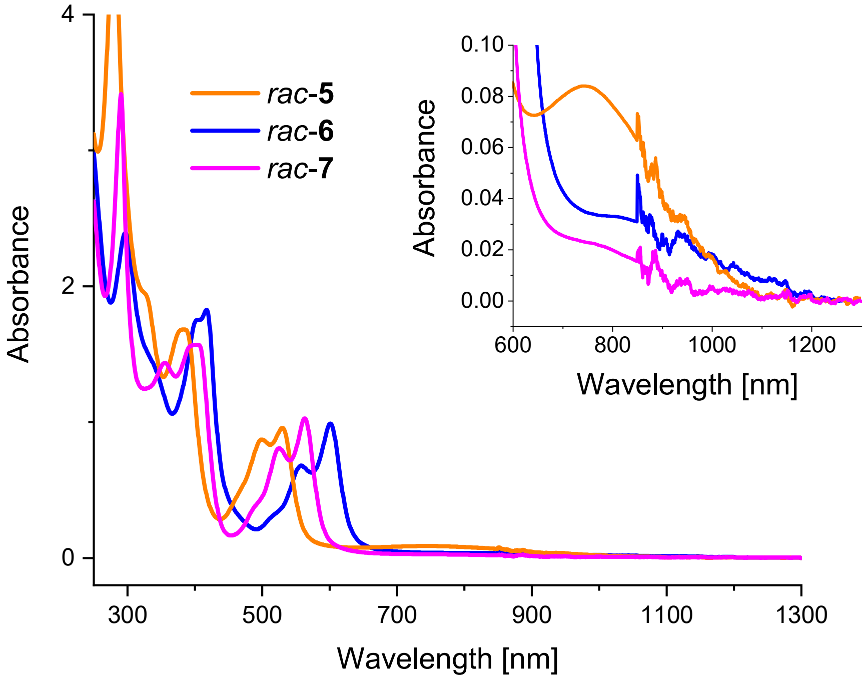

Figure S 22. Concentrated UV-Vis spectra of *rac*-**5** (orange), *rac*-**6** (pink) and *rac*-**7** (blue), showing the broad but very weak absorption band of *rac*-**5** in the inset, which is absent for *rac*-**6** and *rac*-**7**.

CD spectra of (*P*)-**2** and (*M*)-**2** were measured in CH_2Cl_2. Assignment of absolute configuration was performed by comparing the measured and calculated CD spectra (see section 9 in the Supporting Information). The absorption dissymmetry factor (Δε/ε) spectra were calculated by dividing the respective molar circular dichroism values by the extinction coefficients.

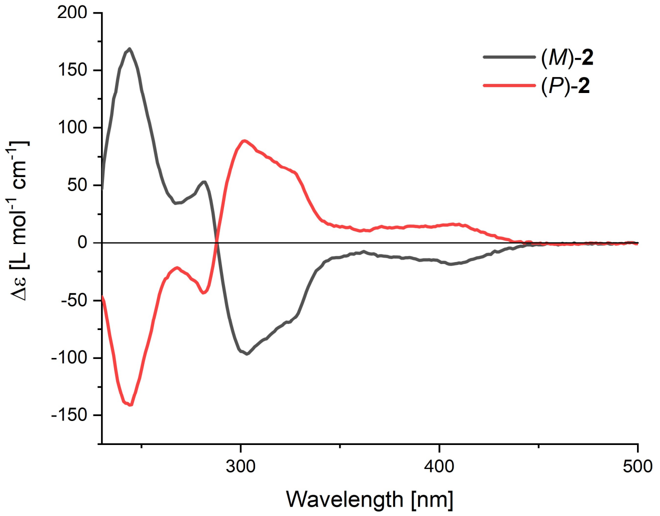

Figure S 23. CD spectra of (*M*)-**2** (black) and (*P*)-**2** (red) in CH_2Cl_2 (c ~ 10^{-5} M).

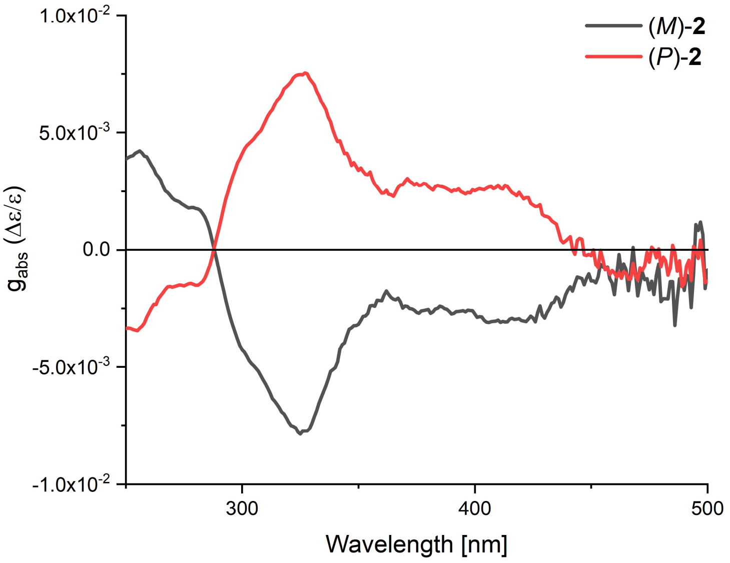

Figure S 24. g_{abs} plot of (*M*)-**2** (black) and (*P*)-**2** (red).

CD spectra of (*P*)-**6** and (*M*)-**6** were measured in *n*-heptane directly after isolation via HPLC due to their quick racemization. Assignment of absolute configuration was done by comparing the measured and

calculated CD spectra (see section 9 in the Supporting Information). The absorption dissymmetry factor spectra were calculated by dividing the respective CD spectra by the UV-Vis spectra.

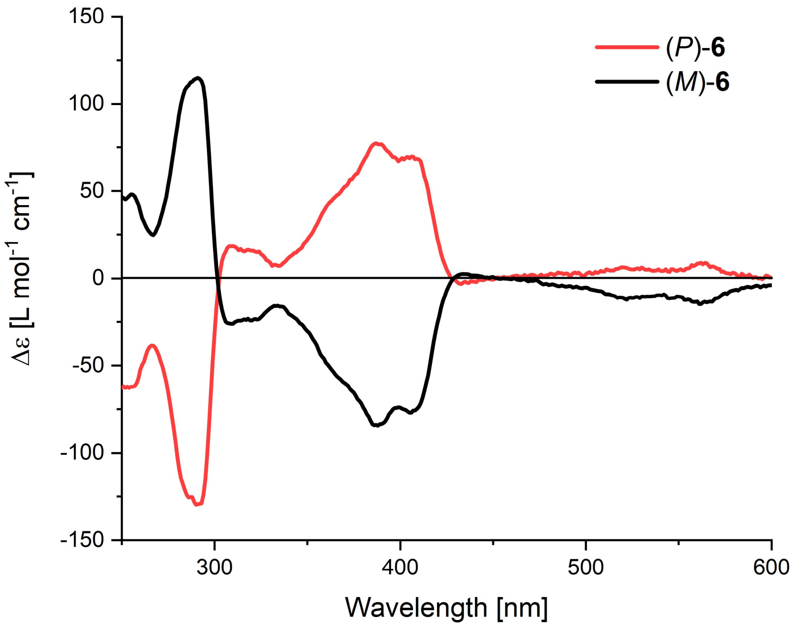

Figure S 25. CD spectra of (*M*)-**6** (black) and (*P*)-**6** (red) in *n*-heptane (c ~ 10^{-5} M).

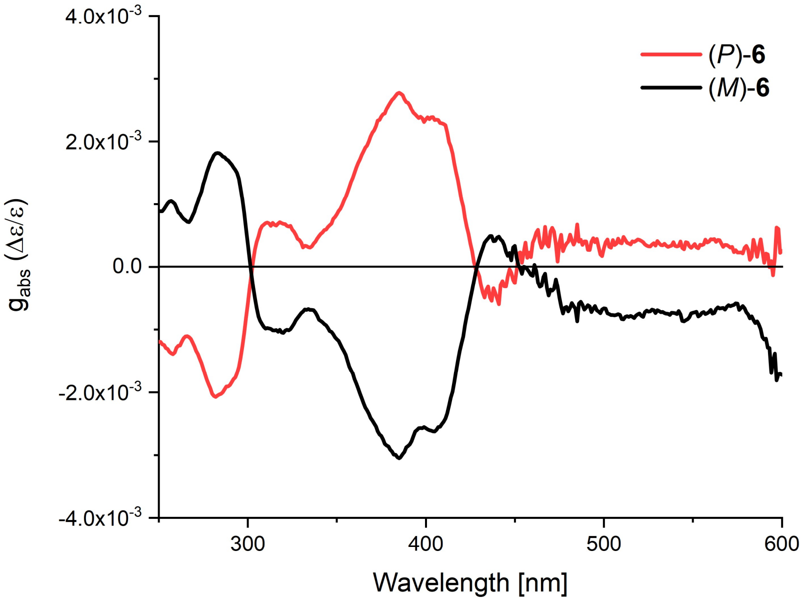

Figure S 26. g_{abs} plot of (*M*)-**2** (black) and (*P*)-**6** (red).

7. Enantiomerization Barrier

The enantiomerization barrier of **2** was measured by subjecting (*P*)-**2** to dynamic CD spectroscopy at 60 °C in 1,2-dichloroethane (DCE) and recording the time-dependent ellipticity at 305 nm (Figure S27).

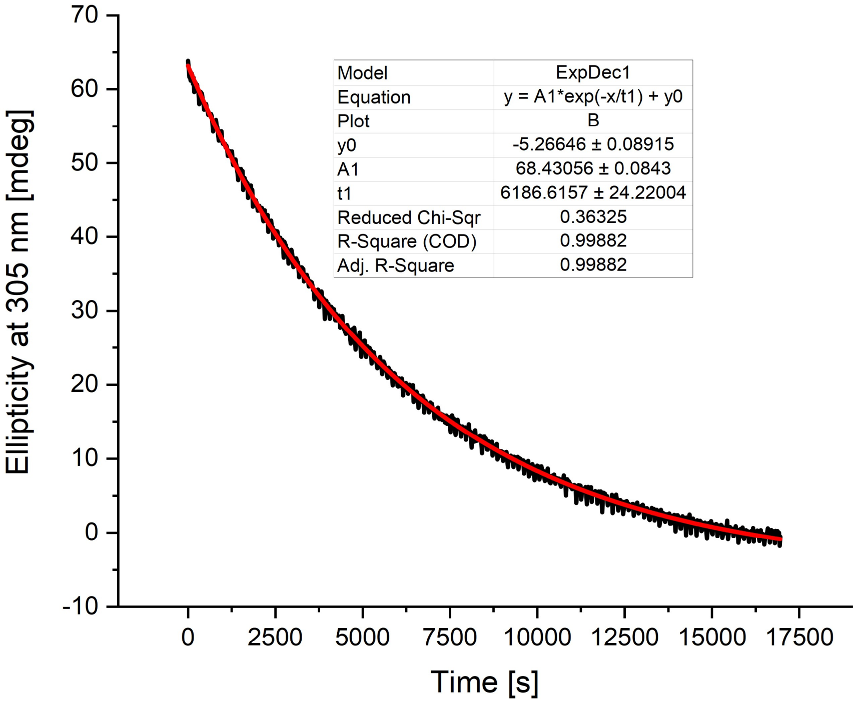

Figure S 27. Time-dependent CD signal of (*P*)-**2** at 305 nm in DCE at 60 °C.

Using the equations[8]

$$k_{rac} = \frac{1}{t1}$$

$$k_{rac} = 2k_e$$

$$\Delta G^{\ddagger}_{e(333.15} = -RTln\left(\frac{hk_e}{Tk_B}\right)$$

we calculated an enantiomierzation barrier of $\Delta G^{\ddagger}_{e(333.15)} = 108\ kJ/mol$ for **2** at 60 °C in DCE. This corresponds to a half-life at room temperature of $t_{1/2}$ = 23.5 d.

The enantiomerization barrier of **6** was obtained by dynamic chromatography at 70 °C, using the same separation conditions as described in section 5 in the Supporting Information. The elution profile (Figure S28) was analyzed with the DCXplorer software[9], which gave an enantiomerization reaction rate of k_e = 6.33x10^{-3} s^{-1}.

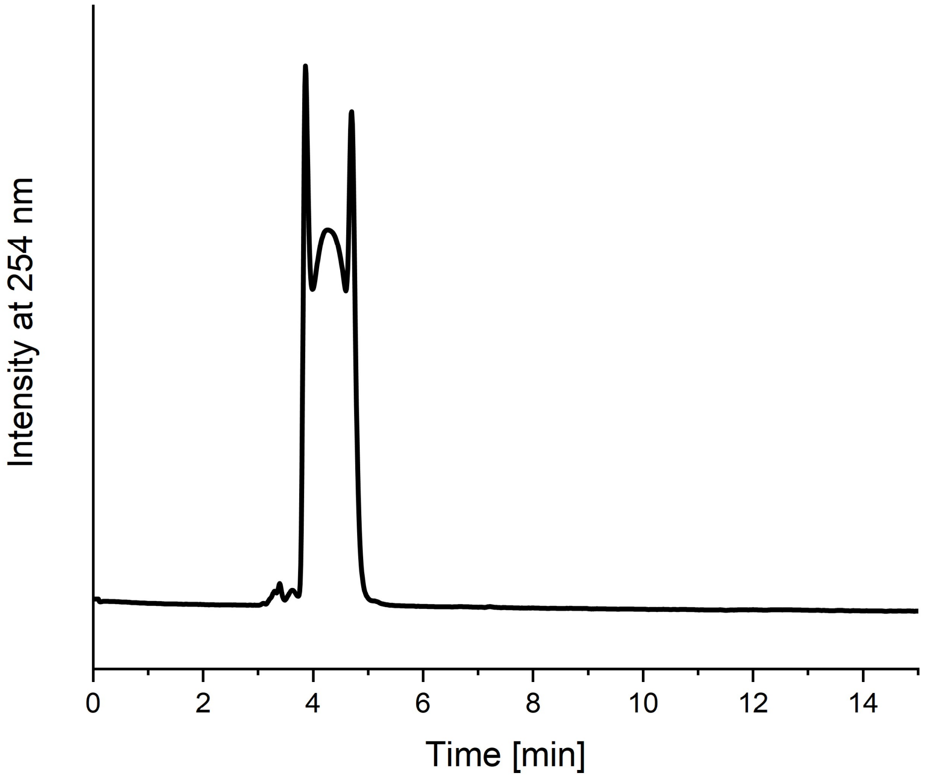

Figure S 28. HPLC chromatogram of *rac*-**6** at 70 °C column temperature.

Using

$$\Delta G^{\ddagger}_{e(343.15)} = RT[\ln(\frac{k_B T}{h}) - \ln(k_e)]$$

we obtained an enantiomerization barrier of $\Delta G^{\ddagger}_{e(343.15)} = 99\ kJ/mol$ for **6** at 70 °C under the HPLC separation conditions. This corresponds to a half-life at room temperature of $t_{1/2}$ = 14 h.

8. Electrochemistry

All cyclized IF derivatives were analysed at 0.5 mM in Ar-degassed, anhydrous CH_2Cl_2, containing 100 mM $TBAPF_6$ as electrolyte. Due to the aggregation behaviour of *rac*-**7**, solutions of this compound were aged for up to 3 days prior to measurement, until a deep-blue colouration was observed, indicative of the monomeric species. The GC disk electrode was polished with 0.05 µm alumina slurry prior to each set of experiments. For both cyclic voltammetry (CV) and square-wave voltammetry (SWV) measurements a step potential of 2 mV was used. SWV was carried out at 20 Hz and an amplitude of 20 mV. No iR compensation was employed.

As shown in Figure S29 and S30, for all three derivatives four (quasi)reversible redox-couples were observed. The small shoulder peaks marked with an asterisk in Figure S29 arise from the non-ideal reversibility of the Ox2 (or for *rac*-**7** also Red2) process. In both cases this peak is not observed when the voltage scanning range is restricted to Ox1 (and Red1 for *rac*-**7**), confirming that these peaks do not arise from an impurity. Note also that the Ox2 peak starts to overlap with background oxidation.

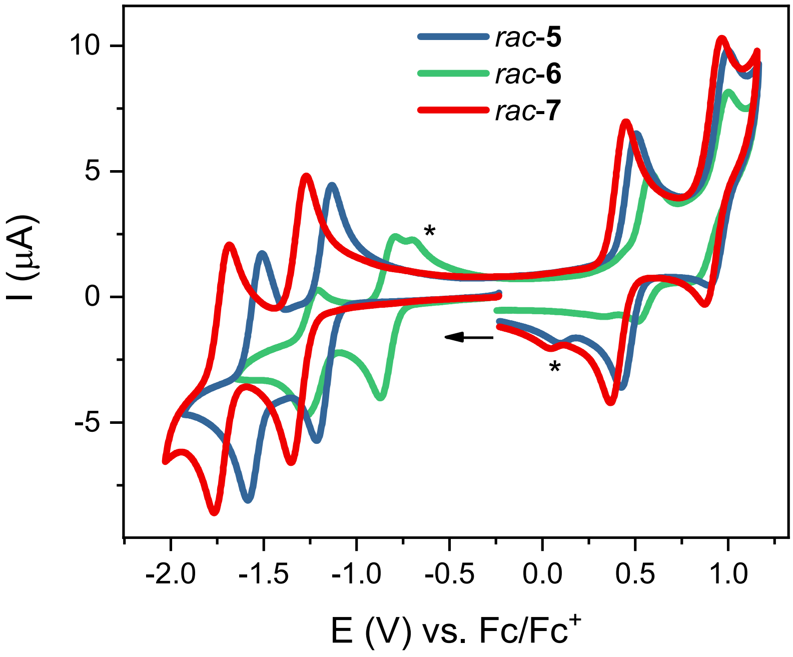

Figure S 29. CVs of *rac*-**5**, *rac*-**6**, and *rac*-**7** at v = 100 mV/s in CH_2Cl_2, 100 mM $TBAPF_6$. The black arrow indicates the starting point and direction of the first scan. The peak marked with an asterisk is not an impurity but is a result of the second oxidation wave.

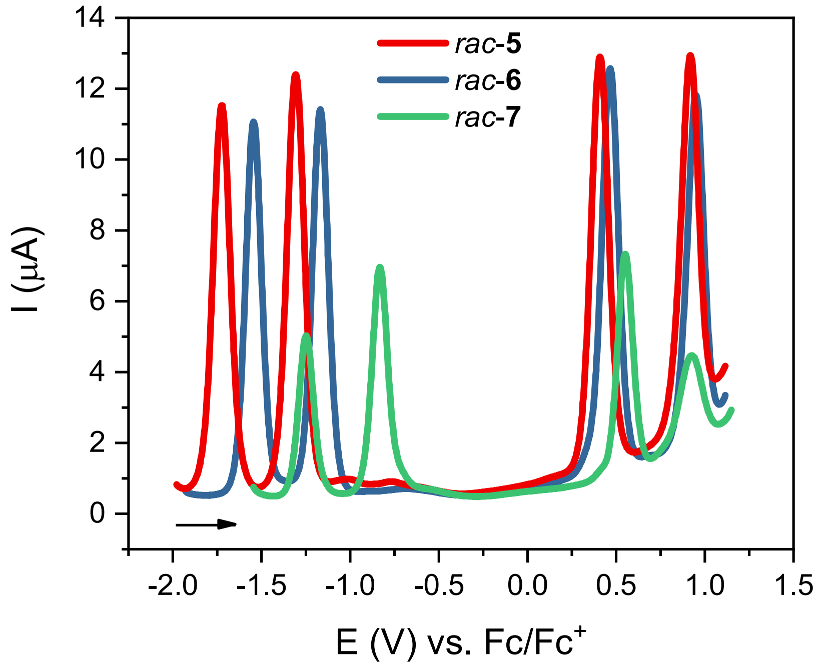

Figure S 30. SWVs of *rac*-**5**, *rac*-**6**, and *rac*-**7** in CH_2Cl_2, 100 mM $TBAPF_6$. The black arrow indicates the direction of the scan.

Table S 2. Half-wave potentials $E_{1/2}$ and peak separations ΔE_p (at ν = 25 mV/s) of *rac*-**5**, *rac*-**6**, and *rac*-**7** in CH_2Cl_2, 100 mM $TBAPF_6$ vs Fc/Fc^+.

	**Red2**		**Red1**		**Ox1**		**Ox2**	
	**$E_{1/2}$ (V)**	**ΔE (mV)**	**$E_{1/2}$ (V)**	**ΔE (mV)**	**$E_{1/2}$ (V)**	**ΔE (mV)**	**$E_{1/2}$ (V)**	**ΔE (mV)**
rac-**5**	–1.720[a]	76	–1.305[a]	75	+0.410[a]	77	+0.925[b]	104
rac-**6**	–1.550[a]	67	–1.165[a]	72	+0.470[a]	76	+0.955[b]	115
rac-**7**	–1.245[b]	100	–0.830[a]	76	+0.555[b]	98	+0.935[b]	n/a

a – reversible, b – quasireversible; the reported potentials correspond to the peak potentials obtained via SWV.

Table S 3. Differences in $E_{1/2}$ of *rac*-**6** and *rac*-**7** relative to *rac*-**5**. Values in parentheses are wrt. *rac*-**6**.

	**Red2**	**Red1**	**Ox1**	**Ox2**
rac-**5**	≡ 0	≡ 0	≡ 0	≡ 0
rac-**6**	+170 mV	+140 mV	+60 mV	+30 mV
rac-**7**	+475 mV (+305 mV)	+475 mV (+335 mV)	+145 mV (+100 mV)	+10 mV (–20 mV)

The significantly larger effect of substitution at the IF core (R-position) on the redox potentials can be explained by consideration of the conjugation pattern/mesomeric structures of the different charge states (Scheme S2); the R substituent is directly attached to the position in which the charge localisation is expected to be highest. In contrast, no relevant mesomeric structure contains a charge adjacent to the R′ position, such that the electron-withdrawing effect of the TIPS-acetylene groups has much more impact when placed in the R position.

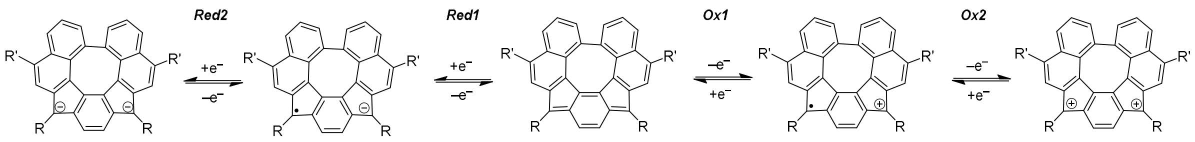

Scheme S 2. Depiction of the 5 charge states of the derivatives and their proposed, most relevant mesomeric structures.

Further data and discussions regarding reversibility

As mentioned in the main text, Red2, Red1 and Ox1 for both *rac*-**5** and *rac*-**6** are reversible redox couples, as evidenced by a near-unity ratio of peak currents, a low peak separation of <77 mV in all cases (Table S2) and a linear dependence of the peak currents on the square-root of the scan rate across a large range of scan rates, as predicted by the Randles-Sevcik equation, see Figures S31-38. This also confirms that all redox processes are diffusion controlled. Ox2 is somewhat less reversible in both cases, as indicated by higher peak separation and non-unity peak current ratios.

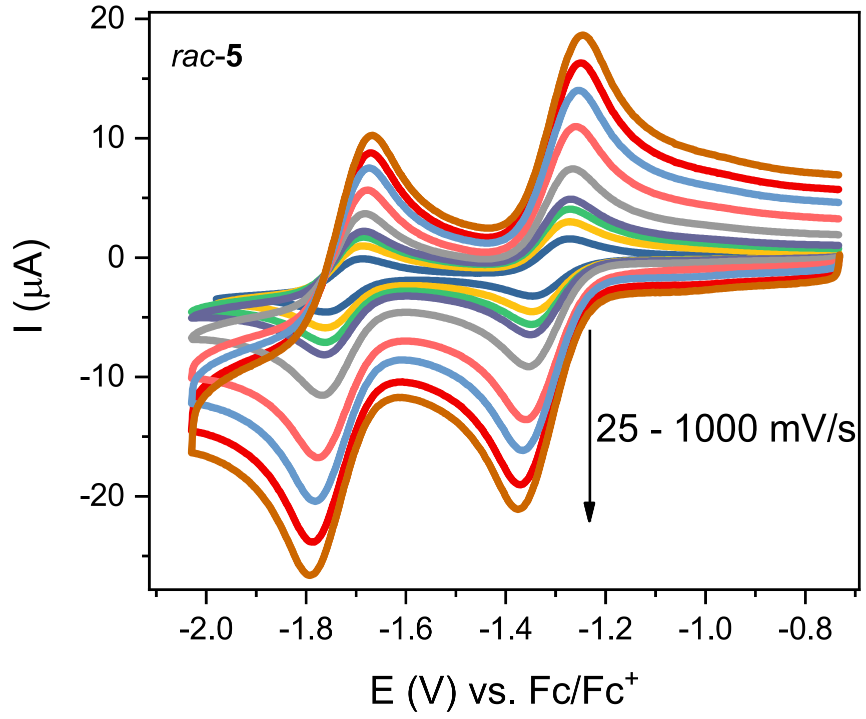

Figure S 31. CVs of *rac*-**5** at varying scan rates in CH_2Cl_2, 100 mM $TBAPF_6$ of the first and second reductive couple.

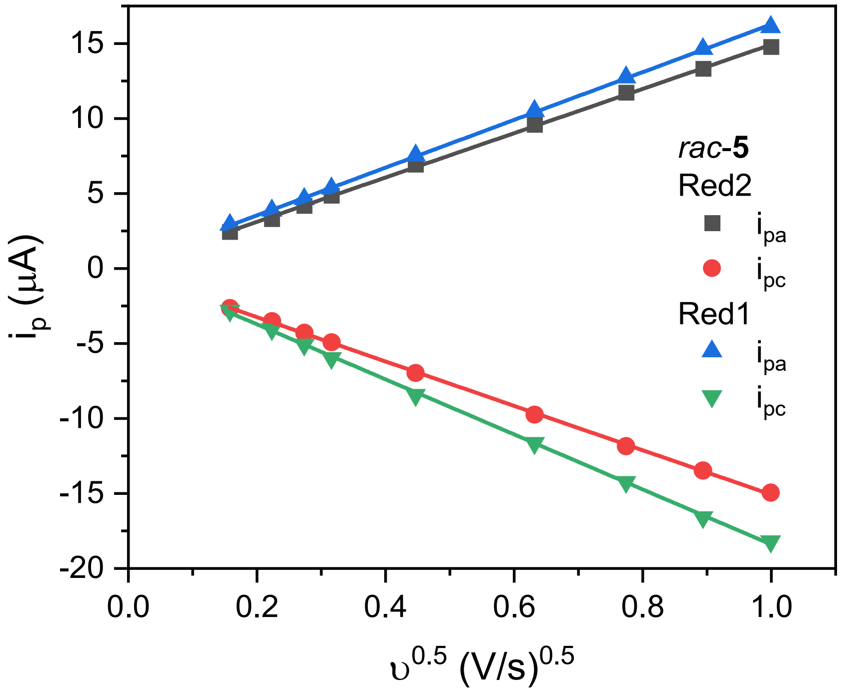

Figure S 32. Anodic and cathodic peak currents for the reductive couples of *rac*-**5** as a function of the square-root of the scan rate, including linear fits, in CH_2Cl_2, 100 mM $TBAPF_6$.

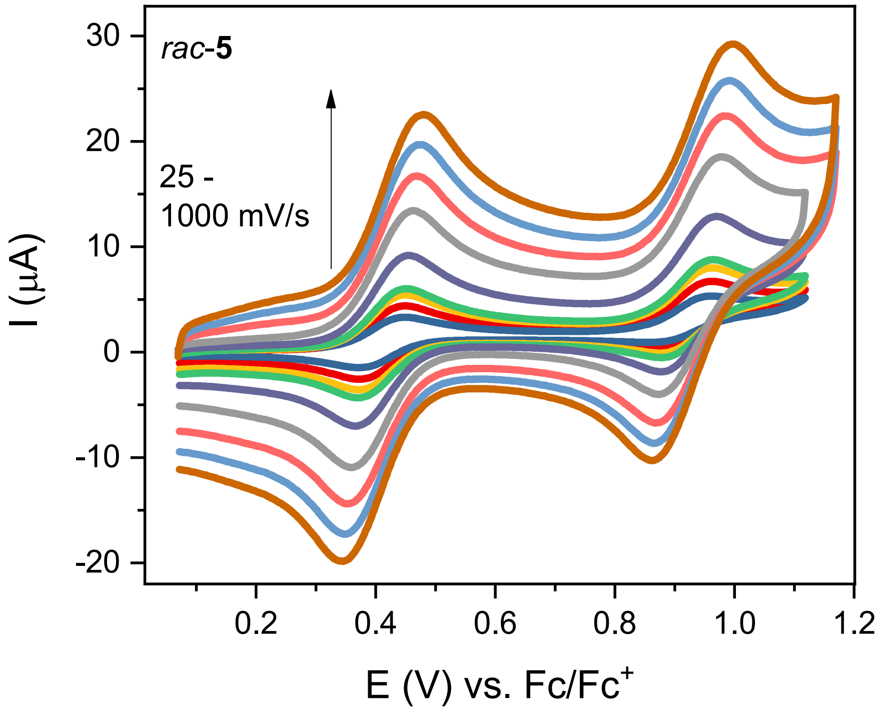

Figure S 33. CVs of *rac*-**5** at varying scan rates in CH_2Cl_2, 100 mM $TBAPF_6$ of the first and second oxidative couple.

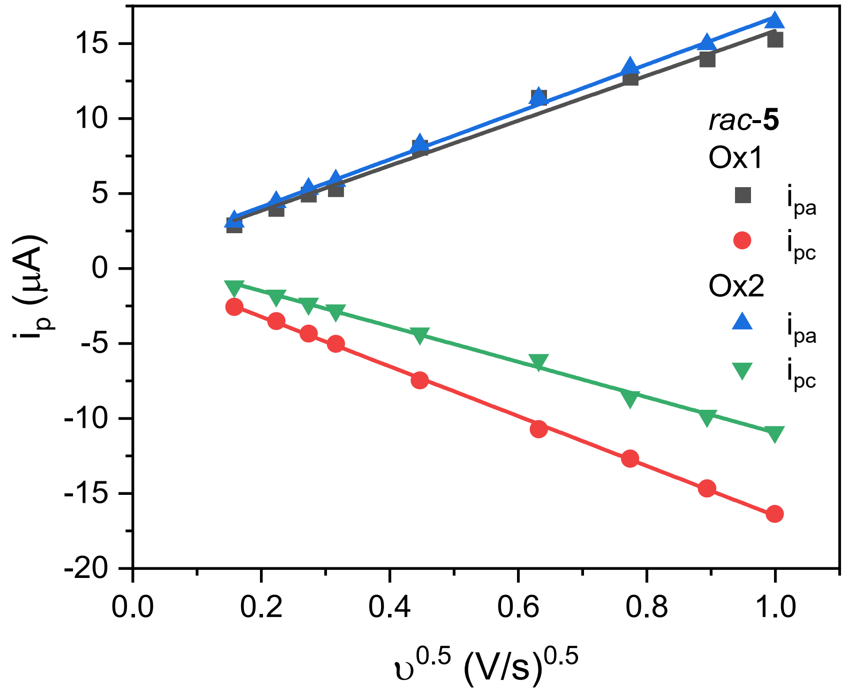

Figure S 34. Anodic and cathodic peak currents for the oxidative couples of *rac*-**5** as a function of the square-root of the scan rate, including linear fits, in CH_2Cl_2, 100 mM $TBAPF_6$.

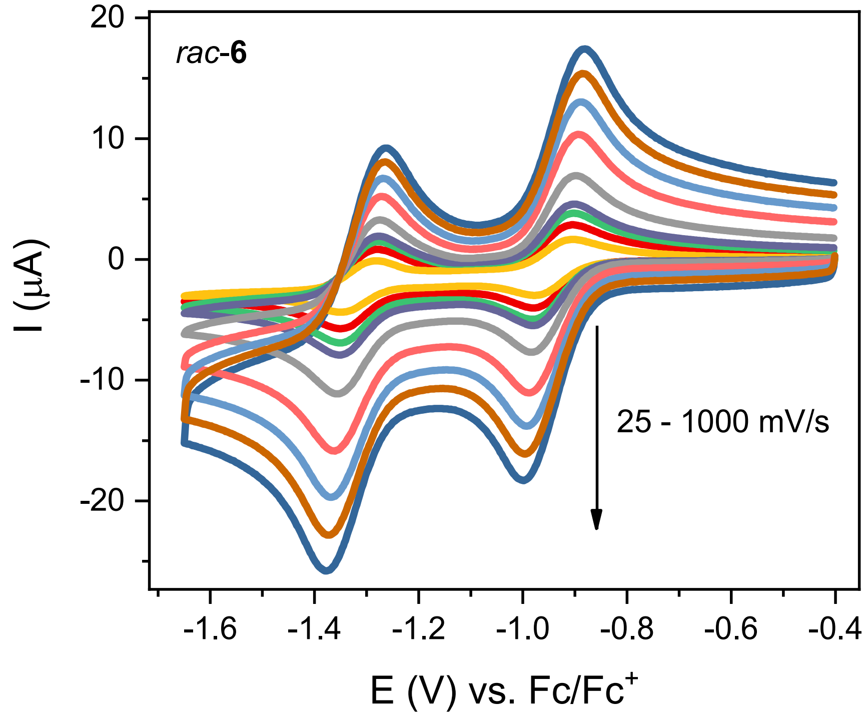

Figure S 35. CVs of *rac*-**6** at varying scan rates in CH_2Cl_2, 100 mM $TBAPF_6$ of the first and second reductive couple.

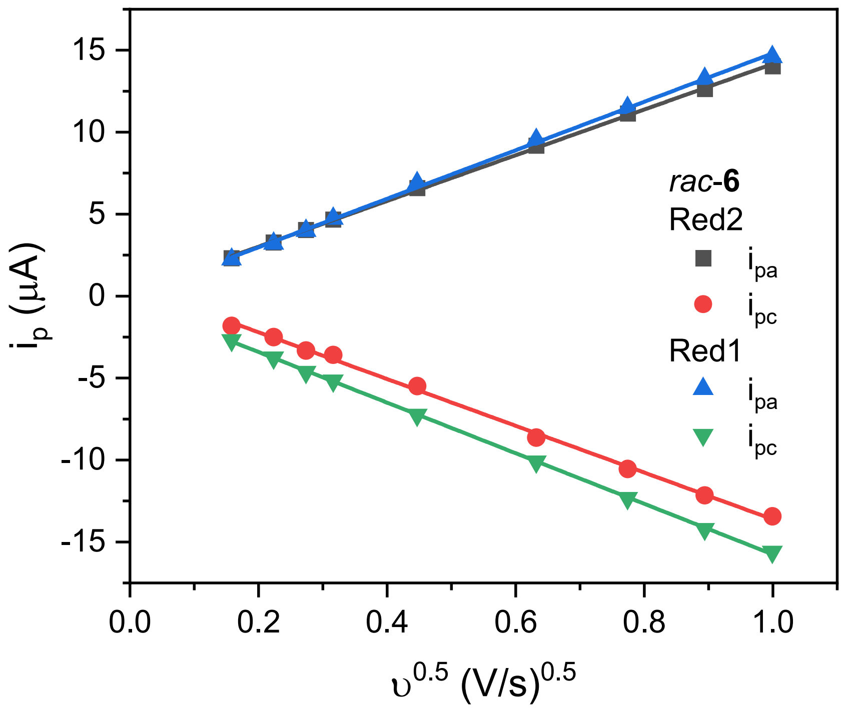

Figure S 36. Anodic and cathodic peak currents for the reductive couples of *rac*-**6** as a function of the square-root of the scan rate, including linear fits, in CH_2Cl_2, 100 mM TBAPF6.

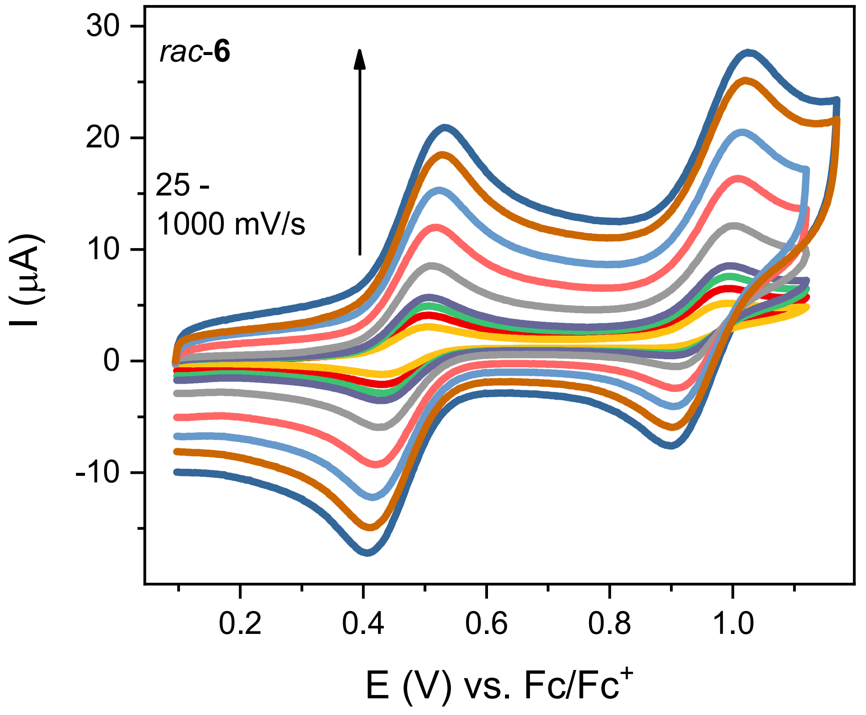

Figure S 37. CVs of *rac*-**6** at varying scan rates in CH_2Cl_2, 100 mM $TBAPF_6$ of the first and second oxidative couple.

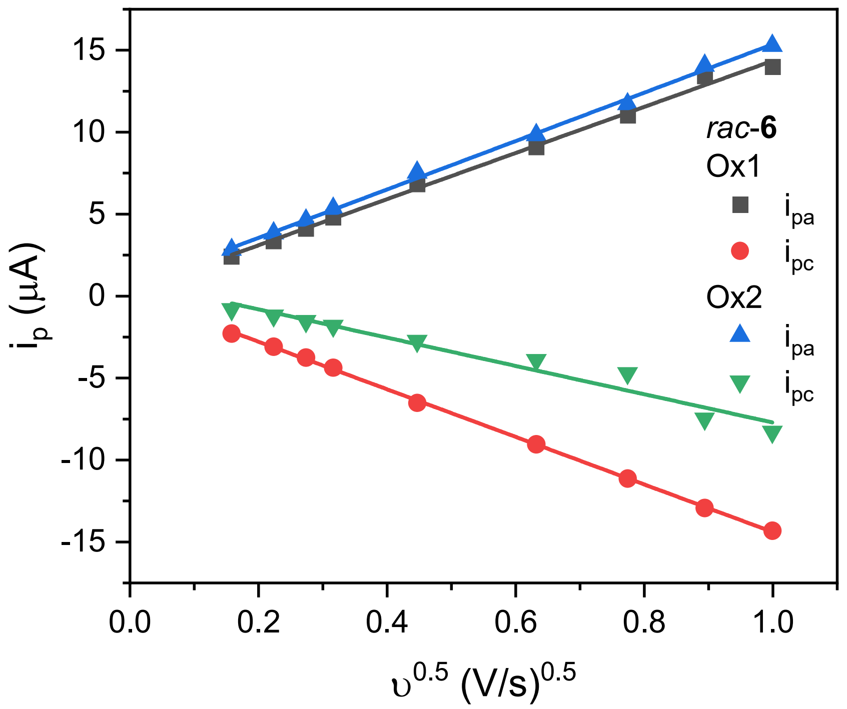

Figure S 38. Anodic and cathodic peak currents for the oxidative couples of *rac*-**6** as a function of the square-root of the scan rate, including linear fits, in CH_2Cl_2, 100 mM $TBAPF_6$.

For *rac*-**7**, Ox2 and Red2 display a much lower degree of (quasi)reversibility, as indicated by higher peak separations and non-unity peak current ratios. Only Red1 is reversible (Figures S39-40), while Ox1 is quasi-reversible (Figures S41-42), as evidenced by a slightly larger peak separation (98 mV at ν = 25 mV/s). This is potentially reflective of the fact that the smaller TIPS-acetylene groups, in contrast to the mesityl groups, do not sterically block the generated charge states from intermolecular reactions and/or dimerization.

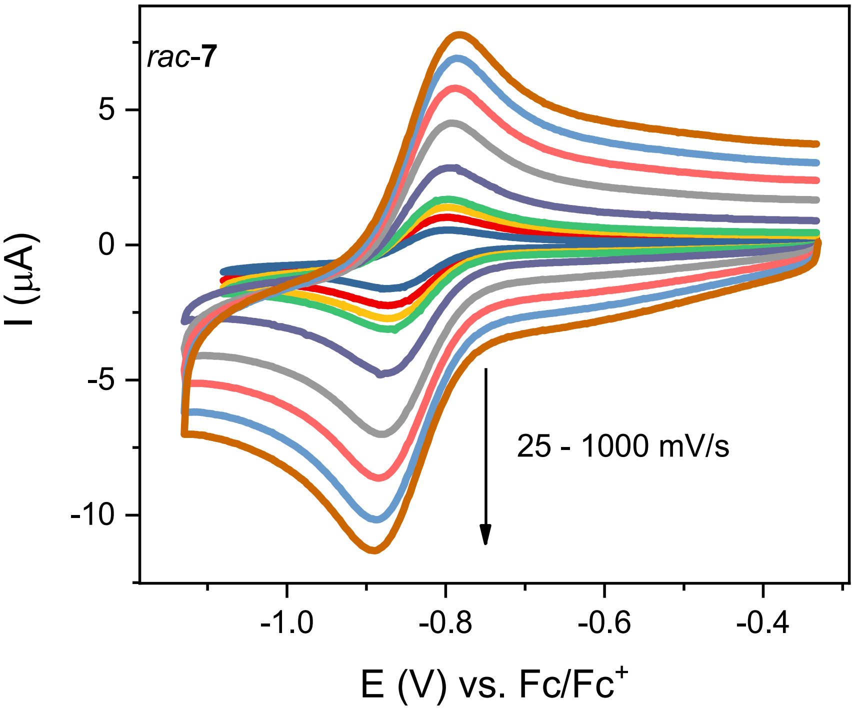

Figure S 39. CVs of *rac*-**7** at varying scan rates in CH_2Cl_2, 100 mM $TBAPF_6$ of the first reductive couple.

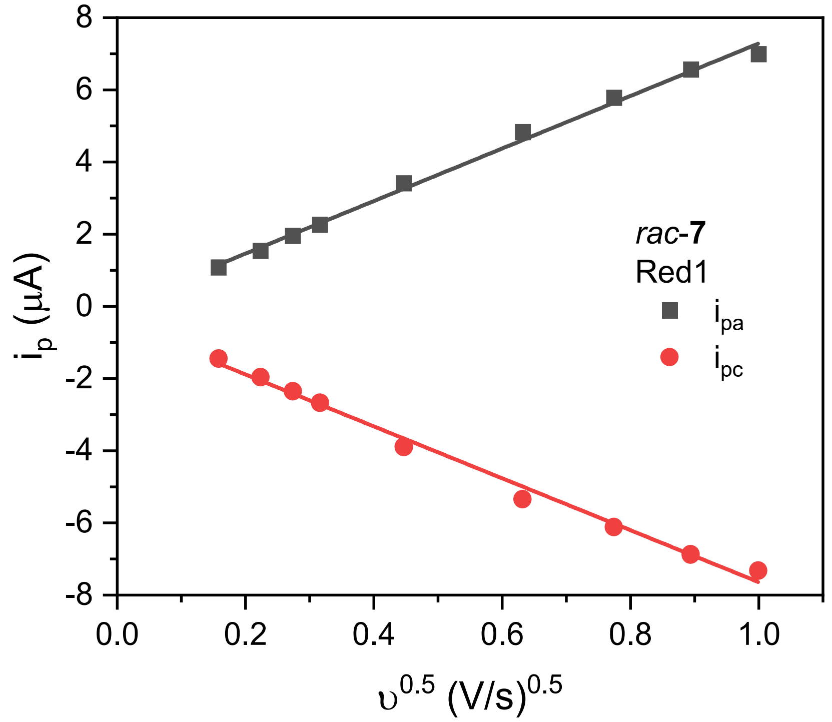

Figure S 40. Anodic and cathodic peak currents for the first reductive couple of *rac*-**7** as a function of the square-root of the scan rate, including linear fits, in CH_2Cl_2, 100 mM $TBAPF_6$.

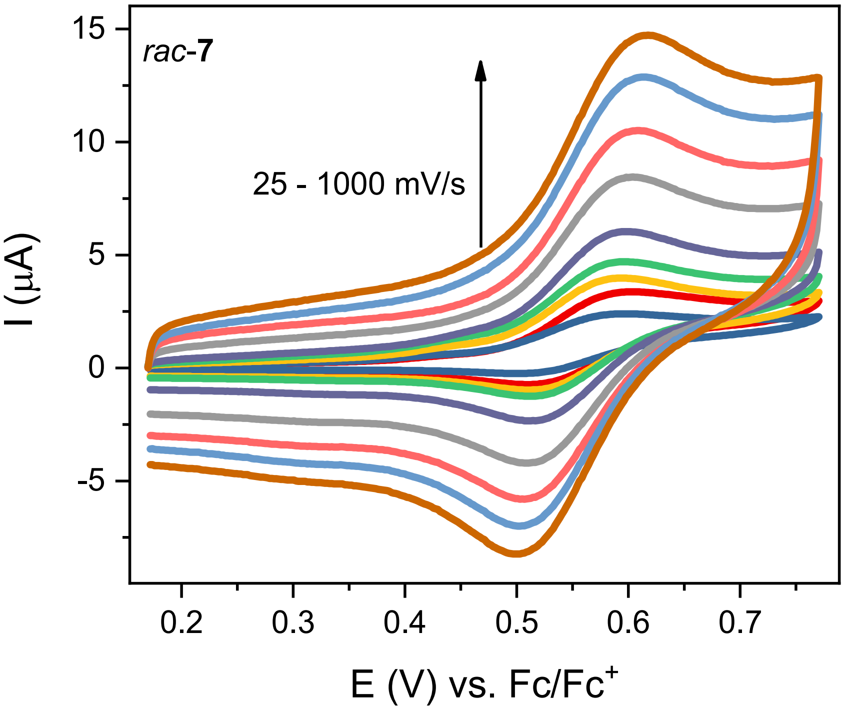

Figure S 41. CVs of *rac*-**7** at varying scan rates in CH_2Cl_2, 100 mM $TBAPF_6$ of the first oxidative couple.

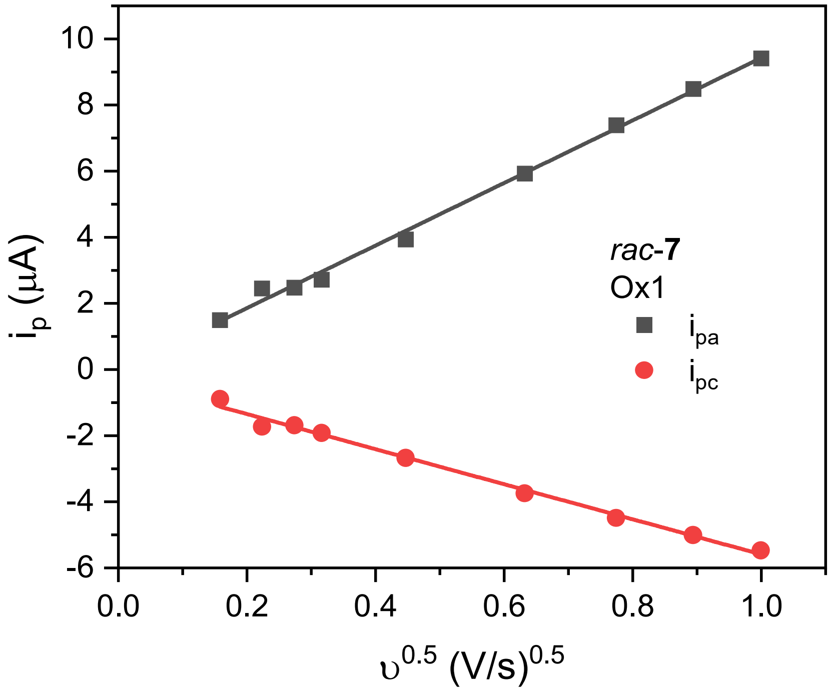

Figure S 42. Anodic and cathodic peak currents for the first oxidative couple of *rac*-**7** as a function of the square-root of the scan rate, including linear fits, in CH_2Cl_2, 100 mM $TBAPF_6$.

9. Computations

To reduce computational demand, the (triisopropylsilyl)acetylene (TIPS) was simplified to (trimethylsilyl)acetylenes (TMS) for the calculations of (*M*)/(*P*)-**6** and (*M*)/(*P*)-**7**. It has been previously shown that this has negligible influence on certain calculated properties, such as the NICS-XY scan (*vide infra*).[10–12]

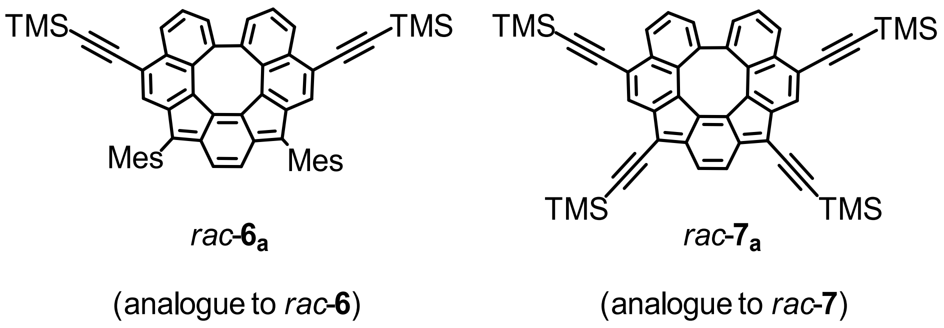

Scheme S 3. Chemical structures of the analogues for *rac*-**6** and *rac*-**7** used for computational analyses.

Structure Optimizations

MM2 optimized structures of (*M*)/(*P*)-**2**, (*M*)/(*P*)-**5**, (*M*)/(*P*)-**6**, and (*M*)/(*P*)-**7** were further optimized through DFT calculations at a r^{2}SCAN-3c/CPCM(CH_2Cl_2)[13,14] level of theory. The potential energy minima were confirmed by subsequent frequency analysis, showcasing zero negative vibrational frequencies.

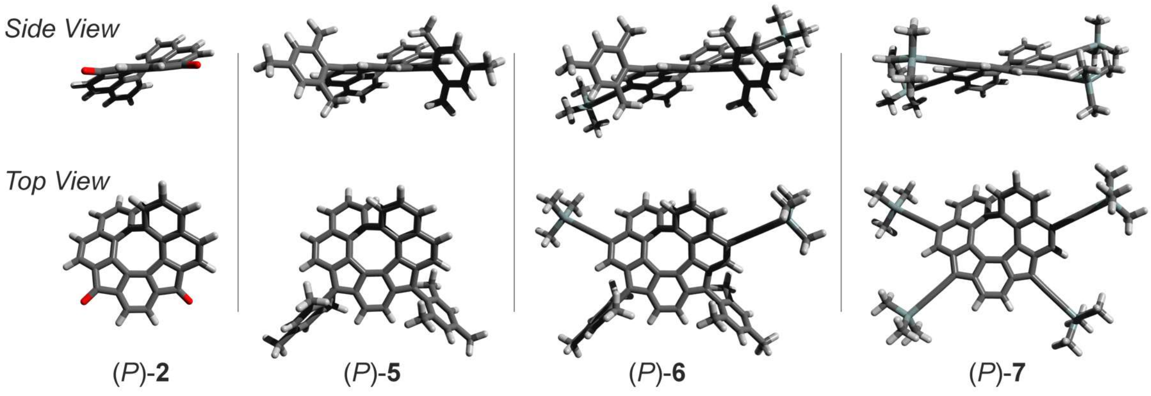

Figure S 43. Side and top view of the DFT-optimized structures of (*P*)-**2**, (*P*)-**5**, (*P*)-**6**, and (*P*)-**7**.

Enantiomerization Barrier

The transition states for the enantiomerization of **2**, **5**, **6**, and **7** were calculated using the Nudged Elastic Band with Transition State optimization (NEB-TS) of the ORCA package. The transition state structures were then further optimized at a r^{2}SCAN-3c/CPCM(CH_2Cl_2)[13,14] level of theory and confirmed by the presence of a single imaginary harmonic frequency.

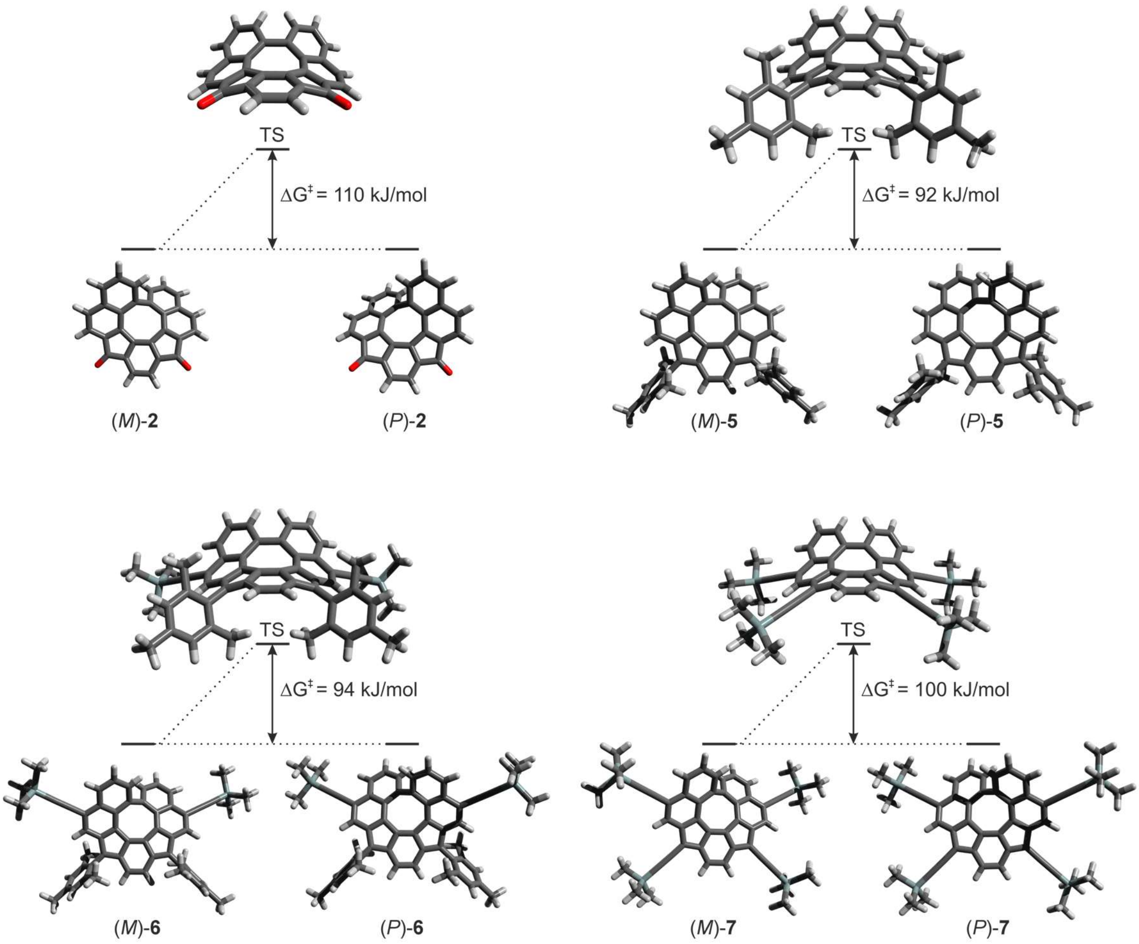

Figure S 44. Overview of the transition state structures and energies for the enantiomerization coordinates of **2**, **5**, **6**, and **7**.

TD-DFT

TD-DFT calculations of the DFT-optimized structures of (*P*)-**2**, (*P*)-**5**, (*P*)-**6** and (*P*)-**7** at the CAM-B3LYP/def2-TZVP/CPCM(CH_2Cl_2)[15,16] level of theory (with Tamm-Dancoff approximation and considering 30 singlet transitions) were performed to simulate the CD spectra for assigning the absolute configuration (for (*P*)-**2** and (*P*)-**6**) and to simulate the absorption spectrum (for (*P*)-**5**, (*P*)-**6** and (*P*)-**7**).

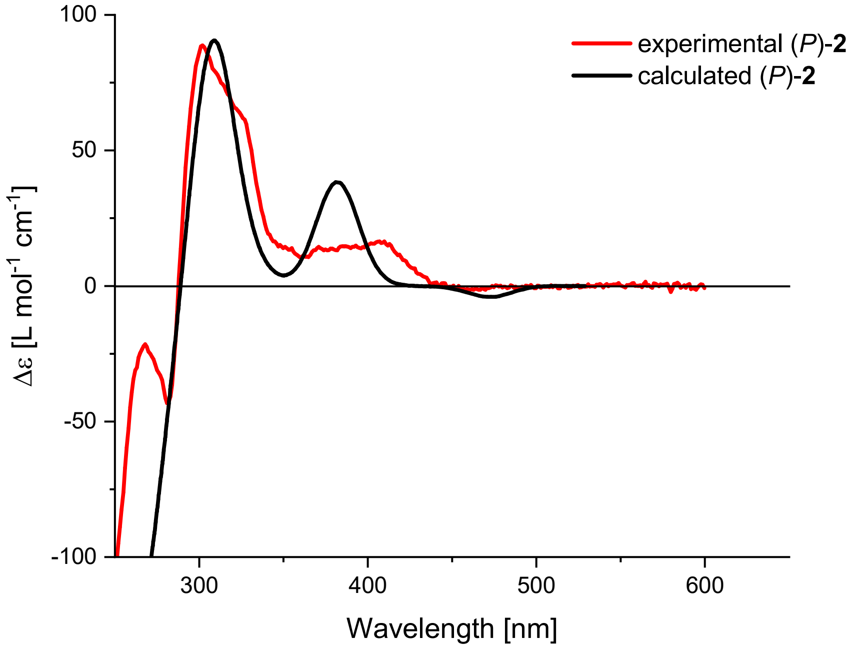

Figure S 45. Overlay of the experimental (red) and calculated (black) CD spectra of (*P*)-**2**. The calculated spectrum was shifted by +62 eV.

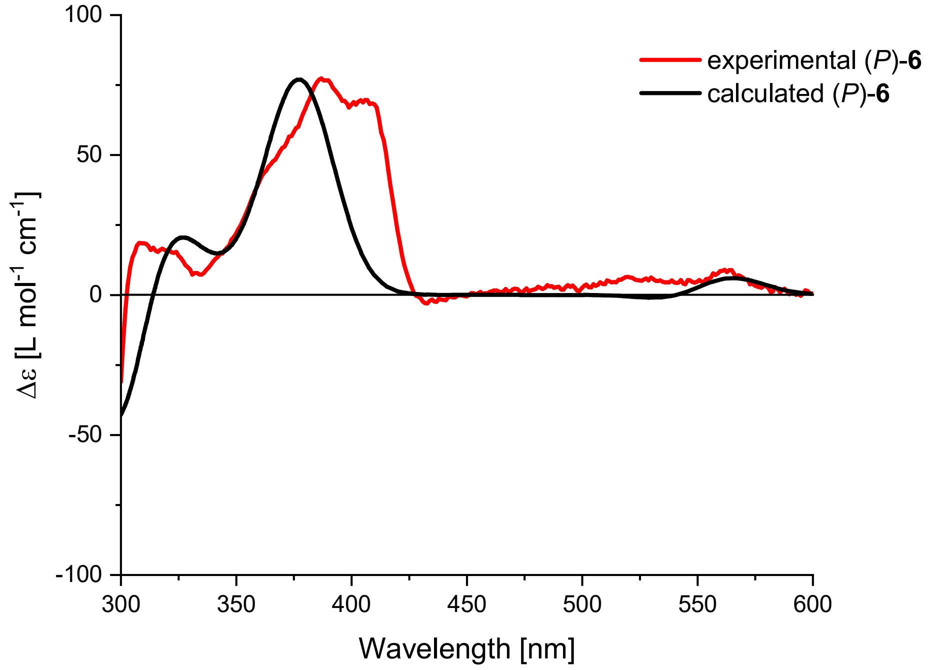

Figure S 46. Overlay of the experimental (red) and calculated (black) CD spectra of (*P*)-**6**. The calculated spectrum was shifted by +62 eV.

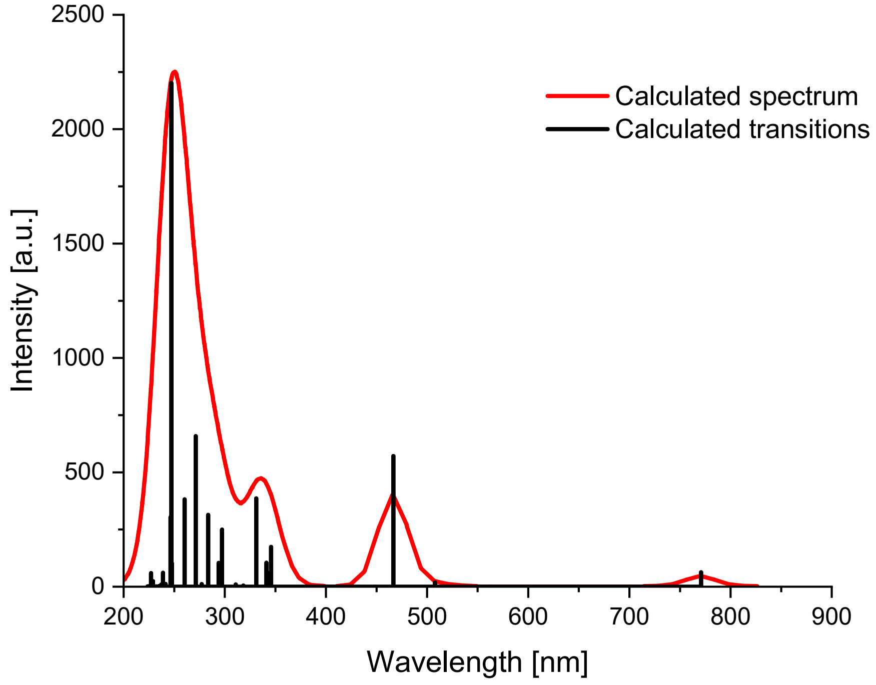

Figure S 47. Calculated absorption spectrum (red) of the calculated transitions (black) of *rac*-**5** using TD-DFT.

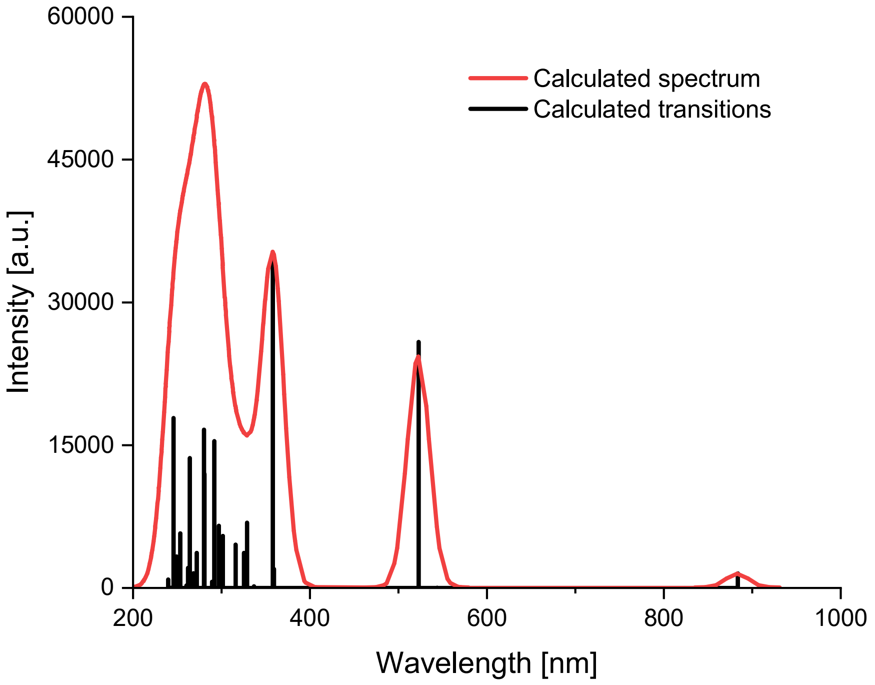

Figure S 48. Calculated absorption spectrum (red) of the calculated transitions (black) of *rac*-**6** using TD-DFT.

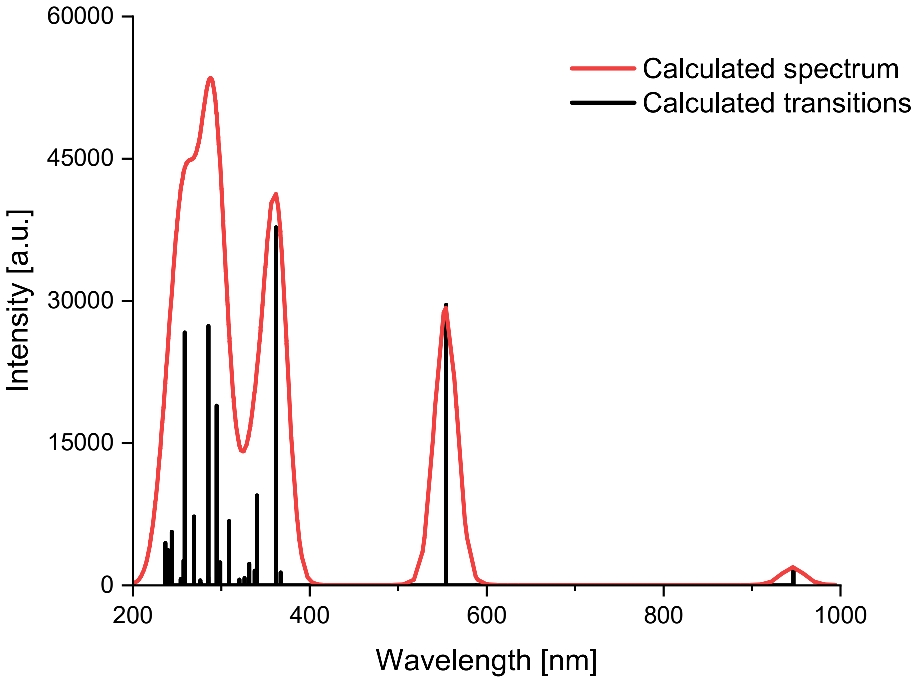

Figure S 49. Calculated absorption spectrum (red) of the calculated transitions (black) of *rac*-**7** using TD-DFT.

NICS-Calculations

NICS$_{\pi zz}$-XY scans[17–19] were performed on the DFT-optimized structures of (*P*)-**5**, (*P*)-**6**, and (*P*)-**7** at the B3LYP/6-311+G* NMR=GIAO[20–22] level of theory. The ghost atoms (Bq) were placed 1.7 Å above the rings using the Aroma package.[23] The inter-ghost-atom distance was set to 0.1 Å. Due to the symmetry axis of all studies indenofluorene compounds, only half of the molecule was scanned.

NICS(1.7) values on top of the 8-membered ring were calculated for (*P*)-**5**, (*P*)-**6**, and (*P*)-**7** at the B3LYP/6-311+G* NMR=GIAO[20–22] level of theory.

Diradical Character

The diradical characters y_0 of (*P*)-**5**, (*P*)-**6**, and (*P*)-**7** were determined by calculating the occupation numbers of the natural orbitals (n_{HONO} and n_{LUNO}) at the UKS B3LYP def2-TZVP level of theory, according to

$$T_i = \frac{n_{HON} \quad - n_{LUNO}}{2}$$

$$y_0 = 1 - \frac{2T_i}{1 + T_i^2}$$

as reported in literature.[24,25]

Table S 4. Summary of the occupation numbers of the HONO and LUNO, along with the resulting diradical character y_0.

Compound	n_{HONO}	n_{LUNO}	Y_0
(*P*)-**5**	2.00000	0.000	0
(*P*)-**6**	1.99997	0.00003	~0
(*P*)-**7**	1.99984	0.00016	~0

XYZ Coordinates

All XYZ coordinates of the DFT-calculated structures can be found in the accompanied .zip file.

10. References

[1] F. Neese, F. Wennmohs, U. Becker, C. Riplinger, *J. Chem. Phys.* **2020**, *152*, 224108.

[2] M. J. Frisch, G. W. Trucks, H. B. Schlegel, G. E. Scuseria, M. A. Robb, J. R. Cheeseman, G. Scalmani, V. Barone, G. A. Petersson, H. Nakatsuji, X. Li, M. Caricato, A. V. Marenich, J. Bloino, B. G. Janesko, R. Gomperts, B. Mennucci, H. P. Hratchian, J. V. Ortiz, A. F. Izmaylov, J. L. Sonnenberg, Williams, F. Ding, F. Lipparini, F. Egidi, J. Goings, B. Peng, A. Petrone, T. Henderson, D. Ranasinghe, V. G. Zakrzewski, J. Gao, N. Rega, G. Zheng, W. Liang, M. Hada, M. Ehara, K. Toyota, R. Fukuda, J. Hasegawa, M. Ishida, T. Nakajima, Y. Honda, O. Kitao, H. Nakai, T. Vreven, K. Throssell, J. A. Montgomery Jr., J. E. Peralta, F. Ogliaro, M. J. Bearpark, J. J. Heyd, E. N. Brothers, K. N. Kudin, V. N. Staroverov, T. A. Keith, R. Kobayashi, J. Normand, K. Raghavachari, A. P. Rendell, J. C. Burant, S. S. Iyengar, J. Tomasi, M. Cossi, J. M. Millam, M. Klene, C. Adamo, R. Cammi, J. W. Ochterski, R. L. Martin, K. Morokuma, O. Farkas, J. B. Foresman, D. J. Fox, **2016**.

[3] T. Cadart, D. Nečas, R. P. Kaiser, L. Favereau, I. Císařová, R. Gyepes, J. Hodačová, K. Kalíková, L. Bednárová, J. Crassous, M. Kotora, *Chem. – Eur. J.* **2021**, *27*, 11279–11284.

[4] L. Krause, R. Herbst-Irmer, G. M. Sheldrick, D. Stalke, *J. Appl. Crystallogr.* **2015**, *48*, 3–10.

[5] G. M. Sheldrick, *Acta Crystallogr. Sect. Found. Adv.* **2015**, *71*, 3–8.

[6] G. M. Sheldrick, *Acta Crystallogr. A* **2008**, *64*, 112–122.

[7] O. V. Dolomanov, L. J. Bourhis, R. J. Gildea, J. a. K. Howard, H. Puschmann, *J. Appl. Crystallogr.* **2009**, *42*, 339–341.

[8] M. Rickhaus, L. Jundt, M. Mayor, *CHIMIA* **2016**, *70*, 192–192.

[9] O. Trapp, *J. Chromatogr. B* **2008**, *875*, 42–47.

[10] T. Jousselin-Oba, P. E. Deal, A. G. Fix, C. K. Frederickson, C. L. Vonnegut, A. Yassar, L. N. Zakharov, M. Frigoli, M. M. Haley, *Chem. – Asian J.* **2019**, *14*, 1737–1744.

[11] C. K. Frederickson, L. N. Zakharov, M. M. Haley, *J. Am. Chem. Soc.* **2016**, *138*, 16827–16838.

[12] G. E. Rudebusch, J. L. Zafra, K. Jorner, K. Fukuda, J. L. Marshall, I. Arrechea-Marcos, G. L. Espejo, R. Ponce Ortiz, C. J. Gómez-García, L. N. Zakharov, M. Nakano, H. Ottosson, J. Casado, M. M. Haley, *Nat. Chem.* **2016**, *8*, 753–759.

[13] S. Grimme, A. Hansen, S. Ehlert, J.-M. Mewes, *J. Chem. Phys.* **2021**, *154*, 064103.

[14] V. Barone, M. Cossi, *J. Phys. Chem. A* **1998**, *102*, 1995–2001.

[15] T. Yanai, D. P. Tew, N. C. Handy, *Chem. Phys. Lett.* **2004**, *393*, 51–57.

[16] F. Weigend, R. Ahlrichs, *Phys. Chem. Chem. Phys.* **2005**, *7*, 3297–3305.

[17] A. Stanger, *J. Org. Chem.* **2006**, *71*, 883–893.

[18] A. Stanger, *J. Org. Chem.* **2010**, *75*, 2281–2288.

[19] R. Gershoni-Poranne, A. Stanger, *Chem. – Eur. J.* **2014**, *20*, 5673–5688.

[20] A. D. Becke, *J. Chem. Phys.* **1993**, *98*, 5648–5652.

[21] W. J. Hehre, R. Ditchfield, J. A. Pople, *J. Chem. Phys.* **1972**, *56*, 2257–2261.

[22] R. Krishnan, J. S. Binkley, R. Seeger, J. A. Pople, *J. Chem. Phys.* **1980**, *72*, 650–654.

[23] Rahalkar, A.; Stanger, A. "Aroma" , https://chemistry.technion.ac.il/en/team/amnon-stanger/ (accessed March 10, 2025).

[24] M. Nakano, H. Fukui, T. Minami, K. Yoneda, Y. Shigeta, R. Kishi, B. Champagne, E. Botek, T. Kubo, K. Ohta, K. Kamada, *Theor. Chem. Acc.* **2011**, *130*, 711–724.

[25] K. Yamaguchi, *Chem. Phys. Lett.* **1975**, *33*, 330–335.
